# Comparative Photoaffinity Profiling of Omega-3 Signaling Lipid Probes Reveals Prostaglandin Reductase 1 as a Metabolic Hub in Human Macrophages

**DOI:** 10.1021/jacs.2c06827

**Published:** 2022-10-05

**Authors:** Berend Gagestein, Johannes H. von Hegedus, Joanneke C. Kwekkeboom, Marieke Heijink, Niek Blomberg, Tom van der Wel, Bogdan I. Florea, Hans van den Elst, Kim Wals, Herman S. Overkleeft, Martin Giera, René E. M. Toes, Andreea Ioan-Facsinay, Mario van der Stelt

**Affiliations:** † Department of Molecular Physiology, Leiden Institute of Chemistry, Leiden University and Oncode Institute, Einsteinweg 55, Leiden 2333 CC, The Netherlands; ‡ Bio-Organic Synthesis, Leiden Institute of Chemistry, Leiden University, Einsteinweg 55, Leiden 2333 CC, The Netherlands; § Department of Rheumatology, Leiden University Medical Center, Albinusdreef 2, Leiden 2333 ZA, The Netherlands; ∥ Center for Proteomics and Metabolomics, Leiden University Medical Center, Albinusdreef 2, Leiden 2333 ZA, The Netherlands

## Abstract

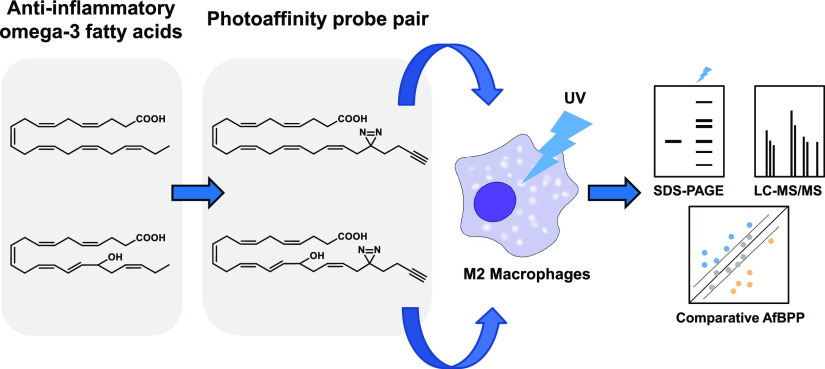

The fish oil constituent docosahexaenoic acid (DHA, 22:6 n-3) is a signaling lipid with anti-inflammatory properties. The molecular mechanisms underlying the biological effect of DHA are poorly understood. Here, we report the design, synthesis, and application of a complementary pair of bio-orthogonal, photoreactive probes based on the polyunsaturated scaffold DHA and its oxidative metabolite 17-hydroxydocosahexaenoic acid (17-HDHA). In these probes, an alkyne serves as a handle to introduce a fluorescent reporter group or a biotin-affinity tag via copper(I)-catalyzed azide-alkyne cycloaddition. This pair of chemical probes was used to map specific targets of the omega-3 signaling lipids in primary human macrophages. Prostaglandin reductase 1 (PTGR1) was identified as an interaction partner that metabolizes 17-oxo-DHA, an oxidative metabolite of 17-HDHA. 17-oxo-DHA reduced the formation of pro-inflammatory lipids 5-HETE and LTB4 in human macrophages and neutrophils. Our results demonstrate the potential of comparative photoaffinity protein profiling for the discovery of metabolic enzymes of bioactive lipids and highlight the power of chemical proteomics to uncover new biological insights.

## Introduction

**Scheme 1:**
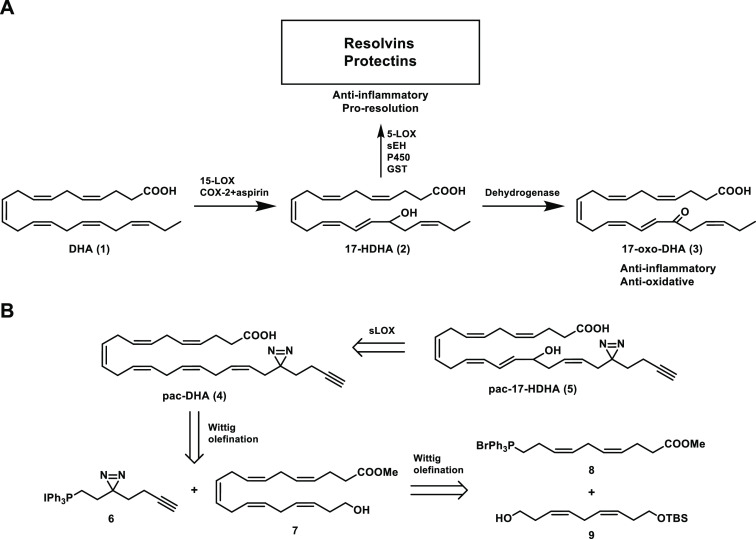
Oxidative Metabolism of DHA (1) and Synthetic Strategy of Photoaffinity Probes **4** and **5** (A) Metabolic pathway of DHA to 17-oxo-DHA, resolvins, and protectins. (B) Structures of photoaffinity-click (pac-)probes based on DHA (1) and 17-HDHA (2). The synthetic strategy included the use of soybean lipoxygenase (sLOX) to introduce the hydroxyl and the use of Wittig reactions to join building blocks 6, 8, and 9. COX, cyclooxygenase; DHA, docosahexaenoic acid; GST, glutathione S-transferase; HDHA, hydroxydocosahexaenoic acid; LOX, lipoxygenase; P450, cytochrome P450; sHE, soluble epoxide hydrolase.

Dietary omega-3 polyunsaturated fatty acids (PUFAs) are generally considered to be beneficial for human health.^1−3^ For example, the fish oil constituent docosahexaenoic acid (DHA, 22:6 n-3, Scheme 1A) is important for brain development, cardiovascular function, and the immune system.^2−7^ Mechanistic studies indicate that many of the favorable effects of omega-3 fatty acids are due to their interaction with immunological processes,^1,2^ which has been reiterated by the discovery of their oxidized metabolites involved in the resolution of inflammation.^8,9^ Malfunction of this resolution phase of inflammation is hypothesized to contribute to many chronic inflammatory diseases, such as rheumatoid arthritis and asthma.^10,11^ A better molecular understanding of the biological role of these lipids in the resolution phase of inflammation is required to develop therapeutics for these diseases.

The resolution of inflammation requires the intricate orchestration of cells of the innate immune system via soluble mediators. Among these, oxidative metabolites of DHA play a central role.^7,12^ Biochemical studies have shown that DHA is oxidized into a central precursor, 17-hydroxydocosahexaenoic acid (17-HDHA, **2**) via multiple pathways, including 15-lipoxygenase and cyclooxygenase acetylated by aspirin (Scheme 1A).^13^ Oxidation by 15-lipoxygenase results in the formation of 17(*S*)-HDHA, while acetylated COX generates 17(*R*)-HDHA.^14^ Importantly, 17-HDHA has protective effects in various animal models of colitis, arthritis, and renal reperfusion.^15−18^ In vitro, human macrophages produce less TNFα and more IL-10 following exposure to 17-HDHA.^19,20^ Treatment with 17-HDHA also reduces LTB4 production in both isolated murine and human neutrophils.^20,21^ Moreover, 17-HDHA can be further metabolized to specialized pro-resolving lipid mediators (SPMs), such as D-series resolvins and protectins (Scheme 1A), a process involving several enzymes. Resolvins and protectins are bioactive lipids with potent pro-resolving activities, including halting the infiltration of neutrophils and enhancing the non-phlogistic clearance of apoptotic cells, cellular debris, and microbes by macrophages, thereby stimulating the resolution of inflammation, while promoting tissue regeneration.^8,22^ On the other hand, 17-HDHA can be metabolized to 17-oxo-docosahexaenoic acid (17-oxo-DHA, **3**), which may limit the formation of SPMs. Insight into its protein interaction partners in human immune cells would be of great benefit in obtaining a better molecular understanding of the biological role and metabolism of DHA and its oxidative metabolites.

Lipid photoaffinity probes have been successfully used to map protein–lipid interactions on a global scale in their native environment.^23^ Bio-orthogonal photoaffinity lipid probes consist of a lipid modified with a photoreactive group and bio-orthogonal ligation handle.^24^ The photoreactive group is activated by irradiation with light, generating a reactive species that may form a covalent and irreversible bond with the interacting protein. The ligation handle is used to attach a reporter group via bio-orthogonal chemistry, which allows for visualization or isolation of the probe-bound protein in a complex biological sample. This affinity-based protein profiling (AfBPP) approach has been reported for multiple lipid classes, including phospholipids,^25,26^ fatty acids,^23,27^ sphingolipids,^28,29^ and sterols,^24,30^ but has not been applied to omega-3 PUFAs, arguably due to the synthetic challenges associated with their preparation.^31^

An important drawback of photoreactive lipid-based probes is their inherent high lipophilicity and nonspecific binding to proteins. Significant overlap between protein targets of lipid probes has been documented, making it difficult to assign specific interaction partners to a given probe and to study their biological role.^32,33^ Competition experiments with non-labeled lipids have been applied to identify specific binding partners but were not always successful, possibly due to the accumulation of both probe and competitor lipids in the cellular membrane.^34,35^

**Figure 1:**
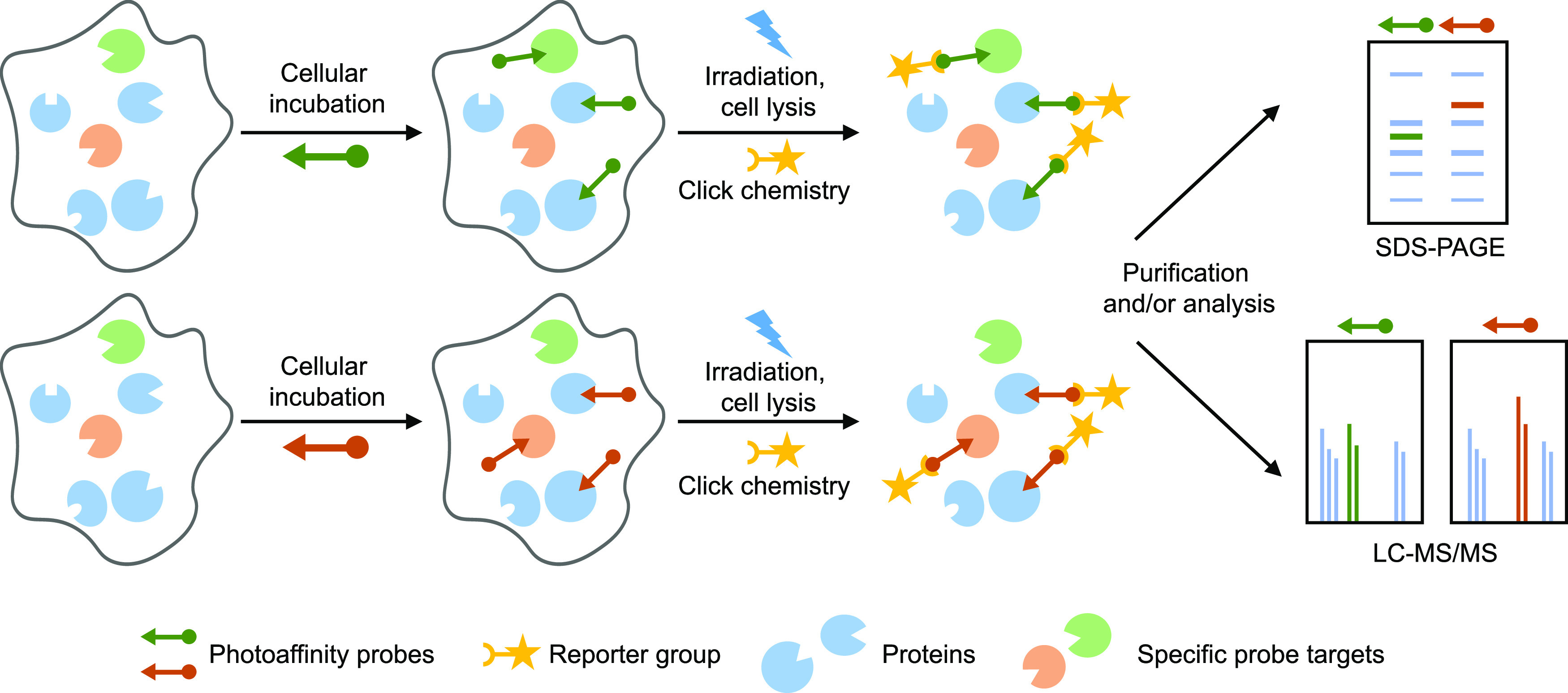
Schematic overview of the comparative photoaffinity-based protein profiling (AfBPP) experiment. Two highly similar, bifunctional photoreactive probes are used to identify probe-interacting proteins using chemical proteomics. Cells are supplied with either probe, and after an incubation period, the photoreactive group is activated using UV light, which results in the formation of a covalent and irreversible bond with the probe-interacting protein. The probe-bound protein can then be investigated by ligation to a fluorescent group and SDS-PAGE separation or ligation to an affinity handle, enriched, and identified using LC–MS/MS. Probe-specific targets can be identified by comparing the interaction landscapes of the individual probes.

Here, we describe the design, synthesis, and application of a pair of complementary photoaffinity probes (**4** and **5**) based on the structure of DHA and 17-HDHA, respectively (Scheme 1B). Our main aim was to map specific lipid targets of 17-HDHA in primary human macrophages by comparative AfBPP (Figure 1). Probe **5** retained the anti-inflammatory properties of the parent lipid in human M2 macrophages. Using chemical proteomics, we identified prostaglandin reductase 1 (PTGR1) as a lipid-binding partner. Subsequent, biochemical and genetic studies revealed that PTGR1 metabolizes 17-oxo-DHA, an oxidative metabolite of 17-HDHA. 17-oxo-DHA reduced the biosynthesis of the pro-inflammatory lipid mediators 5-HETE and LTB4 in human macrophages and neutrophils. Thus, comparative photoaffinity labeling revealed PTGR1 as a metabolic hub in bioactive lipid signaling in human macrophages.

## Results

### Design and Synthesis of Photoaffinity Probes **4** and **5**

In the design of photoaffinity probes **4** and **5** (Scheme 1B) and to ensure they closely resemble the signaling lipids DHA and 17-HDHA, respectively, we decided to keep the PUFA scaffold intact and substitute the omega carbon with a diazirine- and alkyne-containing minimalistic bifunctional group.^36^ Diazirines are small photoreactive groups with short reactive half-lives upon activation, thereby minimizing the interference and reducing non-specific labeling. The alkyne is the smallest bio-orthogonal tag available and has physio-chemical properties similar to the alkyl chain of fatty acids.^37,38^

To synthesize the probes in an efficient manner, we made use of a chemoenzymatic approach. Probe **5** was produced by soybean lipoxygenase using probe **4** as the substrate (Scheme 1B). Probe **4** was synthesized by combining two strategic building blocks, the minimalistic bifunctional photoreactive linker **6** and the PUFA scaffold (**7**), using a Wittig reaction. Building block **7** was generated by combining the two dienes **8** and **9**, also via a Wittig reaction. This synthetic strategy avoids the reduction of six skipped (non-conjugated) alkynes at once, which would lead to a complex mixture of partially hydrogenated products.^39^ Moreover, this strategy did not require the assembly of large, skipped polyalkyne structures, which are tremendously unstable.^40,41^ Moreover, this strategy allowed the installation of the labile diazirine at a late stage in the synthesis.

**Scheme 2:**
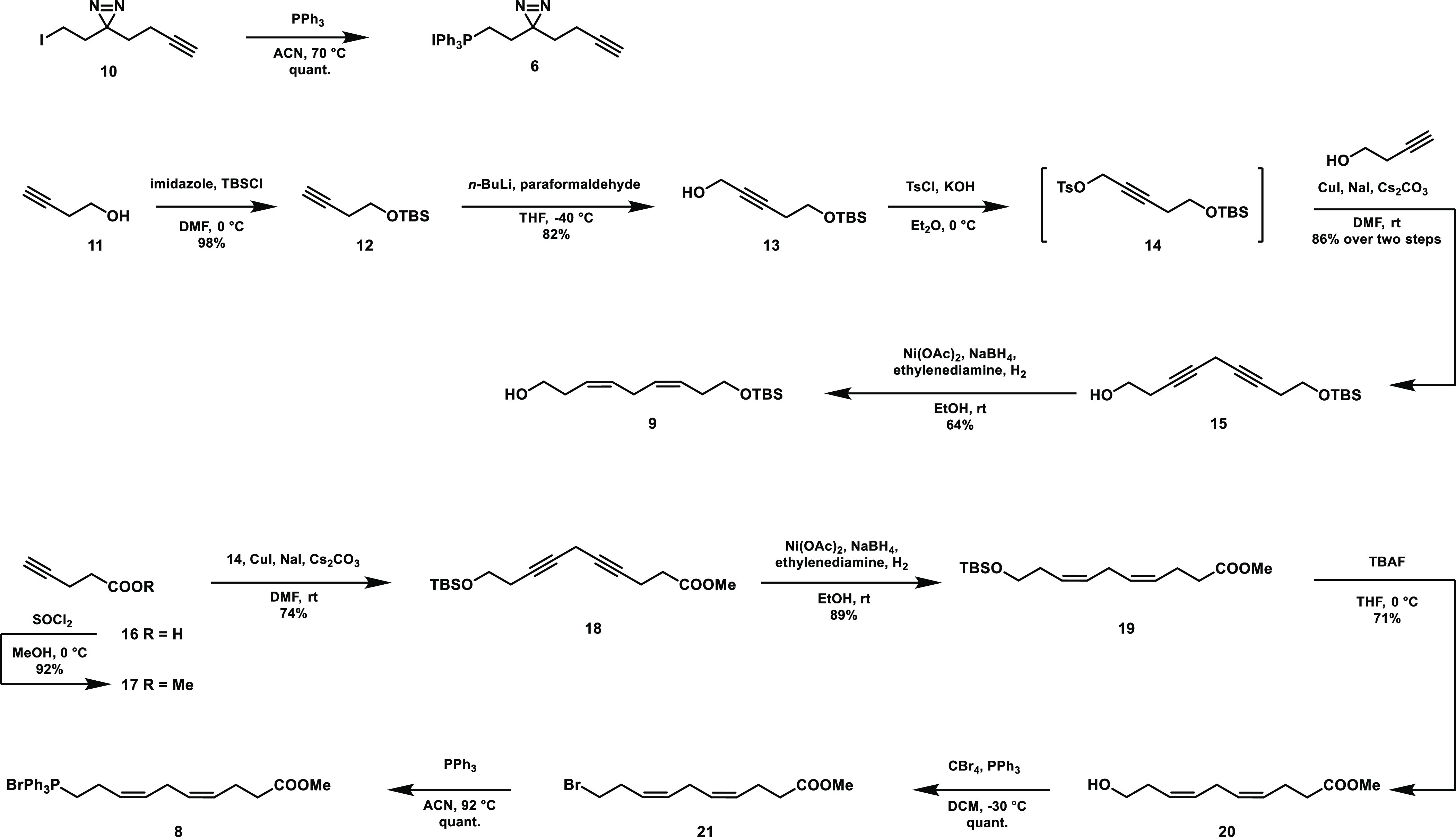
Synthesis of Building Blocks **6**, **8**, and **9**

First, minimalist linker derivative **10** was synthesized following a previously reported route^36^ with minor modifications (Scheme S1). To enable its use in a Wittig reaction, the iodide was substituted with triphenylphosphine at 70 °C to obtain phosphonium salt **6** in quantitative yield (Scheme 2). To obtain fragments **8** and **9**, skipped alkynes were assembled and partially hydrogenated using P-2 nickel. To this end, butynol (**11**) was protected with a tert-butyl(dimethyl)silyl (TBS) group to afford alkyne **12** in 98% yield. This compound was deprotonated using *n*-butyllithium and reacted with paraformaldehyde to afford alcohol **13**, which was tosylated to obtain **14**. This intermediate was used without further purification for a copper(I)-mediated coupling to butynol to afford skipped alkyne **15** in 71% yield over three steps. The skipped alkyne was partially hydrogenated using P-2 nickel catalyst to furnish alcohol **9** in 64% yield.

For fragment **8**, pentynoic acid (**16**) was protected by esterification in methanol to obtain **17**, which was reacted with sulfonate ester **14** in a second copper(I)-mediated coupling to afford skipped alkyne **18** (Scheme 2). Skipped alkyne **18** was partially hydrogenated to afford silylated **19**. The TBS group was removed using tetrabutylammonium fluoride (TBAF) to afford alcohol **20**, which was halogenated to form **21**. This was then substituted with triphenylphosphine to afford phosphonium salt **8** in 43% yield from pentynoic acid.

**Scheme 3:**
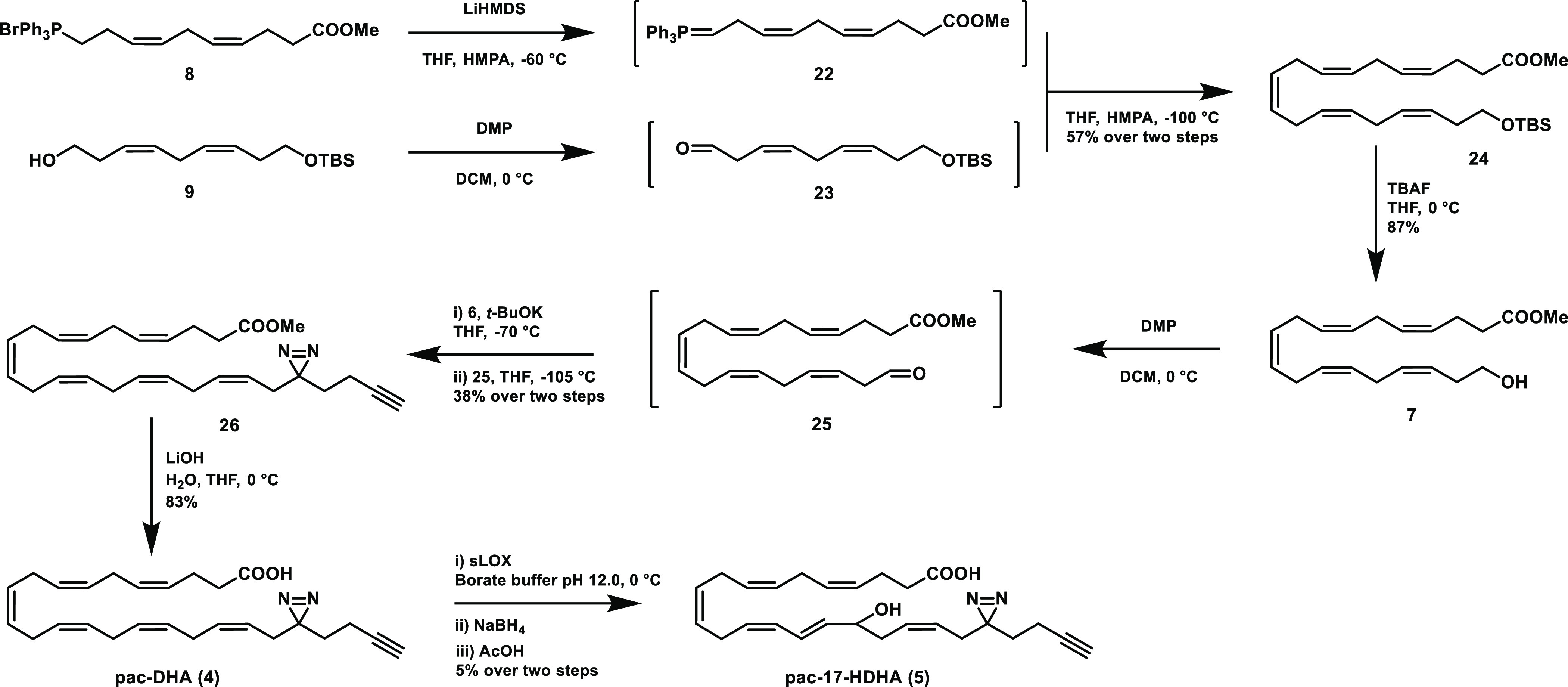
Formation of Probes **4** and **5** by Assembly of Building Blocks **6**, **8**, and **9**

To join intermediates **8** and **9**, a Wittig reaction was performed by deprotonation of **8** to form ylid **22** using lithium bis(trimethylsilyl)amide (LiHMDS) at a reduced temperature, and the addition of freshly prepared aldehyde **23** at −100 °C (Scheme 3). Aldehyde **23** was prepared by oxidation of alcohol **9** using DMP directly before use due to the instability of β,γ-unsaturated aldehydes.^42,43^ The Wittig reaction afforded with high preference the *Z*-isomer, which could be separated from the undesired *E*-isomer by column chromatography to afford **24** in 57% yield. **24** was in turn deprotected using TBAF to obtain alcohol **7** in 87% yield.

Building block **6** was installed in probe **4** by generating the final double bond using another Wittig reaction, for which alcohol **7** was oxidized with DMP, while simultaneously treating phosphonium salt **6** with KO*t*Bu at a reduced temperature. Higher temperatures or stronger bases resulted in the loss of the diazirine. The freshly generated aldehyde was added to the ylid at −105 °C, which afforded methyl ester **26** in 38% yield after workup and purification by column chromatography, which was capable of removing the *E*-isomer. The *Z*-configuration of the last double bond was checked by NMR, where a *Z*-characteristic coupling constant of 10.4 Hz was found. Saponification of the ester in **26** yielded photoaffinity probe **4** in 83% yield.

To generate the desired photoaffinity-click (pac)-17-HDHA (**5**), commercially available soybean lipoxygenase (sLOX) was used, which catalyzes the oxidation of DHA (**1**) to 17-hydroperoxy-DHA, which can be reduced to 17-HDHA (**2**).^44,45^ While DHA (**1**) was fully converted into 17-HDHA by sLOX using previously reported conditions,^46^ this resulted in the complete loss of the diazirine when using probe **4** as a substrate. To this end, the reaction conditions with probe **4** were optimized for enzyme loading, temperature, and incubation time. This resulted in the formation of probe **5**, which could be obtained after high-performance liquid chromatography (HPLC) purification. The position of the hydroxyl group was confirmed by fragmentation on LC–MS after hydrogenation of the alkenes using previously reported conditions.^47^ Of note, the final enzymatic step yielded only a small amount of probe **5**, which was sufficient for subsequent biological experiments. Further optimization of reaction conditions or modification of the lipoxygenase enzyme may improve the yield, although the latter strategy comes with risk, as changing the lipoxygenase structure is known to change its positional specificity.^48,49^

### Mapping Protein Interaction Partners of Probes **4** and **5**

**Figure 2:**
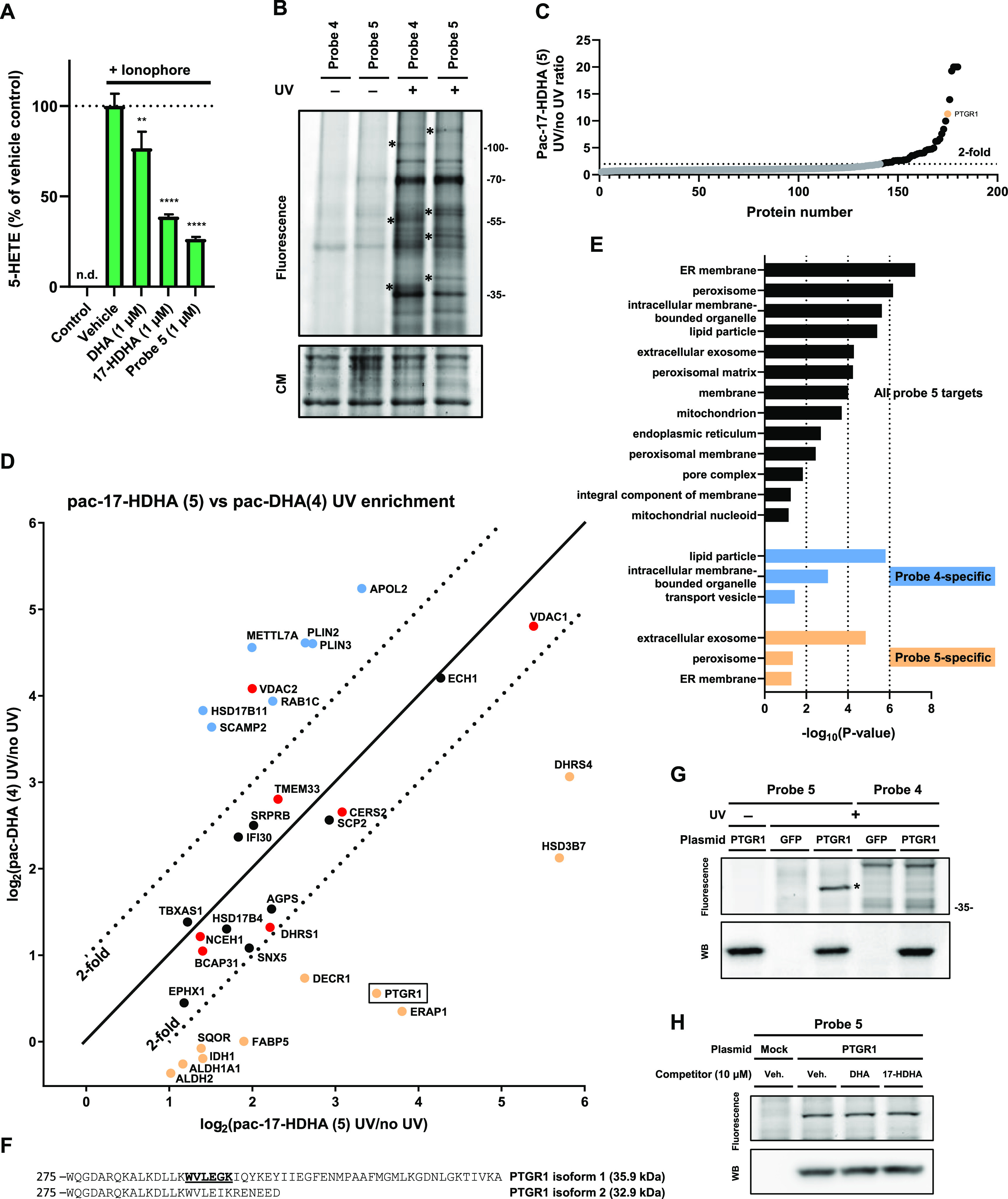
Comparative ABPP in human M2 macrophages using pac-17-HDHA and pac-DHA. (A) 5-HETE formation (LC–MS/MS) produced by ionophore-stimulated M2 macrophages. Data represent means ± SD of cells obtained from a representative donor (*n* = 3). **p < 0.01, ****p < 0.0001 in comparison to ionophore-treated control using a one-way ANOVA with Dunnett’s multiple comparisons correction. n.d.: not detected. (B) Probe targets of pac-17-HDHA (**5**) and pac-DHA (**4**) conjugated to Cy5-N_3_ using CuAAC chemistry and analyzed by SDS-PAGE and in-gel fluorescent scanning show UV-dependent and probe-specific labeling. Coomassie (CM) served as a protein loading control. Asterisks indicate probe-specific bands. (C) Waterfall plot of proteins identified using probe **5** in M2 macrophages. UV enrichment is capped at 20-fold. (D) UV enrichment of probe **5**-enriched targets by probe **4** and probe **5** is shown, with a 2-fold cutoff indicating probe-specific targets, with probe **4**-specific targets in blue and probe **5**-specific targets in orange. Promiscuous lipid probe binders^33^ are indicated in red. Data represent means ± SD of cells obtained from a representative donor (*n* = 3). (E) Cellular component analysis of probe **5**-interacting proteins by gene ontology (GO). (F) PTGR1 amino acid sequence starting at tryptophan W275. One of the identified tryptic peptides is underlined and bold. (G, H) Gel-based AfBPP of GFP- or PTGR1-overexpressing HEK-293-T cells using probes **4** and **5** and indicated a lipid competitor. Expression of PTGR1 was checked by anti-FLAG western blot.

Since our main aim was to identify specific binding partners of 17-HDHA, probe **5** was tested in a cellular assay using primary human M2 macrophages to confirm it was biologically equivalent to 17-HDHA in reducing the inflammatory response. M2 macrophages were differentiated from monocytes of healthy donors in the presence of macrophage colony-stimulating factor (M-CSF). Upon stimulation with Ca^2+^ ionophore, M2 macrophages rapidly synthesize the proinflammatory eicosanoid 5-HETE from arachidonic acid (AA) by 5-lipoxygenase (Figure 2A). A liquid chromatography–mass spectrometry (LC–MS) method was developed to quantify 5-HETE in these human macrophages. Pre-incubation with 17-HDHA lipid (1 μM) or probe **5** (1 μM) significantly reduced the conversion of AA into 5-HETE. DHA, which was taken along as a negative control compound, was less effective (Figure 2A), thereby confirming the specificity of the 17-HDHA response. In conclusion, probe **5** mimics the anti-inflammatory signaling capacity of 17-HDHA in human macrophages and can be used to investigate the binding partners of its parent lipid.

Next, we investigated the protein interaction landscape of probe **5** in human M2 macrophages using two-step AfBPP. To identify specific targets of 17-HDHA, we also employed probe **4** to map general, promiscuous lipid binding proteins binding with equal preference to both probes. To this end, cells were incubated with probe **4** or **5** in serum-free medium for 30 min to allow for sufficient uptake. Crosslinking was effected by UV-irradiation (λ = 350 nm, 10 min) using a CaproBox,^50^ which irradiated the cells with simultaneous cooling at 4 °C to counteract the heat induced by the irradiation. Next, the cells were harvested, lysed, and subjected to copper(I)-catalyzed azide-alkyne cycloaddition (CuAAC, “click”-reaction)^51^ conditions utilizing Cy5-N_3_ to enable the visualization of the probe-bound proteins by sodium dodecyl sulfate polyacrylamide gel electrophoresis (SDS-PAGE) analysis and in-gel fluorescence scanning (Figure 2B). This resulted in the visualization of many fluorescent bands for both probes, which were absent in the non-irradiated samples, demonstrating that the probes do not covalently interact with proteins without UV irradiation. Although a large overlap in fluorescent bands was revealed after labeling by either probe **4** or **5**, there were also several probe-specific bands observed (Figure 2B).

To identify the probe-interacting proteins, a chemical proteomics experiment was performed. Label-free quantification was used in order to compare the relative abundance of probe-bound proteins in the samples.^52^ Briefly, the tagged proteins were ligated to biotin-N_3_ via click chemistry, and the resulting biotin-labeled proteins were enriched using avidin-coated agarose beads and digested using trypsin and the resulting tryptic peptides were analyzed by LC–MS using a Synapt G2-Si instrument. The data processing was performed with the commercial software Progenesis, which resulted in the identification and quantification of 179 proteins after deselection based on identified unique peptides and appearance in the CRAPome.^53^ Of these, 34 were significantly UV-enriched by probe **5** (Figure 2C,D). To identify probe **5**-specific interacting proteins, the UV enrichment profiles of both probes were compared (Figure 2D). Proteins that were previously identified as promiscuous lipid probe binders^33^ are indicated in red. All promiscuous lipid probe binders, with the singular exception of voltage-dependent anion channel 2 (VDAC2), were equally enriched by both probes. Eight and ten proteins were specifically labeled by probes **4** and **5**, respectively. This demonstrated that comparative AfBPP is capable of discerning probe-specific interactions.

Gene ontology (GO) enrichment analysis revealed that the proteins significantly UV-enriched by probe **5** are mainly found in organelles in lipid metabolism, such as the endoplasmic reticulum and mitochondria (Table 1). On the other hand, probe **4**-specific targets are associated with lipid particles and transport vesicles, such as perilipin-2 and -3, which are involved in lipid droplet formation. This may suggest DHA is incorporated in lipid droplets. Of note, probe-**5**-specific targets were significantly associated with the extracellular exosomes GO term (Figure 2E)^54^ and predominantly annotated as oxidoreductase enzymes, for example, aldehyde dehydrogenase 1A1 (ALDH1A1) and prostaglandin reductase-1 (PTGR1). The main activity of ALDH1A1 consists of producing the signaling lipid retinoic acid by oxidation of retinaldehyde, but it can also react with other aldehydes such as 4-hydroxy-2-nonenal (4-HNE).^55,56^ Specific binding of probe **5** indicates ALDH1A1 could be involved 17-HDHA metabolism, possibly via a reactive aldehyde metabolite analogous to 4-hydroxy-2-nonenal. PTGR1 is known for its role in eicosoanoid and 4-HNE metabolism.^57,58^ PTGR1 expression is increased during inflammation and is involved in the resolution of inflammation through modulation of the HMGB1-miR522-3P-PTGR1 axis.^59^ PTGR1 functions as 15-oxo-prostaglandin 13-reductase and acts on 15-oxo-PGE1 and 15-oxo-PGE2.^57,59^ It also catalyzes the conversion LTB4 into its biologically less active metabolite, 12-oxo-LTB4, which is an initial and key step of metabolic inactivation of LTB4.^60^

**Table 1:** List of all Proteins Significantly UV-Enriched by Probe **5** and Their Probe Specificity

Uniprot accession	Gene name	Unique peptides	Description	Specific
Q9H2F3	HSD3B7	3	3 beta-hydroxysteroid dehydrogenase type 7	probe **5**
Q9NZ08	ERAP1	20	endoplasmic reticulum aminopeptidase 1	probe **5**
Q14914	PTGR1	11	prostaglandin reductase 1	probe **5**
Q9BTZ2	DHRS4	10	dehydrogenase/reductase SDR family member 4	probe **5**
Q16698	DECR1	4	2,4-dienoyl-CoA reductase, mitochondrial	probe **5**
Q01469	FABP5	2	fatty acid-binding protein, epidermal	probe **5**
O75874	IDH1	8	isocitrate dehydrogenase [NADP] cytoplasmic	probe **5**
Q9Y6N5	SQOR	6	sulfide:quinone oxidoreductase, mitochondrial	probe **5**
P00352	ALDH1A1	17	retinal dehydrogenase 1	probe **5**
P05091	ALDH2	16	aldehyde dehydrogenase, mitochondrial	probe **5**
				
Q92928	RAB1C	2	putative Ras-related protein Rab-1C	probe **4**
Q99541	PLIN2	11	perilipin-2	probe **4**
Q9BQE5	APOL2	4	apolipoprotein L2	probe **4**
O60664	PLIN3	2	perilipin-3	probe **4**
P45880	VDAC2	7	voltage-dependent anion-selective channel protein 2	probe **4**
O15127	SCAMP2	2	secretory carrier-associated membrane protein 2	probe **4**
Q8NBQ5	HSD17B11	2	estradiol 17-beta-dehydrogenase 11	probe **4**
Q9H8H3	METTL7A	2	methyltransferase-like protein 7A	probe **4**

### Validation of Probe **5**-Specific Targets and Role of PTGR1 in 17-HDHA Metabolism

**Figure 3:**
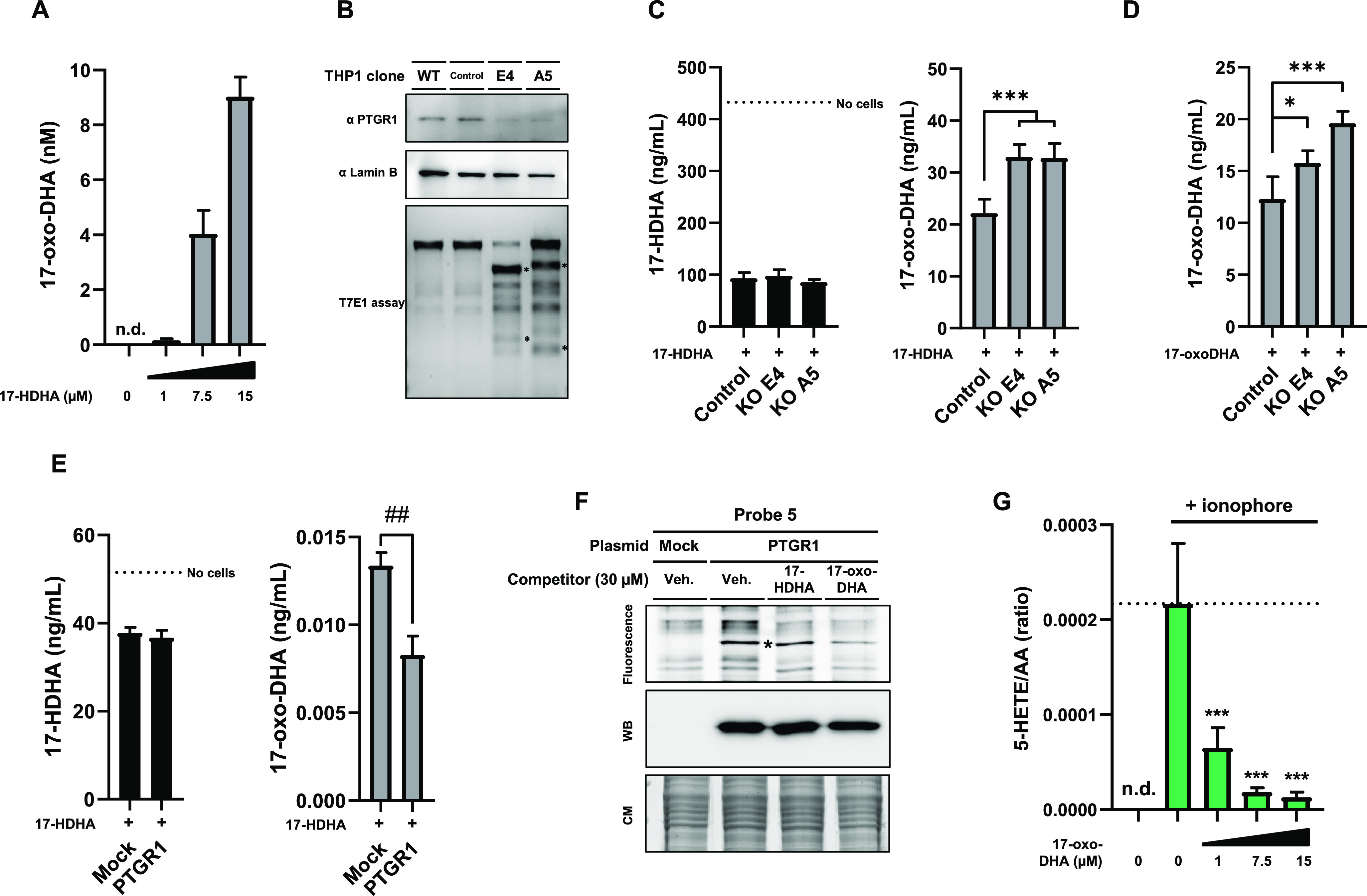
Investigation of 17-HDHA to 17-oxo-DHA metabolism and anti-inflammatory signaling. (A) 17-Oxo-DHA produced by M2 macrophages. Cells were either pretreated for 2 h with 17-HDHA or vehicle control. Data represent means ± SD of cells obtained from a representative donor (*n* = 3). (B) Characterization of PTGR1 CRISPR/Cas9 knockout THP1 cells. Single-cell clones were generated of which two were further cultured. (C) 17-Oxo-DHA produced by WT or PTGR1 KO THP1 cells treated with 1 μM 17-HDHA for 4 h. (D) 17-Oxo-DHA remaining after treating WT or PTGR1 KO THP1 cells with 1 μM 17-oxo-DHA for 3 h. (E) 17-HDHA and 17-oxo-DHA levels of U2OS cells overexpressing PTGR1 or control. Cells were treated for 4 h with 300 nM 17-HDHA. (F) Gel-based AfBPP of control or PTGR1-overexpressing HEK-293-T cells using probes **4** and **5** and indicated a lipid competitor. Expression of PTGR1 was checked by anti-FLAG western blot. Asterisk indicates PTGR1 band. (G) 5-HETE/AA ratio (LC–MS/MS) produced by ionophore-stimulated M2 macrophages. Cells were either pretreated for 15 min with 17-oxo-DHA or vehicle control. Data from THP-1 and U2OS cells represent means ± SD (*n* = 3–4). *p < 0.05, **p < 0.01, ***p < 0.001 in comparison to control (dotted line) using a one-way ANOVA with Dunnett’s multiple comparisons correction. ^##^*p* < 0.01 using a Student’s *t*-test. n.d.: not detected.

Two probe **5**-enriched proteins, PTGR1 (isoform 1, Figure 2F) and ALDH1A1, were chosen as representative targets for validation as 17-HDHA interaction partners (Figure 2F). Both proteins were recombinantly expressed with a FLAG-tag in HEK-293T cells. Cells were treated with either probe **4** or **5**, irradiated, and lysed, and probe-labeled proteins were ligated to Cy5-N_3_ using click chemistry. The proteins were resolved on SDS-PAGE gel and visualized with a fluorescent scanner (Figures 2G and S1). A fluorescent band was observed in a UV-dependent manner at the expected molecular weight for PTGR1 and ALDH1A1 in the samples treated with probe **5**, but not by probe **4**. The fluorescent band overlapped with a FLAG-tag signal in the western blot. Next, we investigated whether the labeling of PTGR1 could be outcompeted by 17-HDHA or DHA. Both lipids did not prevent the labeling of PTGR1 by probe **5** (Figure 2H). Inherent high lipophilicity, low aqueous solubility, and partitioning into the cellular membrane may explain why target occupancy studies are challenging with lipids.^34,35^ Alternatively, it is conceivable that probe **5** was converted into an active metabolite, such as a 17-oxo-derivative, that labels PTGR1. To investigate this hypothesis, we first tested if primary human M2 macrophages and human THP-1 cells are capable of forming 17-oxo-DHA using 17-HDHA as a substrate. To this end, a targeted lipidomics method was set up to quantify 17-oxo-DHA. Upon incubation of M2 macrophages and THP-1 cells with 17-HDHA, we could detect the formation of 17-oxo-DHA in a concentration-dependent manner (Figure 3A). To study whether PTGR1 uses 17-HDHA or 17-oxo-DHA as a substrate, we genetically deleted PTGR1 from THP-1 cells by CRISPR/Cas9. Two different KO clones were generated and treated with 17-HDHA or 17-oxo-DHA (Figures 3B and S3). It was found 17-oxo-DHA levels increased in the KO cells compared to wildtype cells, whereas 17-HDHA levels remained the same (Figure 3C,D). Conversely, overexpressing PTGR1 in U2OS cells resulted in lower 17-oxo-DHA levels, but 17-HDHA concentrations were not affected upon incubation with 17-HDHA (Figure 3E). To determine whether 17-oxo-DHA directly interacted with PTGR1, we performed a competition experiment with probe **5** on recombinant PTGR1 expressed in HEK293T cells. It was observed that 17-oxo-DHA could reduce the PTGR1 labeling by probe **5** (Figure 3F). Next, it was tested if PTGR1 could use 17-oxo-DHA as a substrate. Incubation of 17-oxo-DHA with purified, recombinant PTGR1 led to the conversion of the lipid into a metabolite with a mass of 17-oxo-DHA plus two hydrogen atoms in a temperature- and protein-dependent manner (Figure S5). Likely, the 15-*cis* double bond was reduced by the analogy of the action of PTGR1 on prostaglandins. Taken together, these results indicate that 17-oxo-DHA is a substrate of PTGR1.

Finally, to investigate whether 17-oxo-DHA has anti-inflammatory effects, primary human M2 macrophages and neutrophils were stimulated with an ionophore with or without 17-oxo-DHA present. Notably, dose-dependent inhibition of the pro-inflammatory markers in both macrophages (Figure 3G) and neutrophils (Figure S4) was observed, thereby indicating that 17-oxo-DHA may limit the inflammatory response.

## Conclusions

Here, we developed a comparative chemical proteomic approach with two complementary bifunctional photoreactive probes **4** and **5**, which were based on the scaffold of the PUFA DHA and its oxidative metabolite 17-HDHA. Our aim was to identify specific targets of 17-HDHA. The probes were synthesized by forming building blocks containing four of the alkenes of DHA by partial hydrogenation and combining them by Wittig reactions, forming the last two double bonds. The final step in the synthesis of probe **5** was performed by employing an enzymatic reaction using soybean lipoxygenase.

Using both probes in primary human macrophages from healthy human donors, we identified several probe-specific lipid–protein interactions. PTGR1 was selected as a representative example for validation as a protein target of 17-HDHA. PTGR1 is known for its role in eicosoanoid and 4-HNE metabolism but was so far not linked to omega-3 lipid metabolism.^57,58^ PTGR1 is involved in immune signaling by inactivation of pro-inflammatory eicosanoids such as prostaglandins and LTB4. Here, we have shown that PTGR1 also uses 17-oxo-DHA as a substrate, likely by acting as a 15-reductase. Since 17-oxo-DHA reduces the formation of pro-inflammatory markers 5-HETE and LTB4, its metabolism by PTGR1 may limit this anti-inflammatory effect. Thus, PTGR1 is simultaneously metabolizing pro- and anti-inflammatory lipids. Further studies may shed light on the biological role of the novel metabolite generated by PGTR1.

Altogether, our study extends previous reports, suggesting that PTGR1 serves as a metabolic hub that inactivates proinflammatory LTB4 and metabolizes anti-inflammatory 17-oxo-DHA, thereby modulating the cellular levels of these important signaling lipids that act in concert to modulate the human macrophage–neutrophil axis. Finally, our results highlight the use of complementary bifunctional, photoreactive probes to identify specific protein interaction partners of promiscuous, lipophilic signaling molecules and also showcase the power of chemical proteomics in guiding the discovery of novel biological insights in primary human cells.

## Supporting Information Available

Supplementary Figures S1–S6, experimental procedures and spectra (PDF)
Proteomics output (XLSX)

## Notes

The mass spectrometry proteomics data have been deposited to the ProteomeXchange Consortium via the PRIDE^61^ partner repository with the dataset identifier PXD027600.
