# Supplementary material for: Comparative Photoaffinity Profiling of Omega-3 Signaling Lipid Probes Reveals Prostaglandin Reductase 1 as a Metabolic Hub in Human Macrophages
Source: J Am Chem Soc. 2022 Oct 5;144(41):18938–47. doi: 10.1021/jacs.2c06827 (PMC9585591; doi:10.1021/jacs.2c06827)
Supplement: Supplementary file 1 — ja2c06827_si_001.pdf [file ja2c06827_si_001.pdf]

Supporting information for

Comparative photoaffinity profiling of omega-3 signaling lipid probes reveals prostaglandin reductase 1 as a metabolic hub in human macrophages

Berend Gagestein[1,†], Johannes H. von Hegedus[3,†], Joanneke C. Kwekkeboom[3], Marieke Heijink[4], Niek Blomberg[4], Tom van der Wel[1], Bogdan I. Florea[2], Hans van den Elst[2], Kim Wals[1], Herman S. Overkleeft[2], Martin Giera[4], René E. M. Toes[3], Andreea Ioan-Facsinay[3], Mario van der Stelt[1,*]

[1]Department of Molecular Physiology, Leiden Institute of Chemistry, [2]Bio-Organic Synthesis, Leiden Institute of Chemistry, Leiden University, Einsteinweg 55, Leiden 2333 CC, The Netherlands, [3]Department of Rheumatology, Leiden University Medical Center, [4]Center for Proteomics and Metabolomics, Leiden University Medical Center, Albinusdreef 2, Leiden 2333 ZA, The Netherlands. *Correspondence should be addressed to M. van der Stelt (m.van.der.stelt@chem.leidenuniv.nl). † B.G. and J.H.H. contributed equally to this work.

Table of contents

Supplemental schemes and figures 3
Figure S1. 3
Figure S2. 4
Figure S3. 4
Figure S4. 4
Figure S5. 5
Figure S6. 6
Supplemental methods 7
General remarks 7
Cloning 7
Cell culture 7
Transfection of HEK-293-T cells 7
CRISPR/Cas9 KO generation 7
Table S1. 9
Gel-based AfBPP of HEK-293-T cells 9
Macrophage and neutrophil isolation and treatment 9
THP1 treatment for lipidomics 10
Lipid isolation and LC-MS/MS 10
In vitro LC-MS-based 17-oxo-DHA conversion assay 11
AfBPP of M2 macrophages 11
Mass spectrometric analysis of tryptic peptides, identification and quantification 11
Table S2. 12
Statistical analysis 12
Synthesis 13
^{1}H and ^{13}C NMR spectra 21
LC-MS analysis of pac-17-HDHA (5) 45
Fragmentation pattern of hydrogenated pac-17-HDHA (5) 46
References 47

Supplemental schemes and figures

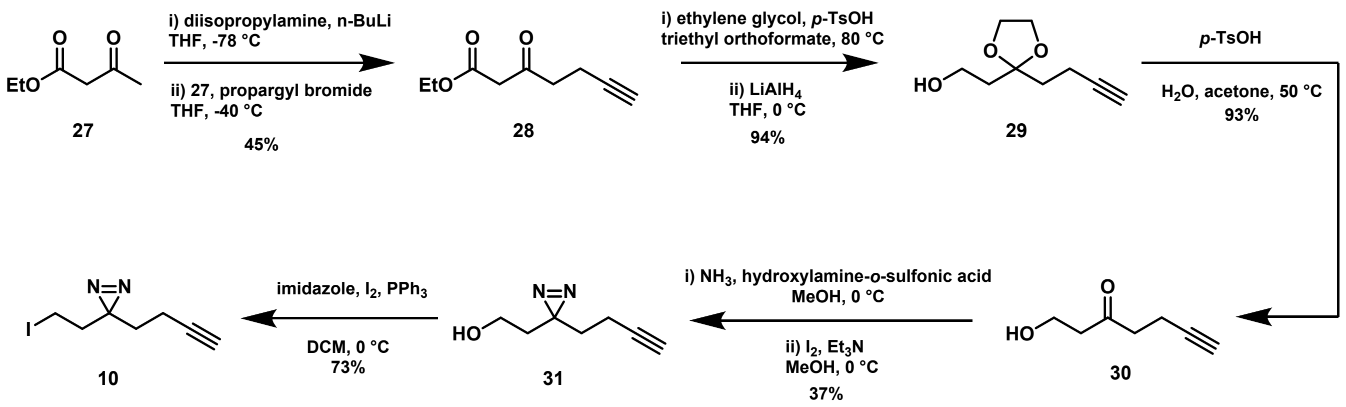

Scheme S1. Synthesis of minimalist diazirine linker **10**.

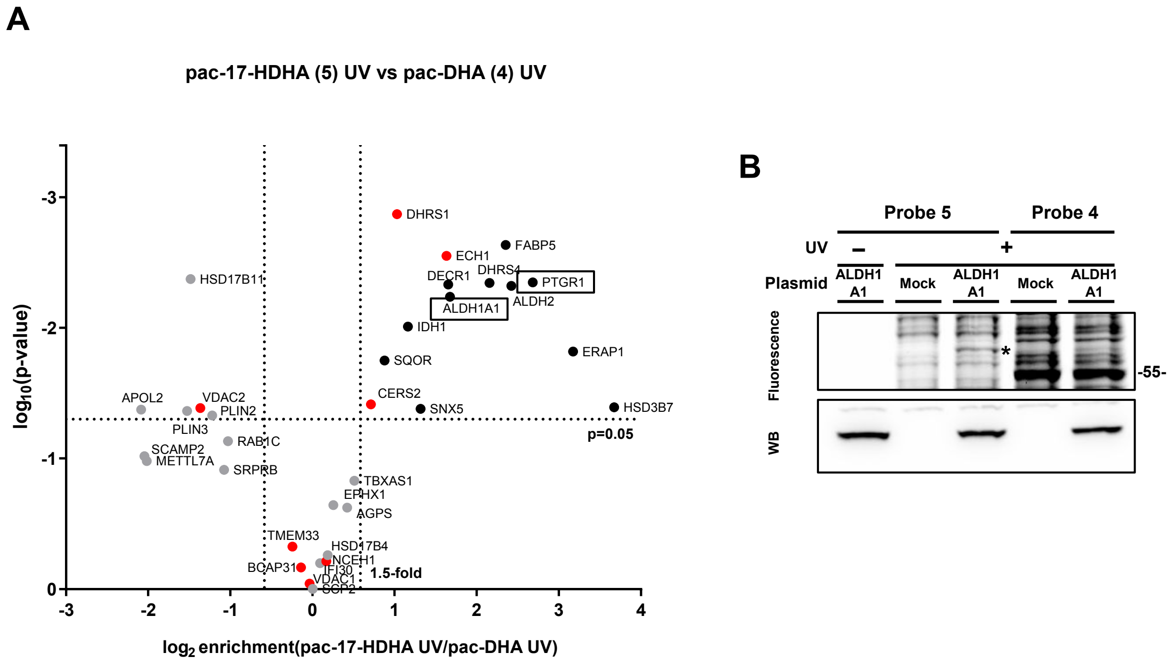

Figure S1. (A) Volcano plot of UV enrichment of probe **5**-enriched targets by probe **4** and probe **5**. Dotted lines indicate a 1.5-fold preference for either probe and p = 0.05. Promiscuous lipid probe binders[1] are indicated in red. **(B)** Gel-based AfBPP of control or ALDH1A1-overexpressing HEK-293-T cells using probe **4** and **5**. Expression of ALDH1A1 was checked by anti-FLAG western blot.

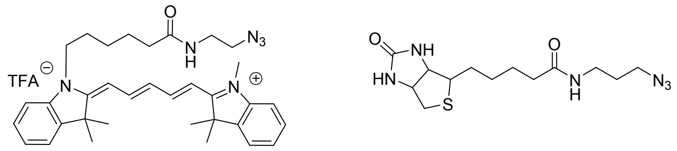

Cy5-N$_3$ (**31**) biotin-N$_3$ (**32**)

Figure S2. Structures of Cy5-N$_3$ (**31**) and biotin-N$_3$ (**32**).

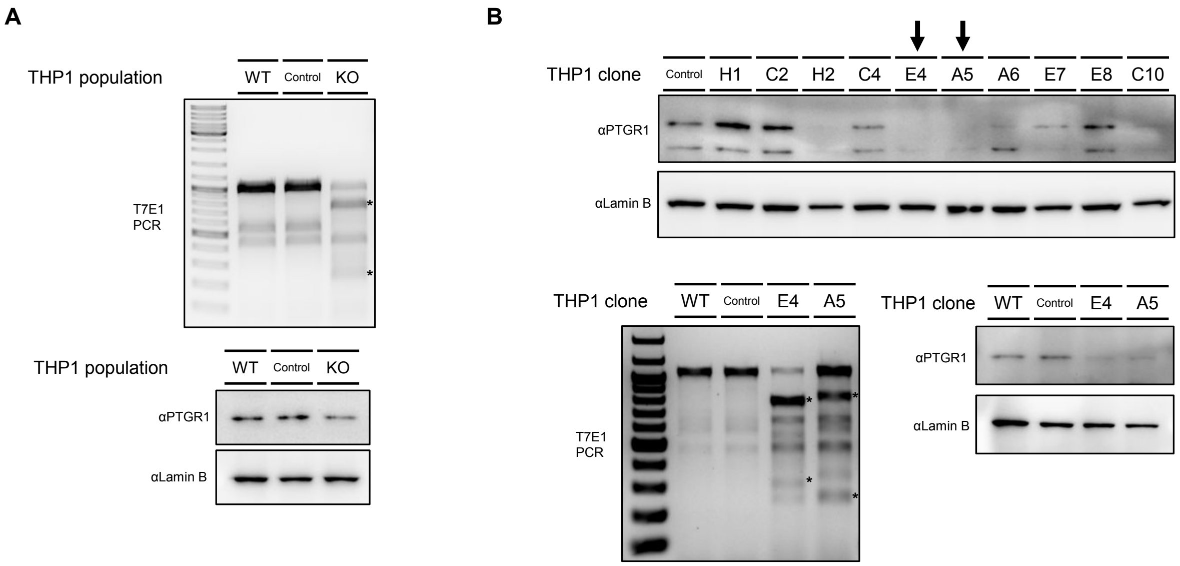

Figure S3. Characterization of CRISPR/Cas9-mediated PTGR1 knockout cells. **(A)** CRISPR/Cas9 knockout of THP1 using lentiviral infection resulted in incomplete knockdown of the protein, as judged by Western blot and T7E1 assay. **(B)** Single-cell colonies were grown and characterized by Western blot and T7E1 assay, resulting in two PTGR1 KO clones.

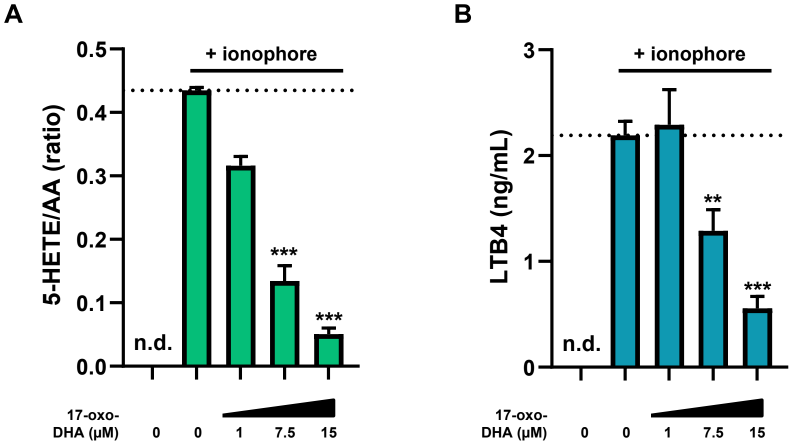

Figure S4. 17-oxo-DHA acts as anti-inflammatory lipid in human neutrophils. **(A)** 5-HETE/AA ratio and **(B)** LTB4 levels (LC-MS/MS) produced by ionophore-stimulated neutrophils. Cells were either pretreated for 10 min with 17-oxo-DHA or vehicle control. Data represent means ± SD of cells obtained from a representative donor **(**n = 3). *** $p < 0.001$ in comparison to control (dotted line) using a one-way ANOVA with Dunnett's multiple comparisons correction. n.d.; not detected.

A

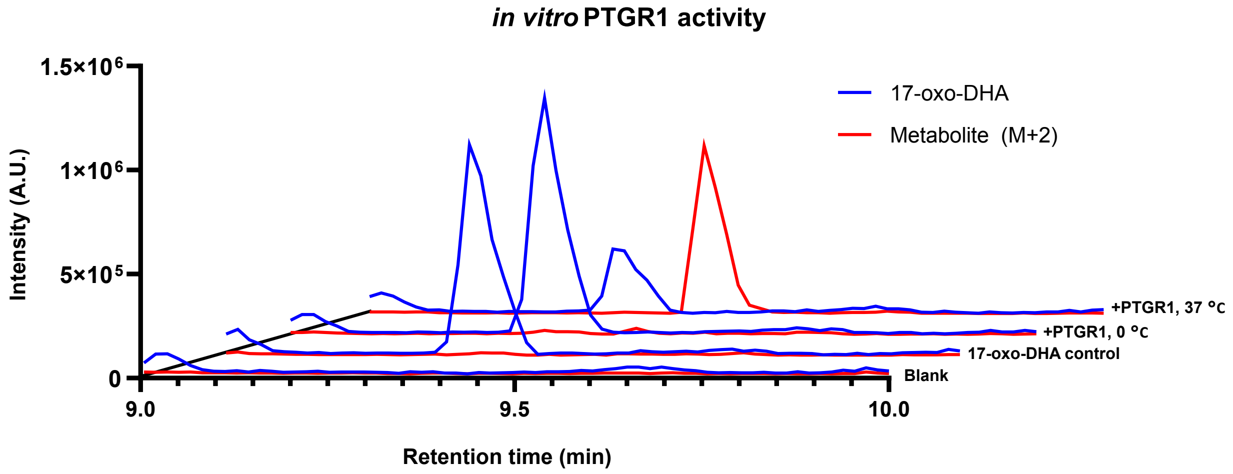

B

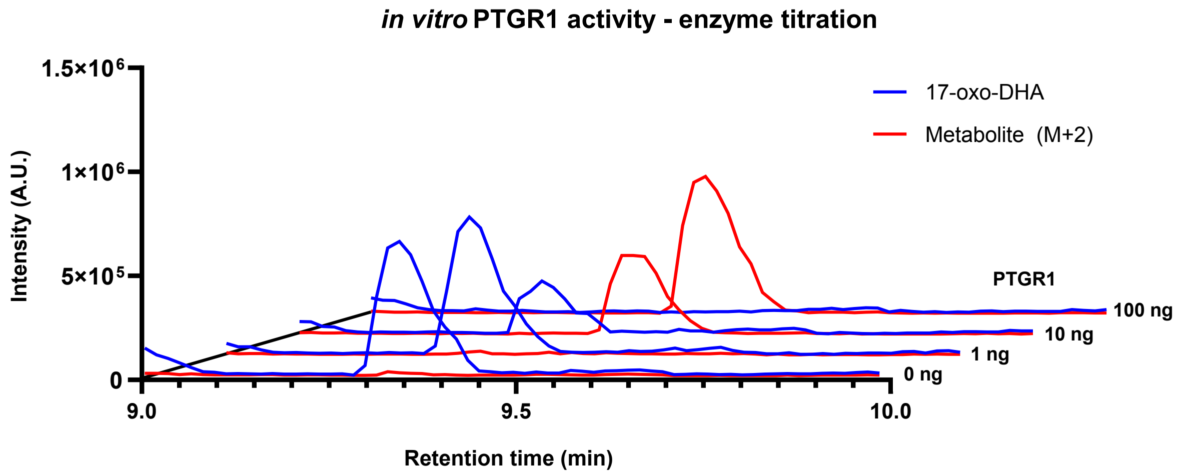

Figure S5. Recombinant PTGR1 reduces substrate 17-oxo-DHA to a metabolite with M+2. **(A)** Mass traces of 17-oxo-DHA (m/z = 341.211, blue) and reduced metabolite (m/z = 343.227, red). No lipid (blank) was compared to 1 µM 17-oxo-DHA (1 µM, control), and 17-oxo-DHA treated with 10 ng human recombinant PTGR1 at 0 ºC or 37 ºC **(B)** Titration of human recombinant PTGR1 with 17-oxo-DHA (1 µM). Full conversion is achieved with 100 ng PTGR1.

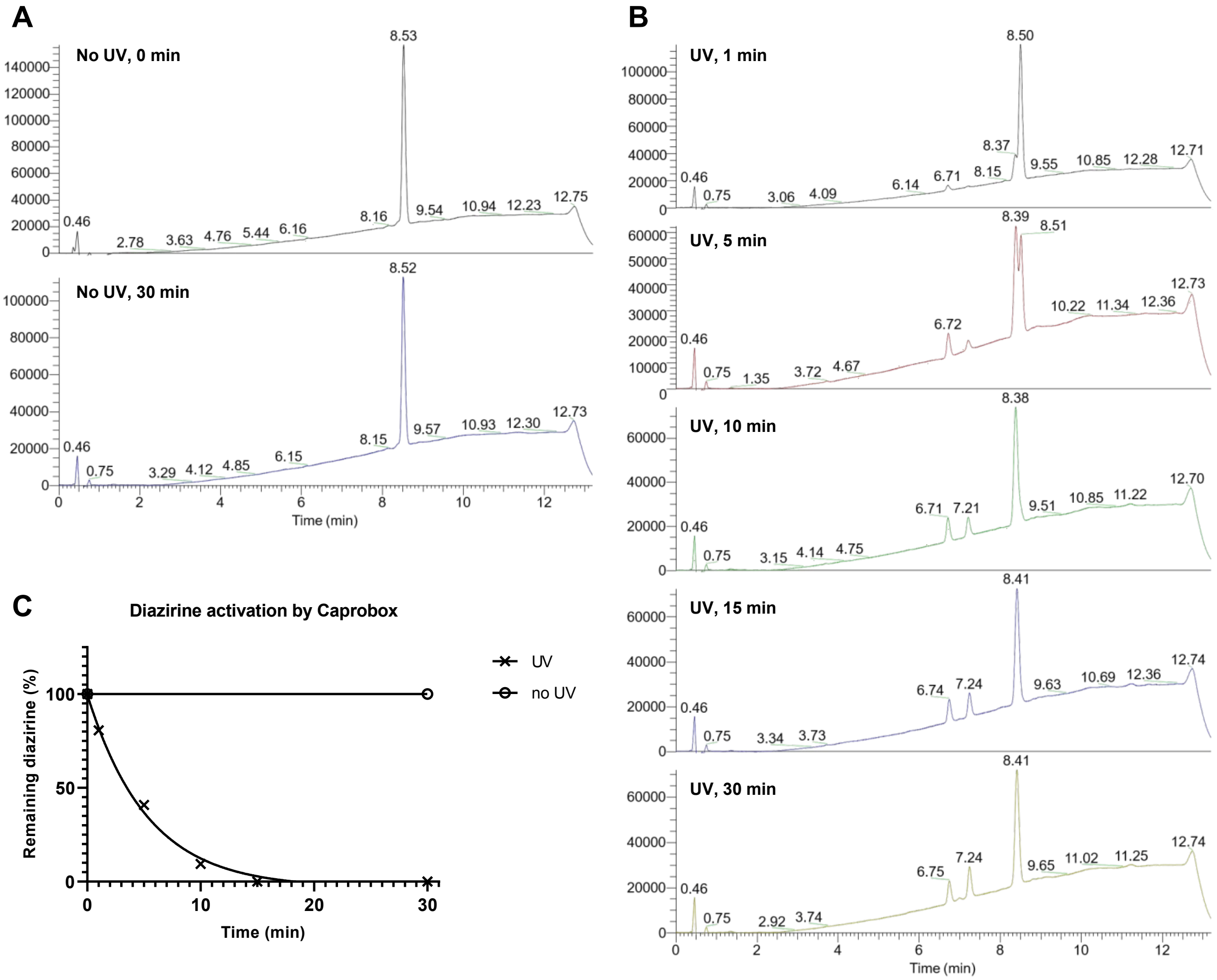

Figure S6. Characterization of diazirine activation by Caprobox. The probe was exposed to either **(A)** ambient light or **(B)** UV irradiation by the Caprobox at 350 nm. **(C)** Integrated remaining diazirine indicates near complete conversion after 10 minutes of UV exposure.

Supplemental methods

General remarks
Lipids were purchased from Cayman Chemicals and stored as ethanolic stocks under argon at -80 °C. Reagents and inhibitors used for biochemical experiments were purchased from Cayman Chemicals or Sigma Aldrich unless otherwise specified and stored at -20 °C, except Cy5-N_3 (Figure S3), which was synthesized according to previously published procedures. Biotin-N_3 (Figure S3) was purchased from Bio-Connect Life Sciences.

Cloning
DNA oligos were purchased at Sigma Aldrich or Integrated DNA Technologies. Cloning reagents were from Thermo Fisher. Full-length cDNA encoding human PTGR1 or GFP was obtained from Source Bioscience. Expression constructs were generated by PCR amplification and restriction/ligation cloning into a pcDNA3.1 vector, in frame with a C-terminal FLAG tag. All plasmids were isolated from transformed XL10-Gold or DH10B competent cells (prepared using E. coli transformation buffer set, Zymo Research) using plasmid isolation kits following the supplier's protocol (Qiagen). All sequences were verified by Sanger sequencing (Macrogen).

Cell culture
HEK-293-T cells were cultured at 37 °C under 7% CO_2 in DMEM (D6546, Merck) containing phenol red, stable glutamine, 10% (v/v) New Born Calf Serum (Thermo Fisher) and penicillin and streptomycin (200 µg/mL each, Duchefa). THP-1 cells were cultured at 37 °C under 7% CO_2 in RPMI 1640 (R5886, Merck) containing phenol red, 1 mM sodium pyruvate, stable glutamine, 10% (v/v) Fetal Calf Serum (Thermo Fisher) and penicillin and streptomycin (200 µg/mL each, Duchefa). Cells were passaged twice a week at 80-90% confluence by resuspension in fresh medium. Cell lines were purchased from ATCC and were tested regularly for mycoplasma contamination. Cultures were discarded after 2-3 months of use.

Transfection of HEK-293-T cells
ALDH1A1-, GFP- or PTGR1-overexpressing HEK-293-T cells were generated by seeding HEK-293-T cells on 12-well plates (4.0x10^4 cells/cm^2) 24 h before transfection. Culture medium was then aspirated and replaced with 400 µL fresh medium. A 3:1 (m/m) mixture of polyethylenimine (PEI) (1.875 µg/well) and plasmid DNA (0.625 µg/well) was prepared in serum-free culture medium (100 µL) and incubated for 15 min at rt. Transfection was performed by dropwise addition of the PEI/DNA mix to the cells. After 24 h, medium was refreshed. Cells were used 48 h post-transfection.

CRISPR/Cas9 KO generation

Guide design & constructs
sgRNA was selected for exon 2 of the *PTGR1* gene, with high efficiency and specificity as predicted by CHOPCHOP v2 online web tool.[2] The guide, along with a sgRNA targeting GFP as non-target control, was cloned into pLentiCRISPR-E vector (a gift from Phillip Abbosh, Addgene plasmid #78852) using the Esp3I restriction site, as previously described.[3,4] Primers are annotated in Table S1.

Lentiviral production
To produce lentiviral particles, HEK-293-T cells were seeded on 10 cm dishes 24 h prior to transfection. Culture medium (DMEM) was then aspirated and replaced with 5 mL fresh medium. A 3:1 (m/m) mixture of PEI (30 µg/dish) and plasmid DNA (10 µg/dish, 1:1:2:4 ratio (m/m/m/m) of pMD2.G, pRSV-Rev, pMDLg/pRRE, and pLentiCRISPR-E encoding Cas9 with sgRNA targeting *PTGR1* or non-targeting control sgRNA) was prepared in serum-free culture medium (1 mL) and incubated for 15 min at rt. Transfection was performed by dropwise addition of the PEI/DNA mix to the cells. After 6 h, medium was replaced with fresh medium (10 mL). After 48 h, the supernatant was collected in a 15 mL tube, supplemented with HEPES pH 7.4 (20 mM final), spun down (10,000 *g*, 5 min) and filtered over a 0.45 µm sterile filter and used immediately.

THP1 lentiviral transduction

5.0x10^{6} THP-1 cells in 5 mL medium (RPMI 1640) were supplemented with 5 mL medium containing lentiviral particles as aforementioned and incubated for 24 h. Cells were spun down (300 *g*, 5 min), supernatant was discarded and cells were resuspended in fresh medium containing 2 µg/mL puromycin (10-2100, Focus Biomolecules). Cells were passaged in medium containing 2 µg/mL puromycin for 7 days, after which cells were collected to assess knockout by T7E1 assay and western blot (Figure S3). To obtain single cell-derived knockout clones, cells were plated on 96-well plates at a density of 1 cell/well in a 1:1 mixture of fresh culture medium and THP-1-preconditioned medium. After 35 days, clones were selected by T7E1 assay and expanded. Further selection was done by western blot, of which two clones were picked (Figure S3).

T7E1 assay and aPTGR1 western blot

Genomic DNA was obtained by spinning down 5x10^{4} cells (2,000 *g*, 10 min), aspirating the medium and adding 50 µL QuickExtract™ (Epicentre). The samples were incubated at 65 °C for 6 min, mixed by vortexing and incubated at 98 °C for 2 min. Genomic DNA extracts were diluted in sterile water and directly used in PCR reactions. Genomic PCR reactions were performed on 5 µL isolated genomic DNA extract using Phusion High-Fidelity DNA Polymerase (Thermo Fisher) in Phusion GC buffer Green (Thermo Fisher) in a final volume of 45 µL, primers are annotated in Table S1.

For the T7E1 assay, genomic PCR products (20 µL) were denatured and reannealed in a thermocycler using the following program: 5 min at 95 °C, 95 to 85 °C using a ramp rate of -2 °C/s, 85 °C to 25 °C using a ramp rate of -0.2 °C/s. Annealed PCR product (8.5 µL) was mixed with NEB2 buffer (1 µL) and T7 endonuclease I (5 U, 0.5 µL; New England Biolabs), followed by a 30 min incubation at 37 °C. Digested PCR products were analyzed using agarose gel electrophoresis with GelRed staining. Agarose gels were analyzed using Image Lab 6.0.1 (BioRad).

To detect PTGR1 and Lamin B protein, THP1 lysate was resolved by SDS-PAGE (10% acrylamide gel, ±80 min, 180 V) along with protein marker (PageRuler™ Plus, Thermo Fisher). Proteins were then transferred to a 0.2 µm polyvinylidene difluoride membrane by Trans-Blot Turbo™ Transfer system (Bio-Rad). Membranes were washed with TBS (50 mM Tris pH 7.5, 150 mM NaCl) and blocked with 5% (w/v) milk in TBS-T (50 mM Tris pH 7.5, 150 mM NaCl, 0.05% (w/v) Tween-20) for 1 h at rt. Membranes were then incubated with primary antibody rabbit-anti-PTGR1 (PA5101698, Thermo Fisher, 1:1,000 in 5% (w/v) BSA in TBS-T, overnight, 4 °C) or rabbit-anti-Lamin B (PA5-19468, Thermo Fisher, 1:4,000 in 5% (w/v) BSA in TBS-T, overnight, 4 °C), washed three times with TBS-T, incubated with secondary mouse-anti-rabbit-HRP (sc-2357-CM, Santa Cruz Biotechnologies, 1:5,000 in 5% (w/v) milk in TBS-T, 1 h, rt) and washed three times with TBS-T and once with TBS. Western blots were developed using Clarity Western ECL Substrate (Bio-Rad) and chemiluminescence was detected on ChemiDoc™ MP (Bio-Rad) in the chemiluminescence channel.

Table S1. sgRNA oligos (top, bottom), sgRNA targets (in bold), and T7E1 primers (forward, reverse).

sgRNA Target	Construct	Oligo Sequences
***PTGR1* – Exon 2**	#	sgRNA top: CACCG**TCACCGTGGATCCCTACATG**<br>sgRNA bottom: AAAC**CATGTAGGGATCCACGGTGA**C<br>Forward primer: CTGAGGTTTGGTGTACGAATGA<br>Reverse primer: TTGAGTCAACCACAGATTCACC
**Non-targeting control**	#	sgRNA top: CACC**GCACTACCAGAGCTAACTCA**<br>sgRNA bottom: AAAC**TGAGTTAGCTCTGGTAGTGC**

Gel-based AfBPP of HEK-293-T cells

HEK-293-T cells in log phase on 12-well plates were treated as follows: Growth medium was aspirated, the cells were washed with PBS (0.5 mL) and a solution of indicated competitor lipid was added in serum-free DMEM supplemented with 0.1% delipidated BSA (0.5 mL) for 30 min at 37 °C. Whenever higher concentrations than 10 µM were used, the ethanolic stock was concentrated by speedvac and the lipid resuspended in treatment medium by sonication (Branson Sonifier probe sonicator, 10s, 30% amplitude). Then, pac-DHA (**4**) or pac-17-HDHA (**5**) (10 µM from 10 mM ethanolic stock) in serum-free DMEM supplemented with 0.1% delipidated BSA (0.5 mL) was added in serum-free DMEM supplemented with 0.1% delipidated BSA (0.5 mL) for 30 min at 37 °C. The cells were incubated for 30 min at 37 °C after which the medium was aspirated and the cells were irradiated ("UV") in 1 mL ice-cold PBS using a Caprobox™ (10 min, 4 °C, 350 nm, Figure S6) or exposed to ambient light ("No UV"). The cells were harvested by pipetting and pelleted by centrifugation (1,000 *g*, 10 min, 4 °C). The supernatant was removed and the cells were lysed by resuspension in lysis buffer (250 mM sucrose, 20 mM HEPES pH 7.5, 1 mM $MgCl_2$) and sonication in a bath sonicator (0 °C, 10 s). Protein concentration was measured by Qubit™ assay (Invitrogen) and the samples were adjusted to 1.5 mg/mL and a volume of 100 µL, after which the samples were treated with 10.95 µL click mix (5.5 µL aq. 25 mM $CuSO_4$, 3.25 µL aq. 250 mM NaAsc, 1.1 µL 25 mM THPTA in DMSO, 1.1 µL 0.9 mM Cy5-N_3 in DMSO) and left at rt for 1 h. Samples were then quenched by addition of 4X Laemmli buffer, boiled (5 min, 95 °C) and resolved by SDS-PAGE (10% acrylamide gel, ±80 min, 180 V) along with protein marker (PageRuler™ Plus, Thermo Fisher). In-gel fluorescence was measured in the Cy3- and Cy5-channel (Chemidoc™ MP, Bio-Rad).

Proteins were then transferred to a 0.2 µm polyvinylidene difluoride membrane by Trans-Blot Turbo™ Transfer system (Bio-Rad). Membranes were washed with TBS (50 mM Tris pH 7.5, 150 mM NaCl) and blocked with 5% (w/v) milk in TBS-T (50 mM Tris pH 7.5, 150 mM NaCl, 0.05% (w/v) Tween-20) for 1 h at rt. Membranes were then incubated with primary antibody mouse-anti-FLAG (F3156, Merck, 1:2,000 in 5% (w/v) BSA in TBS-T, 1 h, rt), washed three times with TBS-T, incubated with secondary goat-anti-mouse-HRP (sc-2005, Santa Cruz Biotechnologies, 1:5,000 in 5% (w/v) BSA in TBS-T, 1 h, rt) and washed three times with TBS-T and once with TBS. Luminol development solution (10 mL of 1.4 mM luminol in 100 mM Tris pH 8.8 + 100 µL of 6.7 mM *p*-coumaric acid in DMSO + 3 µL of 30% (v/v) H_2O_2) was added and chemiluminescence was detected on ChemiDoc™ MP (Bio-Rad) in the chemiluminescence channel and colorimetric channel for the protein marker.

Macrophage and neutrophil isolation and treatment

This study was approved by the local medical ethical committee of the LUMC (METC), and written informed consent was given by all donors. Neutrophils were isolated from fresh 50 mL EDTA blood containers via DextranT500 sedimentation (Pharmacosmos). The upper layer was collected, followed by Ficoll density gradient separation. The remainder of the erythrocytes was removed by hypotonic lysis. Purity was checked by FACS (LSRIII, BD Biosciences) by staining the cells with CD3-AF700 (clone UCHT1)/CD15-APC (clone HI98)/CD16-PE (clone 3G8)/CD19-FITC (clone HIB19) and was typically above 97%. Isolated neutrophils were resuspended in Dulbecco's phosphate-buffered saline (DPBS) with $MgCl_2$ and $CaCl_2$ (D8662, Merck). Human peripheral blood

mononuclear cells (PBMCs) were isolated by Ficoll density gradient from healthy donor buffy coats (Sanquin). Blood monocytes were isolated by positive selection from PBMCs using MACS CD14 Microbeads (Miltenyi Biotec) and purity was checked by FACS (LSRIII, BD Biosciences), by staining the cells with CD14-PE (clone MφP9). Purity was typical above >99%. Monocytes were differentiated for seven days in RPMI 1640 medium (Gibco) containing 8% FCS, 100 U/mL penicillin and streptomycin, 2 mM Glutamax (Thermo Fisher) and 50 ng/mL M-CSF (R&D Systems). 1/3 of the medium was replenished containing 150 ng/mL M-CSF on day three and five. Phenotype was checked before experiments by visual inspection by assessing the typical morphology of M-CSF-treated monocyte-derived macrophages (elongated and spindle-like). Moreover, phenotype was also confirmed by performing IL-12 OptEIA (BD Biosciences), IL-10 PeliPair reagent set (Sanquin), and TNFα OptEIA (BD Biosciences) ELISA on supernatant of cells stimulated for 24 h with 10 ng/mL LPS (Merck). M-CSF-treated monocyte-derived macrophages secreted low IL-12 and TNFα and high IL-10 levels.

M-CSF-treated monocyte-derived macrophages (M2 macrophages) were harvested using Accutase (Merck) and for lipidomics experiments 2.5×10^5 cells were seeded in 24-well plates in 250 µL medium. All experiments were performed in phenol red-free RPMI 1640 medium (Gibco), supplemented with 0.1% (w/v) delipidated BSA (Merck), 100 U/mL penicillin and streptomycin, and 2 mM Glutamax. For lipidomics experiments, cells were pretreated with indicated amounts of 17-(S)-HDHA (Cayman Chemicals) or vehicle, HPLC grade ethanol (Fischer Scientific), and indicated amount of inhibitor (Merck) or vehicle, 0.02% HPLC grade DMSO. Stimulation of both macrophages and neutrophils was done using 4 µM calcium ionophore A23178 (Merck) for 10 min.

THP1 treatment for lipidomics

PTGR1 KO or control (GFP sgRNA) THP1 cells were counted, spun down (300 g, 5 min) washed with DBPS and 1.0×10^6 cells were plated on 12-well plates in 500 µL phenol red-free RPMI 1640 medium (Merck), supplemented with 0.1% (w/v) delipidated BSA (Merck), 200 µg/mL penicillin and streptomycin, and 2 mM Glutamax. Cells were treated with indicated amount of inhibitor (Merck) or vehicle, 0.01% HPLC grade DMSO, followed by indicated amounts of 17-(S)-HDHA (Cayman Chemicals) or vehicle, 0.01% HPLC grade ethanol (Fischer Scientific).

Lipid isolation and LC-MS/MS

After treatment, cells and supernatant were quenched using three volumes of MeOH (Honeywell, 349661L) and internal standard mix containing known concentrations of three internal standards: 5 ng/mL DHA-d5, 500 pg/mL LTB4-d4 and 500 pg/mL 15S-HETE-d8 (Cayman Chemicals) for subsequent quantification. All samples were stored at -80 °C under argon until analysis. Quenched samples were centrifuged (20,000 *g*, 5 min) and the supernatant was transferred into an autosampler vial containing an equal volume of H_2O (Honeywell) before LC-MS/MS analysis, which was carried out as previously published.[5] The MS method was slightly adapted: it was extended with a MRM for 17-oxo-DHA (341 / 111). Lipid measurements were performed using a QTrap 6500 mass spectrometer in negative ESI mode (Sciex), coupled to a LC system employing LC-30AD pumps, a SIL-30AC auto sampler, and a CTO-20AC column oven (Shimadzu). A Kinetex C18 50 × 2.1 mm, 1.7 µm column combined with a C8 precolumn (Phenomenex) was used and kept at 50 °C. A gradient of water and methanol with 0.01% acetic acid was used. The injection volume was 40 µL and a flow rate of 400 µL/min was used. MRM transitions used to identify LM were based on previous work by the group of M. Giera.[5] Peaks were integrated with manual supervision and retention time corrected to corresponding IS (RRT) with MultiQuant™ 2.1 (Sciex). Only peaks with a signal to noise (S/N) >10 were quantified. Calibration curves were constructed using authentic synthetic standards 17-HDHA, 17-oxo-DHA, AA, DHA, LTB4, 5-HETE, LTD4, LTE4, 15S-HETE-d8, LTB4-d4 and DHA-d5 which were purchased from Cayman Chemicals.

In vitro LC-MS-based 17-oxo-DHA conversion assay
Recombinant PTGR1 protein (GTX68309-pro, GeneTex) was diluted to 0.4 ng/µL in assay buffer (100 mM sodium phosphate pH 7.0). The substrate 17-oxo-DHA (9000346, Cayman Chemicals, 1 mM stock in MeOH) was prediluted in assay buffer supplemented with 0.5 mM NAPDH. The enzyme reaction was initiated by addition of substrate mix (25 µL) to the protein (25 µL) in low-binding Eppendorf tubes, followed by incubation at 37 °C or on ice for 1 h. Final reaction conditions: 10 ng PTGR1 (or indicated), 1 µM 17-oxo-DHA, 0.25 mM NAPDH. Reactions were then quenched by addition of ice-cold MeOH (Honeywell, 349661L, 150 µL), followed by centrifugation (15,000 *g*, 1 min, 4 °C) to remove precipitated protein, after which the supernatant was transferred to LC-MS vials and stored under nitrogen atmosphere until measurement. Samples without protein or substrate were included as negative controls. Samples were analyzed as per the previous section and the MS intensities for 17 oxo-DHA (m/z = 341.211) and its reduced metabolite (m/z = 343.227) were plotted as x/y coordinates.

AfBPP of M2 macrophages
M-CSF-treated monocyte-derived macrophages were plated on 6-well plates. The following day, they were washed with PBS (1 mL) and probe incubation was started by adding pac-DHA or pac-17-HDHA (10 µM from 10 mM ethanolic stock) in serum-free RPMI supplemented with 0.1% (w/v) delipidated BSA (1 mL). The cells were incubated for 30 min at 37 °C after which the medium was aspirated and replaced with ice-cold PBS. The cells were irradiated using a Caprobox™ (10 min, 4 °C, 350 nm, "UV") or exposed to ambient light (10 min, 4 °C, "No UV"). The PBS was collected in tubes and floating cells were spun down (1,000 *g*, 10 min, 4 °C) and the PBS was aspirated. The cells in the wells were lysed with lysis buffer (250 µL, 250 mM sucrose, 1X protease inhibitor cocktail (Roche), 20 mM HEPES pH 7.5, 1 mM $MgCl_2$). The material was harvested by scraping and combined with the cell pellet. This was sonicated (Branson Sonifier probe sonicator, 10 x 1 s pulses, 10% amplitude). Protein concentration was measured by Qubit™ assay (Invitrogen) and the samples were adjusted to 0.5 mg/mL and a volume of 440 µL, of which 40 µL was reserved for gel analysis. For gel analysis, the 40 µL lysate was treated with freshly prepared click mix (4.37 µL per sample: 2.19 µL aq. 25 mM $CuSO_4$, 1.3 µL aq. 250 mM NaAsc, 0.44 µL 25 mM THPTA in DMSO, 0.44 µL 0.9 mM Cy5-N_3 in DMSO) and left at rt for 1 h in the dark. Samples were then quenched by addition of 4X Laemmli buffer, boiled (5 min, 95 °C) and resolved by SDS-PAGE (10% acrylamide gel, ±80 min, 180 V) along with protein marker (PageRuler™ Plus, Thermo Fisher). In-gel fluorescence was measured in the Cy3- and Cy5-channel (Chemidoc™ MP, Bio-Rad) and gels were stained with Coomassie after scanning.

Mass spectrometric analysis of tryptic peptides, identification and quantification
The pulldown experiment is performed as earlier described, with minor adjustments.[6,7] The lysates (400 µL) were subjected to a click reaction with freshly prepared click mix (43.7 µL per sample: 21.9 µL aq. 25 mM $CuSO_4$, 13 µL aq. 250 mM NaAsc, 4.4 µL 25 mM THPTA in DMSO, 4.4 µL 2.25 mM biotin-N_3 in DMSO) at rt for 1 h. Proteins were precipitated by addition of HEPES buffer (50 µL, 50 mM, pH 7.5), MeOH (666 µL), $CHCl_3$ (166 µL) and MilliQ (150 µL), vortexing after each addition. After spinning down (1,500 *g*, 10 min) the upper and lower layer were aspirated and the protein pellet was resuspended in MeOH (600 µL) by sonication (Branson Sonifier probe sonicator, 10 x 0.5 s pulses, 10% amplitude). The proteins were spun down (20,000 *g*, 5 min) and the MeOH was aspirated. The proteins were then redissolved in 6 M urea (500 µL) with 25 mM NH_4HCO_3 for 15 min, followed by reduction (65 °C, 15 min, 800 rpm shaking) with DTT (5 µL, 1 M). The samples were allowed to reach rt and proteins were alkylated (30 min) with IAA (40 µL, 0.5 M) in the dark. 140 µL SDS (10% w/v) was added and the samples were spun down (1,000 *g*, 5 min). They were transferred to 5 mL PBS containing 50 µL avidin agarose resin (Pierce, 100 µL of a 50% slurry, prewashed twice with 6 mL PBS + 0.5% SDS and once with 6 mL PBS) and incubated for 2 h while rotating. The beads were then spun down (2,000 *g*, 2 min) and washed (3 x PBS + 0.25% SDS, 2 x PBS, 1 x MilliQ). The beads were resuspended in digestion buffer (250 µL, 100 mM Tris pH 7.8, 100 mM NaCl, 1 mM $CaCl_2$, 2% (v/v) acetonitrile, sequencing grade trypsin (Promega, 0.25 µg)) and transferred to low-binding tubes (Sarstedt) and incubated while shaking overnight (16 h, 37 °C, 1,000 rpm). Trypsin was quenched with 12.5 µL formic acid (LC-MS grade) and the beads were then filtered off over a Bio-Spin column (BioRad, 400 *g*, 5 min), collecting the flow-through in a new 2 mL tube. Samples were added on C18 stagetips[8] (preconditioned

with 50 µL MeOH, then 50 µL of 0.5% (v/v) formic acid in 80% (v/v) acetonitrile/MilliQ (solution B) and then 50 µL 0.5% (v/v) formic acid in MilliQ (solution A) by centrifugation (600 *g*, 2 min)). The peptides were washed with solution A (100 µL, 800 *g*, 3 min) and eluted into new low-binding tubes using solution B (100 µL, 800 *g*, 3 min). Samples were concentrated using an Eppendorf speedvac (Eppendorf Concentrator Plus 5301) and redissolved in LC-MS solution (30 µL per sample: 28.5 µL MilliQ, 2.85 µL acetonitrile, 0.095 µL formic acid, 600 fmol yeast enolase peptide digest (Waters, 186002325)).

Samples were measured using a NanoACQUITY UPLC System coupled to a SYNAPT G2-Si high definition mass spectrometer (Waters). The peptides were separated using an analytical column (HSS-T3 C18 1.8 µm, 75 µm x 250 mm, Waters) with a concave gradient (5 to 40% acetonitrile in H_2O with 0.1% formic acid). [Glu^1]-fibrinopeptide B was used as lock mass. Mass spectra were acquired using the $UDMS^e$ method. The mass range was set from 50 to 2,000 Da with a scan time of 0.6 seconds in positive, resolution mode. The collision energy was set to 4 V in the trap cell for low-energy MS mode. For the elevated energy scan, the transfer cell collision energy was ramped using drift-time-specific collision energies. The lock mass is sampled every 30 seconds. For raw data processing, Progenesis QI for proteomics was used with the following parameters to search the human proteome from Uniprot (Table S2). Proteins were quantified using Label-Free Quantification (LFQ) and probe 5-enriched targets were defined as proteins with an LFQ ratio of >2 between '+ UV' and 'no UV' samples, as well as a p-value <0.05 after performing a Student's t-test. Albumin was not included in the analysis of probe targets.

Table S2. Parameters used for Progenesis QI.

Parameter	Value
**Lock mass *m/z* value**	785.8426
**Low energy threshold**	150 counts
**Elevated energy threshold**	30 counts
**Digest reagent**	Trypsin
**Missed cleavages**	Max 2
**Modifications**	Fixed carbamidomethyl C, variable oxidation M
**FDR less than**	1%
**Minimum fragments/peptide**	2
**Minimum fragments/protein**	5
**Minimum peptides/protein**	1
**Minimum peptide score for quantification**	5.5
**Identified ion charges for quantification**	$2/3/4/5/6/7^+$

Statistical analysis

Unless otherwise noted, all data represent means ± SD. Statistical significance was determined using Student's t-tests (two-tailed, unpaired) or a ANOVA with Dunnett's or Tukey's multiple comparisons correction. *** p <0.001; ** p <0.01; * p <0.05; n.s. if p >0.05. All statistical analysis was conducted using Graphpad Prism 8.1.1 or Microsoft Excel.

Synthesis

General remarks

Dry solvents were prepared by storage on activated 4 Å molecular sieves for at least 24 hours. The reactions were performed under an inert atmosphere of nitrogen gas unless stated otherwise. All reagents were purchased from Alfa Aesar, Sigma Aldrich/Merck or Acros and used without further purification. Flash column chromatography was performed using SiliCycle silica gel type SiliaFlash P60 (230-400 mesh). TLC analysis was performed on Merck silica gel 60/Kieselguhr F254, 0.25 mm. Compounds were visualized using $KMnO_4$ stain (K_2CO_3 (40 g), $KMnO_4$ (6 g), and water (600 mL)). ^1H NMR and ^{13}C NMR spectra were recorded on a Bruker AV-400 (400 MHz) or AV-500 (500 MHz) spectrometer. Chemical shift values are reported in ppm relative to the tetramethylsilane signal for ^1H NMR (δ = 0 ppm) and relative to the solvent signal of $CDCl_3$ for ^{13}C NMR (δ = 77.16 ppm). Data are reported as follows: Chemical shifts (δ), multiplicity (s = singlet, d = doublet, dd = doublet of doublets, ddt = doublet of doublet of triplets, td = triplet of doublets, t = triplet, q = quartet, p = pentet, bs = broad singlet, m = multiplet), coupling constants J (Hz), and integration. High resolution mass spectra (HRMS) were recorded by direct injection on a q-TOF mass spectrometer (Synapt G2-SI) equipped with an electrospray ion source in positive mode with Leu-enkephalin (m/z = 556.2771) as an internal lock mass. The instrument was calibrated prior to measurement using the MS/MS spectrum of [Glu^1]-fibrinopeptide B.

Preparative HPLC separations were performed with an Agilent Technologies 1200 series HPLC system using a Gemini column (5 µm C18, pore size: 100 Å, 250 x 10.0 mm) or Nucleodur column (5 µm C18, pore size: 110 Å, 250 x 10.0 mm) using a specified linear gradient (Gradient of solvent B in solvent A in 12 min, flow rate of 5 mL/min, detection at 210 - 600 nm by a diode array and Agilent 6130 series quadrupole mass detector, solvent A: 0.2% (v/v) TFA in H_2O, solvent B: acetonitrile). Analytical LC-MS was performed using a C18 column (50 x 4.6 mm, 3 µm; Nucleodur Gravity, Macherey-Nagel) connected to a Vanquish UHPLC system (Thermo Scientific) with a Vanquish Diode Array detector (Thermo Scientific) coupled to a LCQ™ Fleet (Thermo Scientific) via electrospray ionization (ESI). Acetonitrile and water containing TFA (0.1%) were used for chromatographic separation using an indicated gradient.

Fragmentation of pac-17-HDHA (**5**) was done after reduction of the double bonds, as LM-MS/MS fragmentation in negative mode was not successful, possibly due to activation of the diazirine during tandem MS. Pac-17-HDHA (**5**) was analyzed by complete hydrogenation of an analytical sample (50 nmol) in EtOH (1 mL) using catalytic PtO_2 and hydrogen gas, followed by the filtration of the catalyst by cotton, removal of the solvent under a stream of N_2 gas and redissolving in 100 µL acetonitrile, injecting 10 µL on a C18 column (50 x 4.6 mm, 3 µm; Gemini) connected to a Agilent Technologies 1260 Infinity system and eluting it with acetonitrile/H_2O (70:30) with 10 mM NH_4OAc onto a 6120 Quadrupole LC/MS (Agilent Technologies) equipped with an electrospray ion source in positive mode (source voltage 4 kV, sheath gas flow 10, capillary temperature 623 K).

Ethyl 3-oxohept-6-ynoate (28)

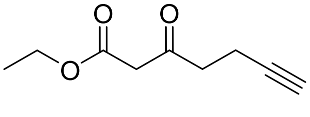

Freshly distilled diisopropylamine (20.43 mL, 143 mmol) was dissolved in dry THF (60 mL) and cooled to -78 °C. *n*-BuLi (1.4 M in hexane, 102 mL, 143 mmol) was added dropwise and the reaction was stirred for 15 min. The mixture was allowed to reach -40 °C after which ethyl 3-oxobutanoate (8.23 mL, 65.2 mmol) in dry THF (40 mL) was added dropwise. After 30 min of stirring, propargyl bromide (80 wt.% in toluene, 7.02 mL, 65.2 mmol) was added dropwise after which the reaction was allowed to reach 0 °C. After 1.5 h of stirring, it was quenched with 0.5 M aq. HCl (200 mL) and diluted with Et_2O (200 mL). The organic layer was collected and the aq. layer extracted with Et_2O (200 mL). The combined organic layers were washed with brine (100 mL), dried over $MgSO_4$, filtered and concentrated under reduced pressure to afford a dark brown oil, which was distilled (105-110 °C, 6.8 mmHg) to afford the title compound as a clear oil (4.943 g, 29.4 mmol, 45%). R_f = 0.60 (EtOAc/pentane = 1:4); ^{1}H NMR (400 MHz, $CDCl_3$) δ 4.20 (q, *J* = 7.1 Hz, 2H), 3.48 (s, 2H), 2.82 (t, *J* = 7.2 Hz, 2H), 2.48 (td, *J* = 7.2, 2.7 Hz, 2H), 1.98 (t, *J* = 2.7 Hz, 1H), 1.29 (t, *J* = 7.1 Hz, 3H); ^{13}C NMR (101 MHz, $CDCl_3$) δ 200.63, 166.95, 82.59, 69.05, 61.51, 49.23, 41.63, 14.13, 12.84. Spectra were consistent with previously reported data.[9]

2-(2-(But-3-yn-1-yl)-1,3-dioxolan-2-yl)ethan-1-ol (29)

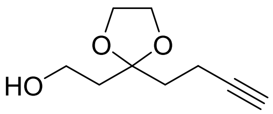

In a microwave vial, ketone **28** (0.8619 g, 5.12 mmol), ethylene glycol (2.143 mL, 38.4 mmol), *p*-TsOH·$2H_2O$ (0.049 g, 0.256 mmol) and triethyl orthoformate (2.56 mL, 15.37 mmol) were added, the vial was sealed and the reaction was stirred at 80 °C for 1.5 h. The mixture was diluted with Et_2O (5 mL) and quenched with sat. aq. $NaHCO_3$ (5 mL). It was then diluted with Et_2O (100 mL) and sat. aq. $NaHCO_3$ (100 mL). The layers were separated and the organic layer washed with brine (100 mL), dried over Na_2SO_4 and concentrated under reduced pressure to afford crude ethyl 2-(2-(but-3-yn-1-yl)-1,3-dioxolan-2-yl)acetate. The residue was diluted with dry THF (20 mL) and added dropwise to a cold (0 °C) mixture of $LiAlH_4$ (0.389 g, 10.24 mmol) and dry THF (20 mL). The reaction was allowed to reach rt and after 0.5 h it was cooled to 0 °C and quenched with EtOAc (5 mL). After 15 min, 10% (w/v) aq. Rochelle's salt (40 mL) was added and the mixture was allowed to reach rt overnight. The mixture was then diluted with brine (40 mL) and extracted with Et_2O (5 x 40 mL). The combined organic layers were washed with brine (100 mL), dried over Na_2SO_4, filtered, concentrated under reduced pressure and filtered over a plug of silica with EtOAc/pentane (1:1) to afford the title compound as a yellow oil (0.8204 g, 4.82 mmol, 94%). R_f = 0.63 (EtOAc); ^{1}H NMR (400 MHz, $CDCl_3$) δ 4.06 – 3.93 (m, 4H), 3.76 (q, *J* = 5.4 Hz, 2H), 2.72 (t, *J* = 5.5 Hz, 1H), 2.32 – 2.24 (m, 2H), 1.99 – 1.91 (m, 5H); ^{13}C NMR (101 MHz, $CDCl_3$) δ 111.12, 84.04, 68.36, 65.03, 58.76, 38.35, 35.99, 13.22. Spectra were consistent with previously reported data.[10]

1-Hydroxyhept-6-yn-3-one (30)

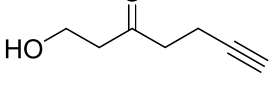

To a solution of ketal **29** (0.8204 g, 4.82 mmol) in acetone (19 mL) and water (1 mL) was added *p*-TsOH·$2H_2O$ (0.229 g, 1.205 mmol) and the reaction was stirred for 1 h at 50 °C. It was quenched with sat. aq. $NaHCO_3$ (50 mL) and diluted with EtOAc (50 mL). The aq. layer was separated and extracted with EtOAc (50 mL). The combined organic layers were washed with brine (50 mL), dried over $MgSO_4$, filtered, concentrated under reduced pressure and purified by column chromatography (EtOAc/pentane = 1:2 to 1:1) to afford the title compound as a yellow oil (0.565 g, 4.48 mmol, 93%). R_f = 0.62 (EtOAc); ^{1}H NMR (400 MHz, $CDCl_3$) δ 3.87 (t, *J* = 5.5 Hz, 2H), 2.76 – 2.68 (m, 5H), 2.46 (td, *J* = 7.1, 2.6 Hz, 2H), 1.98 (t, *J* = 2.7 Hz, 1H); ^{13}C NMR (101 MHz, $CDCl_3$) δ 209.03, 82.89, 68.95, 57.62, 44.68, 41.81, 12.78. Spectra were consistent with previously reported data.[10]

2-(3-(But-3-yn-1-yl)-3*H*-diazirin-3-yl)ethan-1-ol (31)

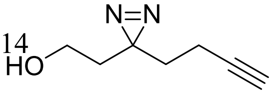

Ketone **30** (0.660 g, 5.23 mmol) was dried by coevaporation with toluene. It was dissolved

in dry MeOH (15 mL) and cooled to 0 °C. NH_3 gas was bubbled through the solution for 30 min under stirring after which the reaction was stirred for 5 h. Then, hydroxylamine-*o*-sulfonic acid (0.887 g, 7.85 mmol) in dry MeOH (5 mL) was added dropwise and the reaction was allowed to reach rt overnight. It was filtered over celite and the filtrate was concentrated under reduced pressure. The residue was redissolved in dry MeOH (10 mL) and Et_3N (1.094 mL, 7.85 mmol) was added. The reaction was cooled to 0 °C and a sat. solution of I_2 in dry MeOH was added dropwise until the color persisted for 20 min (8 mL). The reaction was diluted with EtOAc (100 mL) and quenched with 1 M aq. $Na_2S_2O_3$ (100 mL). The layers were separated, the aq. layer was extracted with EtOAc (100 mL) and the combined organic layers were washed with 1 M aq. HCl (100 mL) and brine (100 mL), dried over $MgSO_4$, filtered and concentrated under reduced pressure. Column chromatography of the residue (EtOAc/pentane = 1:6 to 1:3) afforded the title compound as a yellow oil (0.2677 g, 1.937 mmol, 37%). R_f = 0.59 (EtOAc/pentane = 1:1); ^{1}H NMR (400 MHz, $CDCl_3$) δ 3.49 (t, *J* = 6.2 Hz, 2H), 2.10 – 2.01 (m, 2H), 1.99 (s, 1H), 1.77 – 1.65 (m, 4H); ^{13}C NMR (101 MHz, $CDCl_3$) δ 82.95, 69.34, 57.36, 35.56, 32.68, 26.72, 13.31; HRMS: Calculated for $[C_7H_{10}N_2O+H]^+$ 139.0866, found 139.0865.

3-(But-3-yn-1-yl)-3-(2-iodoethyl)-3*H*-diazirine (10)

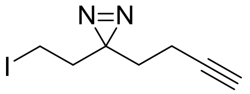

To a cooled (0 °C) solution of alcohol **31** (0.6617 g, 4.79 mmol) dry DCM (24 mL) was added imidazole (0.978 g, 14.37 mmol), I_2 (1.459 g, 5.75 mmol) and PPh_3 (1.382 g, 5.27 mmol). The reaction was allowed to reach rt and stirred for 3 h. It was cooled to 0 °C and quenched with 10% aq. $Na_2S_2O_3$ (50 mL) and diluted with DCM (100 mL). The aq. layer was isolated and extracted with DCM (100 mL) and the combined organic layers were dried over $MgSO_4$, filtered and concentrated under reduced pressure. Column chromatography of the residue (Et_2O/pentane = 1:49 to 1:16) afforded the title compound as a yellow liquid (0.8609 g, 3.47 mmol, 73%). R_f = 0.69 (EtOAc/pentane = 1:19); ^{1}H NMR (400 MHz, $CDCl_3$) δ 2.90 (t, *J* = 7.6 Hz, 2H), 2.13 (t, *J* = 7.6 Hz, 2H), 2.07 – 2.00 (m, 3H), 1.72 – 1.67 (m, 2H); ^{13}C NMR (101 MHz, $CDCl_3$) δ 82.50, 69.56, 37.54, 31.84, 28.67, 13.32, -3.76. Spectra were consistent with previously reported data.[11]

(2-(3-(But-3-yn-1-yl)-3*H*-diazirin-3-yl)ethyl)triphenylphosphonium iodide (6)

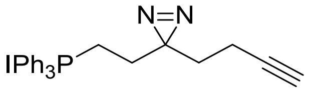

To a solution of iodide **10** (0.2621 g, 1.057 mmol) in dry acetonitrile (4.2 mL) in a microwave vial was added PPh_3 (1.386 g, 5.28 mmol) and the vial was sealed. The mixture was degassed and stirred at 70 °C overnight, concentrated under reduced pressure and purified by column chromatography (MeOH/DCM = 1:100 to 1:19) to afford the title compound as a white solid (0.539 g, 1.056 mmol, quant.). R_f = 0.49 (MeOH/DCM = 1:9); ^{1}H NMR (400 MHz, $CDCl_3$) δ 7.90 – 7.75 (m, 10H), 7.74 – 7.69 (m, 5H), 3.83 – 3.70 (m, 2H), 2.11 – 2.01 (m, 2H), 2.01 – 1.92 (m, 2H), 1.90 (t, *J* = 2.6 Hz, 1H), 1.85 – 1.72 (m, 2H); ^{13}C NMR (101 MHz, $CDCl_3$) δ 135.52 (d, *J* = 2.9 Hz), 133.85 (d, *J* = 10.1 Hz), 130.79 (d, *J* = 12.7 Hz), 117.42 (d, *J* = 86.6 Hz), 83.03, 69.60, 31.16, 28.42, 27.22 (d, *J* = 2.9 Hz), 18.71 (d, *J* = 51.8 Hz), 13.55; HRMS: Calculated for $[C_{25}H_{24}N_2P]^+$ 383.1672, found 383.1681.

(But-3-yn-1-yloxy)(*tert*-butyl)dimethylsilane (12)

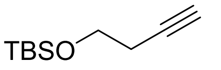

To a cooled (0 °C) solution of but-3-yn-1-ol (5.0 mL, 66 mmol) in dry DMF (150 mL) was added imidazole (6.75 g, 99 mmol) and subsequently TBSCl (11.95 g, 79 mmol) in portions. After 2 h, the reaction was quenched with H_2O (100 mL) and diluted with Et_2O (150 mL). The layers were separated and the aqueous layer extracted with Et_2O (150 mL). The combined organic layers were washed with brine (4 x 100 mL), dried over $MgSO_4$, filtered and concentrated under reduced pressure to afford the title compound as a clear liquid (11.95 g, 64.8 mmol, 98%). ^{1}H NMR (400 MHz, $CDCl_3$) δ 3.74 (t, *J* = 7.1 Hz, 2H),

2.40 (td, J = 7.1, 2.7 Hz, 2H), 1.96 (t, J = 2.7 Hz, 1H), 0.89 (s, 9H), 0.07 (s, 6H); ^{13}C NMR (101 MHz, $CDCl_3$) δ 81.66, 69.43, 61.87, 26.02, 22.98, -5.16. Spectra were consistent with previously reported data.[12]

5-((*Tert*-butyldimethylsilyl)oxy)pent-2-yn-1-ol (13)

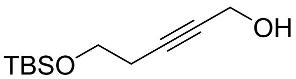

To a cooled (-40 °C) solution of alkyne **12** (26.76 g, 145 mmol) in dry THF (300 mL) under argon was added *n*-BuLi (61.0 mL, 152 mmol) dropwise over 10 min. After addition, it was stirred at -40 °C for 15 min and subsequently transferred through cannula to a flask containing a cooled (-40 °C) suspension of paraformaldehyde (13.08 g, 435 mmol) in dry THF (150 mL). After addition, the cooling bath was removed and the mixture was stirred for 1 h. It was then diluted with Et_2O (500 mL) and quenched by addition of brine (50 mL). The mixture was then washed with brine (150 mL) after which the organic layer was collected, dried over $MgSO_4$, filtered and concentrated under reduced pressure. The residue was purified through column chromatography (EtOAc/pentane = 1:19 to 1:4) to afford the title compound as a clear oil (25.46 g, 119 mmol, 82%). R_f = 0.22 (EtOAc/pentane = 1:9); ^{1}H NMR (400 MHz, $CDCl_3$) δ 4.23 (t, J = 2.2 Hz, 2H), 3.71 (t, J = 7.2 Hz, 2H), 2.42 (tt, J = 7.2, 2.1 Hz, 2H), 1.95 (bs, 1H), 0.89 (s, 9H), 0.06 (s, 6H); ^{13}C NMR (101 MHz, $CDCl_3$) δ 83.43, 79.63, 61.95, 51.39, 26.00, 23.23, 18.47, -5.16. Spectra were consistent with previously reported data.[13]

5-((*Tert*-butyldimethylsilyl)oxy)pent-2-yn-1-yl 4-methylbenzenesulfonate (14)

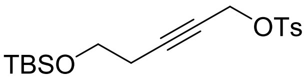

To a cooled (0 °C) solution of alcohol **13** (8.220 g, 38.3 mmol) in Et_2O (80 mL) was added TsCl (8.77 g, 46.0 mmol) followed by freshly pestled KOH (10.76 g, 192 mmol) in 10 portions over 5 min. The reaction was removed from the ice bath and after 45 min it was diluted with Et_2O (120 mL), cooled to 0 °C and poured into ice water (200 mL). The layers were separated and the aq. layer extracted with Et_2O (200 mL). The combined organic layers were washed with brine, dried over $MgSO_4$, filtered and concentrated under reduced pressure to afford the crude product (12.9622 g) as a slightly orange oil which was used without further purification due to its instability. R_f = 0.69 (EtOAc/pentane = 1:9); For NMR characterization an analytical sample was purified by column chromatography (EtOAc/pentane = 1:24); ^{1}H NMR (400 MHz, $CDCl_3$) δ 7.80 (d, J = 8.4 Hz, 2H), 7.34 (d, J = 8.0 Hz, 2H), 4.67 (t, J = 2.2 Hz, 2H), 3.59 (t, J = 7.1 Hz, 2H), 2.44 (s, 3H), 2.29 (tt, J = 7.1, 2.2 Hz, 2H), 0.87 (s, 9H), 0.03 (s, 6H); ^{13}C NMR (101 MHz, $CDCl_3$) δ 145.04, 133.37, 129.86, 128.24, 87.58, 73.03, 61.36, 58.66, 25.94, 23.17, 21.78, 18.40, -5.21. Spectra were consistent with previously reported data.[14]

9-((*Tert*-butyldimethylsilyl)oxy)nona-3,6-diyn-1-ol (15)

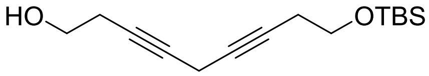

In a roundbottom flask, CuI (7.62 g, 40.0 mmol), NaI (6.00 g, 40.0 mmol) and Cs_2CO_3 (13.04 g, 40.0 mmol) were dried under vacuum at 90 °C for 2 h. The flask was backfilled with nitrogen and allowed to reach rt. Dry degassed DMF (70 mL) was added and the mixture was stirred for 10 min. But-3-yn-1-ol (3.22 mL, 42.6 mmol) was added to the mixture which was then stirred for 10 min. Sulfonate ester **14** (13.08 g, 35.5 mmol) was then added dropwise and the reaction was stirred overnight in the dark. The reaction was quenched with sat. aq. NH_4Cl (15 mL) and diluted with Et_2O (150 mL). After 20 min of stirring, the mixture was filtered over pad of celite and sand. The pad was rinsed with Et_2O (800 mL) and the resulting solution was washed with sat. aq. NH_4Cl (200 mL) and water (200 mL). The combined aq. layers were extracted with Et_2O (200 mL) and the combined organic layers were washed with brine (400 mL), dried over $MgSO_4$, filtered and concentrated under reduced pressure. Column chromatography (Et_2O/pentane = 1:10 to 1:4) afforded the title compound as a yellow oil (8.170 g, 30.7 mmol, 86%). R_f = 0.36 (EtOAc/pentane = 1:4); ^{1}H NMR (400 MHz, $CDCl_3$) δ 3.72 – 3.66 (m, 4H), 3.12 (p, J = 2.4 Hz, 2H), 2.43 (tt, J = 6.2, 2.4 Hz, 2H), 2.37 (tt, J = 7.2, 2.4 Hz, 2H), 0.88 (s, 9H), 0.06 (s, 6H); ^{13}C NMR (101 MHz, $CDCl_3$) δ 77.80, 77.07, 76.74, 75.37, 62.08, 61.22, 26.02, 23.24, 23.22, 18.49, 9.89, -5.13; HRMS: Calculated for $[C_{15}H_{26}O_2Si+H]^+$ 267.1775, found 267.1775.

(3*Z*,6*Z*)-9-((*Tert*-butyldimethylsilyl)oxy)nona-3,6-dien-1-ol (9)

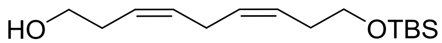

In a Schlenk flask, nickel acetate hydrate (0.300 g, 1.206 mmol) was added to 96% EtOH (15.08 mL) and purged with nitrogen. In a vial, $NaBH_4$

(0.046 g, 1.206 mmol) was added to a mixture of 96% EtOH (0.2 mL) and aq. NaOH (2 M, 11 μL) and the mixture was added dropwise to the Schlenk flask. The flask was purged with H_2 and stirred for 20 min. Then, freshly distilled ethylenediamine (0.244 mL, 3.62 mmol) and alcohol **15** (1.6071 g, 6.03 mmol) were added and the reaction was stirred under H_2 atmosphere. After 2 h, additional catalyst was prepared in a Schlenk flask in 96% EtOH (5 mL) and added to the reaction via cannula. After 20 min of stirring the reaction was purged with N_2, the mixture was diluted with Et_2O (80 mL) and water (80 mL) and filtered over celite. The layers were separated, the organic layer was washed with water (80 mL) and the combined aq. layers were extracted with Et_2O (100 mL). The combined organic layers were washed with water (100 mL) and brine (100 mL), dried over $MgSO_4$, filtered and concentrated under reduced pressure. Column chromatography (EtOAc/pentane = 1:14.5 to 1:10.5) afforded the title compound (1.0363 g, 3.83 mmol, 64%) as a yellow oil. R_f = 0.69 (EtOAc/pentane = 1:2); ^{1}H NMR (400 MHz, $CDCl_3$) δ 5.58 – 5.49 (m, 1H), 5.47 – 5.35 (m, 3H), 3.65 (t, *J* = 6.5 Hz, 2H), 3.62 (t, *J* = 6.9 Hz, 2H), 2.84 (t, *J* = 5.7 Hz, 2H), 2.41 – 2.26 (m, 4H), 0.89 (s, 9H), 0.05 (s, 6H); ^{13}C NMR (101 MHz, $CDCl_3$) δ 131.42, 129.52, 126.55, 125.67, 62.98, 62.35, 31.25, 30.97, 26.10, 26.02, -5.12; HRMS: Calculated for $[C_{15}H_{30}O_2Si+H]^+$ 271.2088, found 271.2090.

Methyl pent-4-ynoate (17)

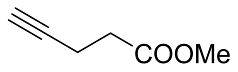

To a cooled (0 °C) solution of pent-4-ynoic acid (5.14 g, 52.4 mmol) in dry MeOH (200 mL) was added $SOCl_2$ (4.21 mL, 57.6 mmol) dropwise after which the reaction was allowed to reach rt overnight. The reaction was diluted with DCM (250 mL) and sat. aq. $NaHCO_3$ (500 mL). The layers were separated and the aq. layer extracted with DCM (200 mL). The combined organic layers were washed with brine (200 mL), dried over $MgSO_4$, filtered, concentrated under reduced pressure and coevaporated with DCM to afford the title compound as a clear oil (5.39 g, 48.1 mmol, 92%). R_f = 0.47 (EtOAc/pentane 1:19); ^{1}H NMR (400 MHz, $CDCl_3$) δ 3.71 (s, 3H), 2.60 – 2.54 (m, 2H), 2.54 – 2.48 (m, 2H), 1.99 (t, *J* = 2.5 Hz, 1H); ^{13}C NMR (101 MHz, $CDCl_3$) δ 172.31, 82.55, 69.12, 51.92, 33.23, 14.44. Spectra were consistent with previously reported data.[15]

Methyl 10-((*tert*-butyldimethylsilyl)oxy)deca-4,7-diynoate (18)

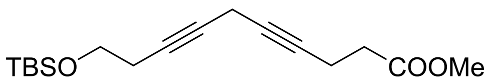

In a roundbottom flask, CuI (7.29 g, 38.3 mmol), NaI (5.74 g, 38.3 mmol) and Cs_2CO_3 (12.48 g, 38.3 mmol) were dried under vacuum for 6 h at 95 °C. The flask was refilled with nitrogen and allowed to cool to rt. Under stirring, dry degassed DMF (70 mL) was added and the mixture was stirred for 10 min. Then, alkyne **17** (5.389 g, 48.1 mmol) was added in one portion after which sulfonate ester **14** (12.97 g, 35.2 mmol) was added dropwise over 15 min and the reaction was stirred overnight. The reaction was diluted with Et_2O (320 mL) and quenched with sat. aq. NH_4Cl (12 mL) and stirred for 15 min. The mixture was filtered over celite and the filter was rinsed with Et_2O (1.5 L). The mixture was washed with sat. aq. NH_4Cl (100 mL) and water (100 mL), the combined aq. layers were extracted with Et_2O (100 mL) and the combined organic layers were washed with brine (5 x 100 mL). The organic layer was dried over $MgSO_4$, filtered and concentrated under reduced pressure. The residue was purified using neutralized silica (flushed with 0.5% Et_3N in pentane, EtOAc/pentane = 1:99 to 1:24) to afford the title compound as a clear oil (8.050 g, 26.1 mmol, 74%). R_f = 0.54 (EtOAc/pentane = 1:9); ^{1}H NMR (400 MHz, $CDCl_3$) δ 3.73 – 3.65 (m, 5H), 3.09 (t, *J* = 2.3 Hz, 2H), 2.55 – 2.46 (m, 4H), 2.40 – 2.33 (m, 2H), 0.88 (s, 9H), 0.06 (s, 6H); ^{13}C NMR (101 MHz, $CDCl_3$) δ 172.57, 78.55, 77.63, 75.45, 75.27, 62.09, 51.87, 33.49, 26.01, 23.22, 18.47, 14.76, 9.83, -5.15; HRMS: Calculated for $[C_{17}H_{28}O_3Si+H]^+$ 309.1881, found 309.1882.

Methyl (4*Z*,7*Z*)-10-((*tert*-butyldimethylsilyl)oxy)deca-4,7-dienoate (19)

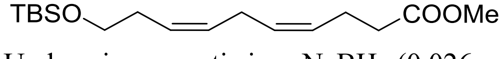

In a microwave vial, nickel acetate hydrate (0.169 g, 0.681 mmol) was dissolved in MeOH (2 mL) and purged with three vacuum/H_2 cycles. Under vigorous stirring, $NaBH_4$ (0.026 g, 0.681 mmol) was added in dry MeOH (1 mL). After 15 min, freshly distilled ethylenediamine (0.184 mL, 2.72 mmol) was added and the mixture was purged with a cycle of vacuum/H_2. After 15 min a solution of methyl ester **18** in MeOH (1 mL) was added and the mixture was purged with a cycle of vacuum/H_2. After 3 h, the mixture was purged with three vacuum/N_2 cycles, filtered over celite and concentrated under reduced pressure. The residue was redissolved in Et_2O (10 mL), washed with sat. aq. NH_4Cl (5 mL) and brine

(5 mL), dried over Na_2SO_4, filtered and concentrated under reduced pressure to afford the title compound as a yellow oil (0.0634 g, 0.203 mmol, 89%). R_f = 0.62 (EtOAc/pentane = 1:24); ^{1}H NMR (400 MHz, $CDCl_3$) δ 5.45 – 5.30 (m, 4H), 3.67 (s, 3H), 3.61 (t, J = 6.9 Hz, 2H), 2.81 (t, J = 5.8 Hz, 2H), 2.42 – 2.33 (m, 4H), 2.33 – 2.24 (m, 2H), 0.89 (s, 9H), 0.05 (s, 6H); ^{13}C NMR (101 MHz, $CDCl_3$) δ 173.68, 129.63, 129.56, 127.87, 126.44, 62.98, 51.68, 34.15, 31.24, 26.09, 25.82, 22.92, 18.50, -5.13; HRMS: Calculated for $[C_{17}H_{32}O_3Si\text{-}TBS+2H]^+$ 199.1329, found 199.1328.

Methyl (4*Z*,7*Z*)-10-hydroxydeca-4,7-dienoate (20)

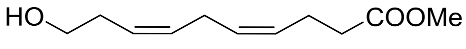

To a cooled (0 °C) solution of methyl ester **19** (0.7420 g, 2.374 mmol) in THF (25 mL) was added TBAF (1 M in THF, 3.56 mL, 3.56 mmol) and the mixture was allowed to reach rt. After 2 h, additional TBAF (1 M in THF, 0.475 mL, 0.475 mmol) was added. After 0.5 h the reaction was cooled to 0 °C, diluted with sat. aq. NH_4Cl (100 mL) and Et_2O (100 mL). The organic phase was collected and the aq. phase extracted with Et_2O (100 mL). The combined organic layers were washed with brine (2 x 100 mL), dried over $MgSO_4$, filtered and concentrated under reduced pressure. Column chromatography (EtOAc/pentane = 1:9 to 1:1) afforded the title compound as a clear oil (0.3361 g, 1.695 mmol, 71%). R_f = 0.52 (EtOAc/pentane = 1:1); ^{1}H NMR (500 MHz, $CDCl_3$) δ 5.59 – 5.49 (m, 1H), 5.48 – 5.33 (m, 3H), 3.70 – 3.62 (m, 5H), 2.90 – 2.80 (m, 2H), 2.46 – 2.33 (m, 6H), 1.69 (s, 1H); ^{13}C NMR (126 MHz, $CDCl_3$) δ 173.81, 130.99, 129.31, 128.07, 125.93, 62.31, 51.74, 34.08, 30.98, 25.84, 22.93; HRMS: Calculated for $[C_{11}H_{18}O_3+H]^+$ 199.1329, found 199.1329.

Methyl (4*Z*,7*Z*)-10-bromodeca-4,7-dienoate (21)

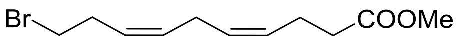

Alcohol **20** (0.4527 g, 2.283 mmol) was coevaporated three times with dry toluene, dissolved in dry DCM (10 mL) and cooled to -30 °C. CBr_4 (0.984 g, 2.97 mmol) was added and then PPh_3 (0.779 g, 2.97 mmol) was added in three portions over three min. After 30 min, the reaction was allowed to reach 0 °C and stirred for 1.5 h. The solvent was then removed under reduced pressure and the residue purified with column chromatography (Et_2O/pentane = 1:19 to 1:9) to afford the title compound as a clear oil (0.6031 g, 2.309 mmol, quant.). R_f = 0.71 (EtOAc/pentane = 1:19); ^{1}H NMR (400 MHz, $CDCl_3$) δ 5.57 – 5.47 (m, 1H), 5.47 – 5.32 (m, 3H), 3.68 (s, 3H), 3.38 (t, J = 7.1 Hz, 2H), 2.83 (t, J = 5.9 Hz, 2H), 2.65 (q, J = 7.1 Hz, 2H), 2.39 (m, 4H); ^{13}C NMR (101 MHz, $CDCl_3$) δ 173.58, 130.84, 128.94, 128.27, 126.53, 51.68, 34.03, 32.49, 30.86, 25.83, 22.90; HRMS: Calculated for $[C_{11}H_{17}BrO_2+H]^+$ 261.0485, found 261.0486.

((3*Z*,6*Z*)-10-Methoxy-10-oxodeca-3,6-dien-1-yl)triphenylphosphonium bromide (8)

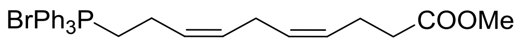

In a microwave vial, bromide **21** (0.2803 g, 1.073 mmol) and dry PPh_3 (0.422 g, 1.610 mmol) were dissolved in dry acetonitrile (7 mL) and the vial was sealed. The solvent was purged with three vacuum/N_2 cycles and the reaction was stirred at 92 °C for 5 days in the dark. The reaction was concentrated under a flow of N_2 and purified with column chromatography (MeOH/DCM = 1:99 to 1:5) to afford the title compound as a clear syrup (0.5751 g, 1.099 mmol, quant.). R_f = 0.19 (MeOH/DCM 1:9); ^{1}H NMR (500 MHz, $CDCl_3$) δ 7.92 – 7.77 (m, 10H), 7.75 – 7.72 (m, 5H), 5.69 – 5.56 (m, 1H), 5.42 – 5.32 (m, 1H), 5.32 – 5.22 (m, 2H), 3.91 – 3.81 (m, 2H), 3.63 (s, 3H), 2.58 (t, J = 7.0 Hz, 2H), 2.54 – 2.39 (m, 2H), 2.30 (td, J = 7.2, 1.2 Hz, 2H), 2.26 – 2.18 (m, 2H); ^{13}C NMR (126 MHz, $CDCl_3$) δ 173.41, 135.13 (d, J = 3.1 Hz), 133.68 (d, J = 10.1 Hz), 130.54 (d, J = 12.6 Hz), 130.17, 128.33 (d, J = 13.3 Hz), 126.54 (d, J = 14.7 Hz), 118.10 (d, J = 85.8 Hz) 51.53, 33.70, 25.49, 22.91 (d, J = 48.7 Hz), 22.69, 20.40 (d, J = 3.5 Hz). HRMS: Calculated for $[C_{29}H_{32}O_2P]^+$ 443.2134, found 443.2132.

Methyl (4*Z*,7*Z*,10*Z*,13*Z*,16*Z*)-19-((*tert*-butyldimethylsilyl)oxy)nonadeca-4,7,10,13,16-pentaenoate (24)

To a cooled (0 °C) solution of alcohol **9** (0.2784 g, 1.029 mmol) in dry DCM (4 mL) was added DMP (0.655 g,

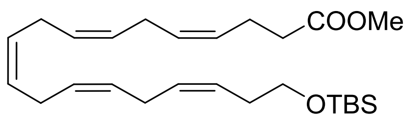

1.544 mmol) and the reaction was allowed to reach rt. After 30 min, the mixture was cooled to 0 °C, diluted with Et_2O (10 mL) and quenched with a mixture of sat. aq. $NaHCO_3$/10% (w/v) aq. $Na_2S_2O_3$ (1:1 (v/v), 10 mL) under vigorous stirring. The layers were separated and the organic layer washed with water (3 x 10 mL) and brine (10 mL), dried over Na_2SO_4, filtered and

concentrated under reduced pressure at rt. The generated aldehyde **23** was used in the next reaction immediately. Phosphonium salt **8** (0.9028 g, 1.725 mmol) was dried by coevaporation with dry toluene four times and dissolved in dry THF (9 mL) and dry HMPA (1.7 mL). It was cooled to -60 °C after which LiHMDS (1 M in THF, 1.65 mL, 1.65 mmol) was added dropwise. The reaction was stirred for 40 min at -60 °C and then cooled to -100 °C. A solution of aldehyde **23** in dry THF (2 mL) was added dropwise to the reaction. The reaction was stirred at -100 °C for 30 min and then allowed to reach 0 °C over 3 h. It was cooled to -20 °C and quenched with sat. aq. $NaHCO_3$ (10 mL). It was diluted with Et_2O (50 mL) and sat. aq. $NaHCO_3$ (20 mL), the aq. layer was isolated and extracted with Et_2O (30 mL) and the combined organic layers were washed with brine (3 x 20 mL), dried over Na_2SO_4, filtered and concentrated under reduced pressure. Column chromatography (Et_2O/pentane = 1:30 to 1:15) allowed separation from the undesired trans product and afforded the title compound as a clear oil (0.2529 g, 0.584 mmol, 57%). R_f = 0.57 (EtOAc/pentane = 1:20); ^{1}H NMR (400 MHz, $CDCl_3$) δ 5.50 – 5.29 (m, 10H), 3.67 (s, 3H), 3.62 (t, J = 7.0 Hz, 2H), 2.84 (dd, J = 7.0, 3.2 Hz, 8H), 2.38 (q, J = 3.1, 2.6 Hz, 4H), 2.30 (q, J = 6.9 Hz, 2H), 0.89 (s, 9H), 0.05 (s, 6H); ^{13}C NMR (101 MHz, $CDCl_3$) δ 129.68, 129.46, 128.49, 128.38, 128.36, 128.25, 128.20, 128.18, 128.01, 126.36, 63.00, 51.70, 34.15, 31.26, 26.10, 25.90, 25.77, 25.71, 22.93, 18.51, -5.12; HRMS: Calculated for $[C_{26}H_{44}O_3Si+H]^+$ 433.3133, found 433.3132.

Methyl (4*Z*,7*Z*,10*Z*,13*Z*,16*Z*)-19-hydroxynonadeca-4,7,10,13,16-pentaenoate (7)

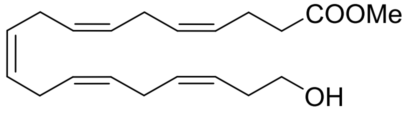

To a cooled (0 °C) solution of methyl ester **24** (0.5385 g, 1.244 mmol) in THF (12 mL) was added TBAF (1 M in THF, 1.87 mL, 1.87 mmol) and the reaction was allowed to reach rt. After 2 h, the reaction was quenched with sat. aq. NH_4Cl (100 mL) and diluted with Et_2O (100 mL). The layers were separated and the aq. layer was extracted with Et_2O (2 x 50 mL). The combined organic layers were washed with brine (100 mL), dried over $MgSO_4$, filtered and concentrated under reduced pressure. The residue was purified with column chromatography (EtOAc/pentane = 1:9 to 1:4) to afford the title compound (0.3453 g, 1.084 mmol, 87%) as a clear oil which was further purified with preparative HPLC (Gemini, 63 to 66% B in A over 12 min, 5 mL/min). R_f = 0.70 (EtOAc/pentane = 1:1); ^{1}H NMR (500 MHz, $CDCl_3$) δ 5.59 – 5.50 (m, 1H), 5.47 – 5.32 (m, 9H), 3.72 – 3.61 (m, 5H), 2.92 – 2.79 (m, 8H), 2.44 – 2.32 (m, 6H), 1.72 (bs, 1H); ^{13}C NMR (126 MHz, $CDCl_3$) δ 173.77, 131.17, 129.46, 128.41, 128.36, 128.31, 128.26, 128.21, 128.15, 128.00, 125.82, 62.34, 51.73, 34.13, 30.97, 25.90, 25.79, 25.78, 25.71, 22.92; HRMS: Calculated for $[C_{20}H_{30}O_3+H]^+$ 319.2268, found 319.2269.

Methyl (4*Z*,7*Z*,10*Z*,13*Z*,16*Z*,19*Z*)-21-(3-(but-3-yn-1-yl)-3*H*-diazirin-3-yl)henicosa-4,7,10,13,16,19-hexaenoate (26)

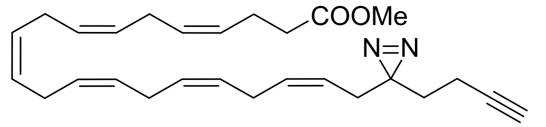

Alcohol **7** (0.0613 g, 0.192 mmol) was dissolved in dry DCM (2 mL), cooled to 0 °C and DMP (0.122 g, 0.289 mmol) was added. It was allowed to reach rt and stirred for 1 h, after which it was cooled to 0 °C, diluted with Et_2O (5 mL) and quenched by addition of a mixture of sat. aq. $NaHCO_3$/10% (w/v) aq. $Na_2S_2O_3$ (1:1 (v/v), 4 mL). It was stirred at rt until two layers appeared, after which the layers were separated, the aq. layer was extracted with Et_2O (10 mL) and the combined organic layers washed with brine (2 x 5 mL), dried over Na_2SO_4, filtered and concentrated under reduced pressure at rt. The formed aldehyde **25** was dried by coevaporation with toluene at room temperature and used immediately. Phosphonium salt **6** (0.128 g, 0.250 mmol) was dried by coevaporation with toluene three times. It was suspended in dry THF (3 mL) and cooled to -70 °C after which KO*t*Bu (1.0 M in THF, 0.202 mL, 0.202 mmol) was added dropwise and the reaction was allowed to reach -50 °C over 1 h, then cooled to -105 °C. The aldehyde was dissolved in dry THF (2 mL) and added to the reaction via cannula dropwise. The reaction was allowed to reach -30 °C after which it was cooled to -80 °C. The reaction was diluted with dry Et_2O (5 mL) and quenched with sat. aq. $NaHCO_3$ (2 mL) under vigorous stirring. The reaction was further diluted with Et_2O (25 mL) and sat. aq. $NaHCO_3$ (25 mL), the layers were separated and the aq. layer extracted with Et_2O (25 mL). The combined organic layers were washed with brine (50 mL), dried over $MgSO_4$, filtered and concentrated under reduced pressure. Column chromatography of the residue (Et_2O/pentane = 1:49 to 1:19) allowed separation from the undesired trans product and afforded the

title compound as a yellow oil (0.0306 g, 0.073 mmol, 38%). R_f = 0.68 (EtOAc/pentane = 1:9); ^{1}H NMR (500 MHz, $CDCl_3$) δ **5.53 (dtt, *J* = 10.4, 2.0, 7.2, 1H)**, 5.45 – 5.30 (m, 10H), **5.24 (dtt, *J* = 10.4, 1.6, 7.6 Hz, 1H)**, 3.67 (s, 3H), 2.89 – 2.79 (m, 8H), 2.75 (t, *J* = 7.3 Hz, 2H), 2.43 – 2.35 (m, 4H), 2.17 (d, *J* = 7.6 Hz, 2H), 2.04 – 1.98 (m, 3H), 1.65 (t, *J* = 7.4 Hz, 2H); ^{13}C NMR (126 MHz, $CDCl_3$) δ 173.66, 132.03, 129.43, 128.73, 128.42, 128.33, 128.25, 128.13, 128.03, 127.53, 121.94, 82.88, 69.26, 51.70, 34.13, 32.10, 31.27, 28.27, 25.86, 25.78, 25.71, 22.92, 13.45; HRMS: Calculated for $[C_{27}H_{36}N_2O_2+H]^+$ 421.2850, found 421.2849.

(4*Z*,7*Z*,10*Z*,13*Z*,16*Z*,19*Z*)-21-(3-(But-3-yn-1-yl)-3*H*-diazirin-3-yl)henicosa-4,7,10,13,16,19-hexaenoic acid (pac-DHA, 4)

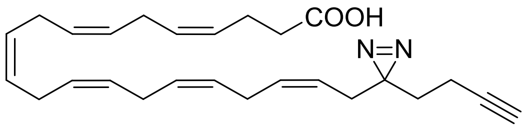

To a cooled (0 °C) solution of methyl ester **26** (0.0198 g, 0.047 mmol) in THF (2 mL) was added 1 M aq. LiOH (2 mL) and the reaction was allowed to reach rt overnight. It was then cooled to 0 °C, diluted with Et_2O (5 mL) and acidified with 1 M aq. HCl to pH <2. NaCl was added until saturation and the organic layer was isolated. The aq. layer was extracted with Et_2O (2 x 20 mL) and the combined organic layers were washed with brine (20 mL), dried over Na_2SO_4, filtered and concentrated under reduced pressure. Column chromatography of the residue (MeOH/DCM = 1:128 to 1:80) afforded the title compound as a slightly yellow oil (0.0159 g, 0.039 mmol, 83%). R_f = 0.47 (MeOH/DCM = 1:11); ^{1}H NMR (400 MHz, $CDCl_3$) δ 5.58 – 5.47 (m, 1H), 5.49 – 5.29 (m, 10H), 5.25 (dtt, *J* = 10.9, 7.6, 1.7 Hz, 1H), 2.84 (q, *J* = 6.9, 6.1 Hz, 8H), 2.75 (t, *J* = 7.0 Hz, 2H), 2.49 – 2.37 (m, 4H), 2.17 (dd, *J* = 7.6, 1.5 Hz, 2H), 2.05 – 1.96 (m, 3H), 1.65 (t, *J* = 7.3 Hz, 2H); ^{13}C NMR (101 MHz, $CDCl_3$) δ 178.86, 132.06, 129.72, 128.75, 128.44, 128.42, 128.32, 128.29, 128.16, 127.70, 127.55, 121.96, 82.91, 69.29, 33.99, 32.11, 31.29, 28.31, 25.88, 25.80, 25.74, 22.63, 13.48; HRMS: Calculated for $[C_{26}H_{34}N_2O_2+H]^+$ 407.2693, found 407.2693.

(4*Z*,7*Z*,10*Z*,13*Z*,15*E*,19*Z*)-21-(3-(But-3-yn-1-yl)-3*H*-diazirin-3-yl)-17-hydroxyhenicosa-4,7,10,13,15,19-hexaenoic acid (pac-17-HDHA, 5)

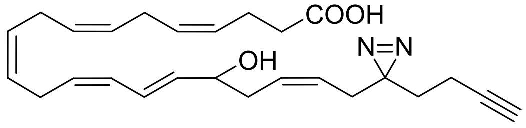

Carboxylic acid **4** (pac-DHA, 21 mg, 0.052 mmol) was dissolved in EtOH (5 mL) and added to a cooled (0 °C) borate buffer (50 mM boric acid in MilliQ, adjusted to pH 12 with NaOH, 500 mL). A solution of soybean lipoxidase (SBLOX, L7395, Sigma, 50 mg) in borate buffer was added under vigorous stirring and air bubbling, and the reaction was stirred for 20 min. Then, aq. $NaBH_4$ (10.33 mmol, 10.33 mL) was added and the reaction was stirred for 15 min. Then, acetic acid (2.63 mL, 45.9 mmol) was added dropwise and after 15 min the reaction was extracted with $CHCl_3$ (4 x 200 mL). The combined organic layers were dried over Na_2SO_4, filtered, concentrated under reduced pressure and purified with column chromatography (MeOH/DCM = 1:99) and preparative HPLC (Nucleodur, 66 to 72% B in A over 12 min, 5 mL/min) to afford the title compound as a clear oil (1.0 mg, 2.37 µmol, 4.6%). Aliquots were taken to analyze purity by LC-MS and NMR and regioselectivity was confirmed by the *m/z* 285 fragment ion after hydrogenation and LC-MS. ^{1}H NMR (500 MHz, $CDCl_3$) δ 6.57 (dd, J = 15.1, 11.0 Hz, 1H), 5.98 (t, J = 10.9 Hz, 1H), 5.64 (dd, J = 15.2, 6.3 Hz, 1H), 5.58 – 5.32 (m, 9H), 4.16 – 4.09 (m, 1H), 2.97 (t, J = 7.0 Hz, 2H), 2.91 – 2.80 (m, 4H), 2.45 – 2.39 (m, 4H), 2.07 – 1.98 (m, 5H), 1.76 – 1.68 (m, 4H), 1.62 – 1.58 (m, 1H). HRMS: Calculated for $[C_{26}H_{34}N_2O_3+Na]^+$ 445.2462, found 445.2459.

^{1}H and ^{13}C NMR spectra

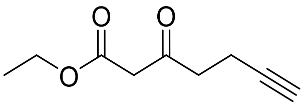

28

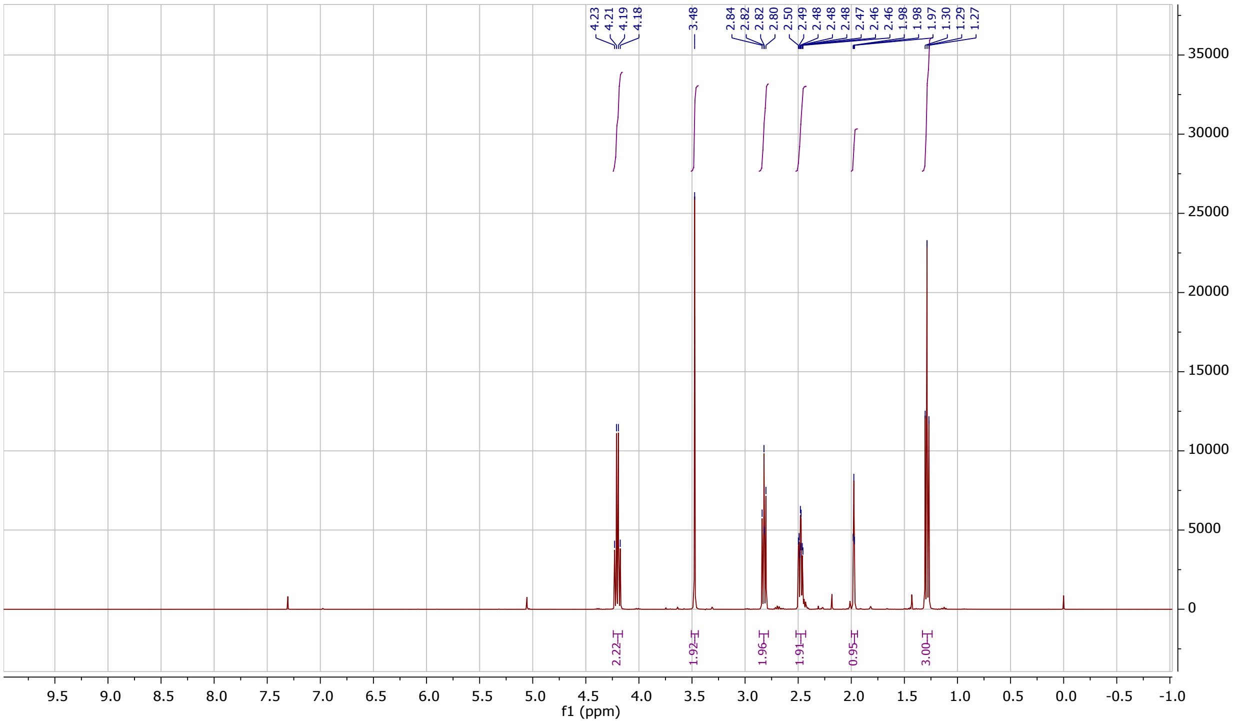

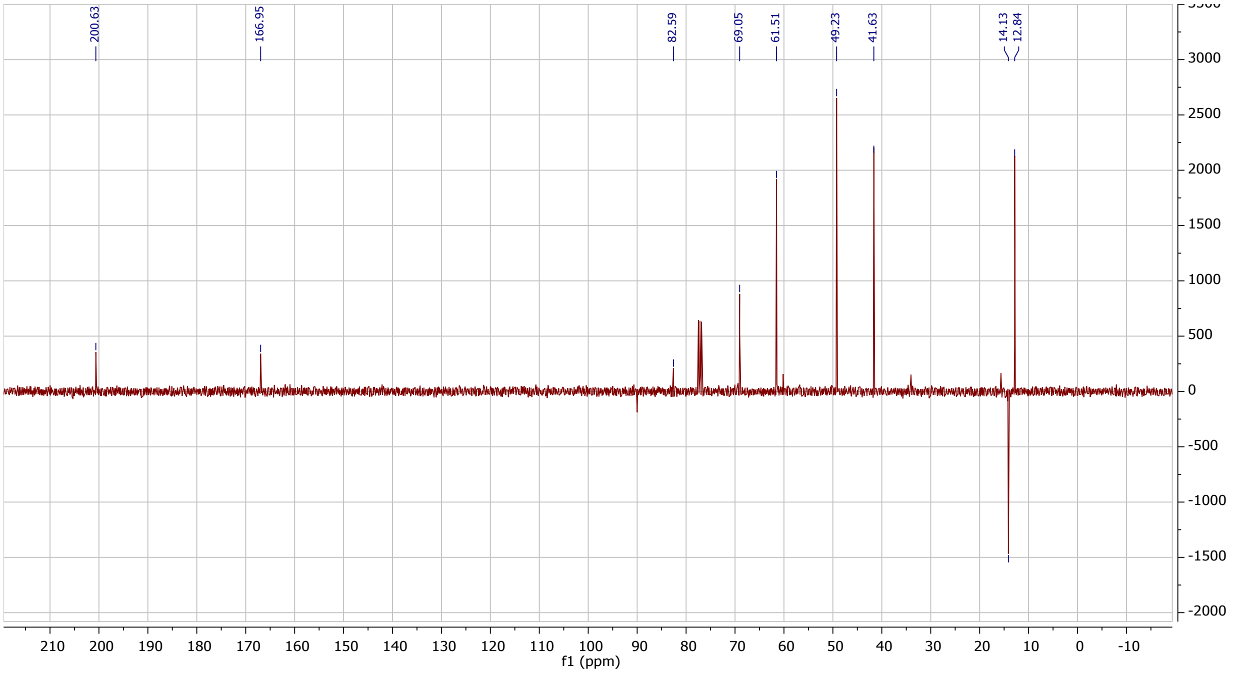

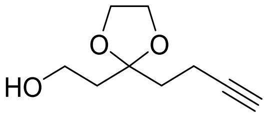
HO
O
O

29

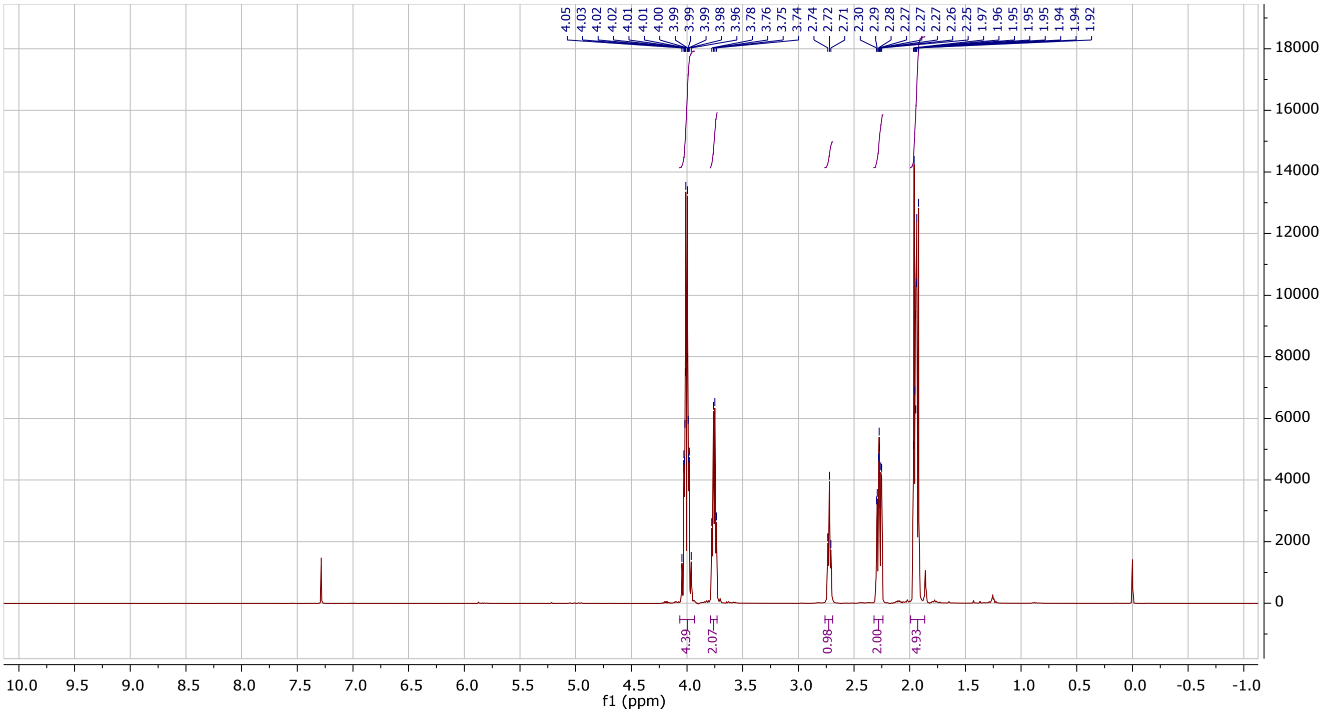
4.05
4.03
4.02
4.02
4.01
4.01
4.00
3.99
3.99
3.99
3.98
3.96
3.78
3.76
3.75
3.74
2.74
2.72
2.71
2.30
2.29
2.28
2.27
2.27
2.27
2.26
2.25
1.97
1.96
1.95
1.95
1.95
1.94
1.94
1.92
4.39
2.07
0.98
2.00
4.93
f1 (ppm)

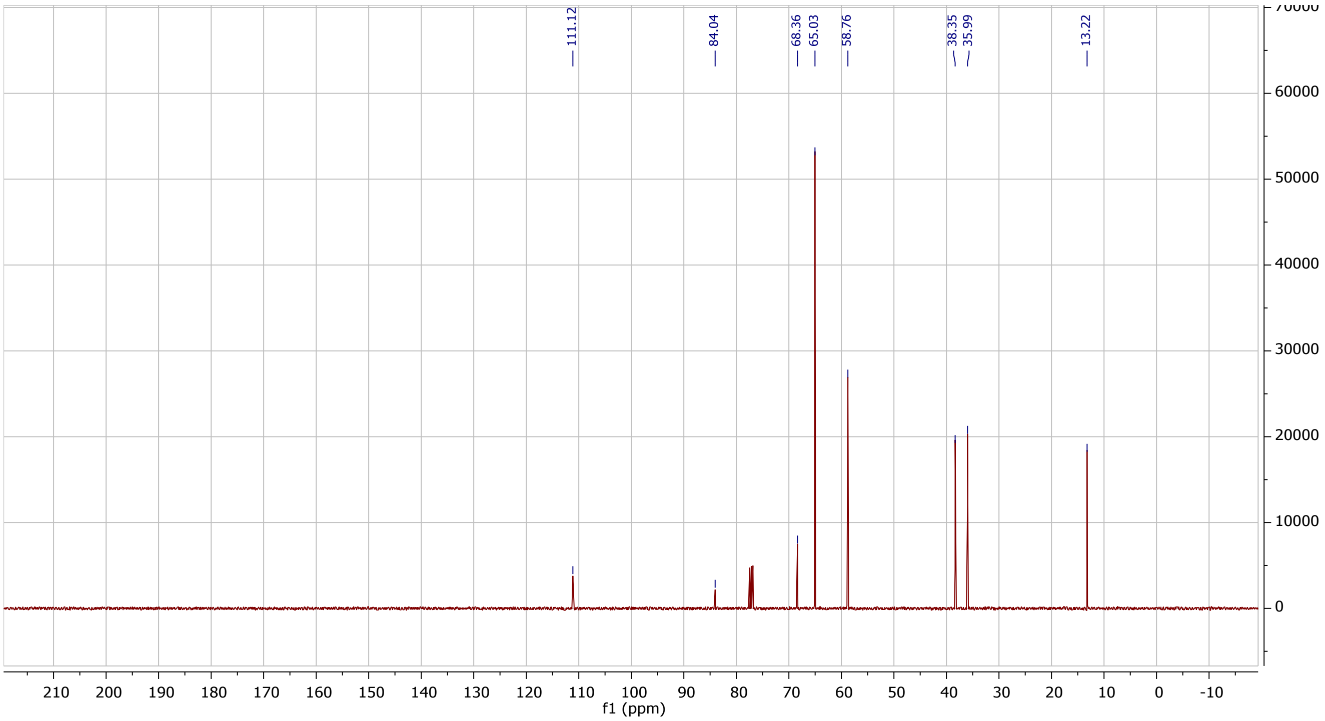
111.12
84.04
68.36
65.03
58.76
38.35
35.99
13.22
f1 (ppm)

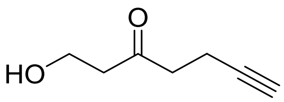
O
HO

30

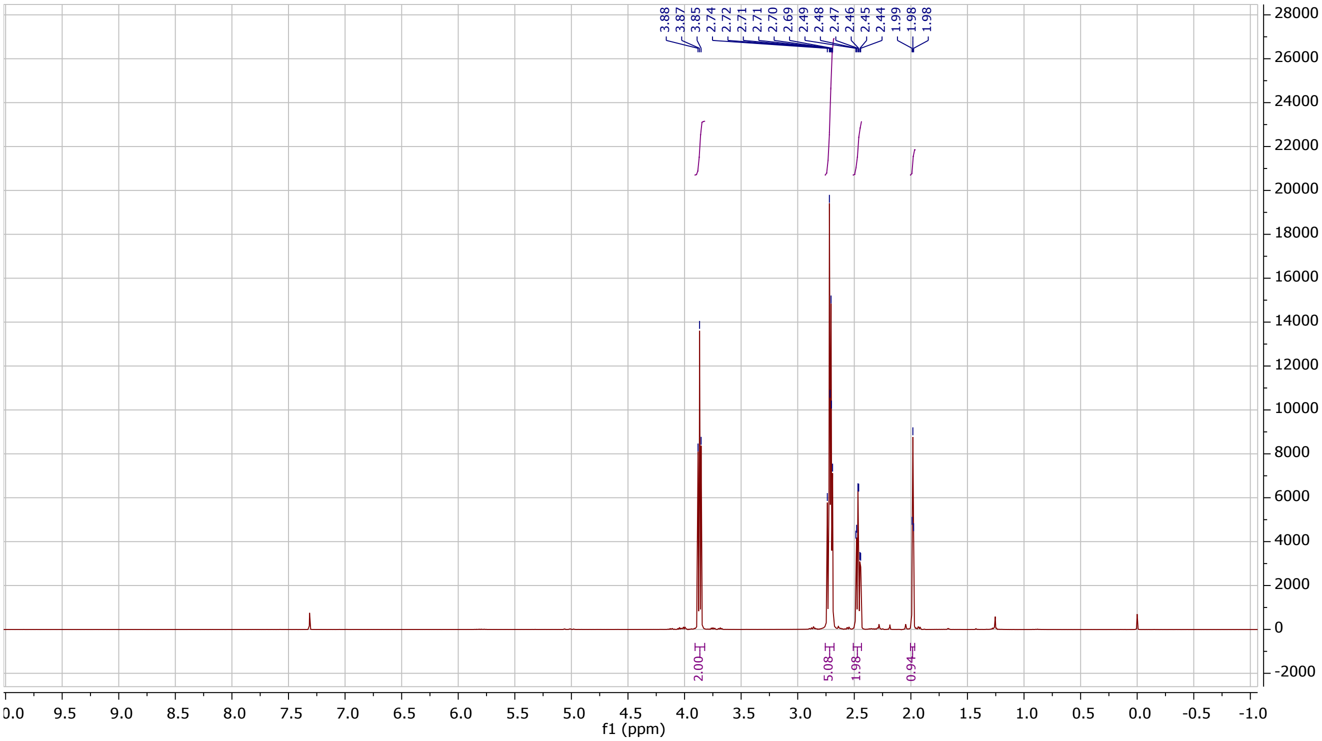
3.88
3.87
3.85
2.74
2.72
2.71
2.71
2.70
2.69
2.49
2.48
2.47
2.46
2.45
2.44
1.99
1.98
1.98
2.00
5.08
1.98
0.94
f1 (ppm)

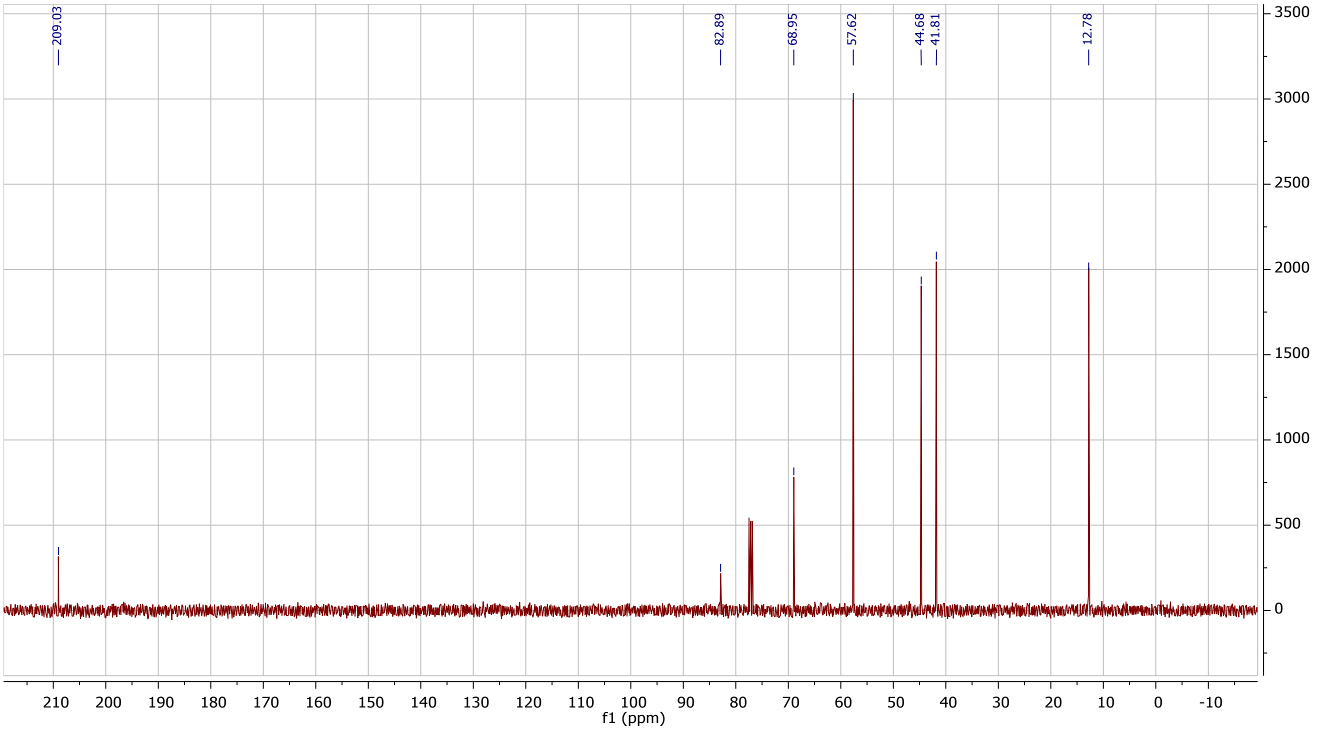
209.03
82.89
68.95
57.62
44.68
41.81
12.78
f1 (ppm)

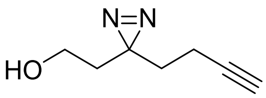

31

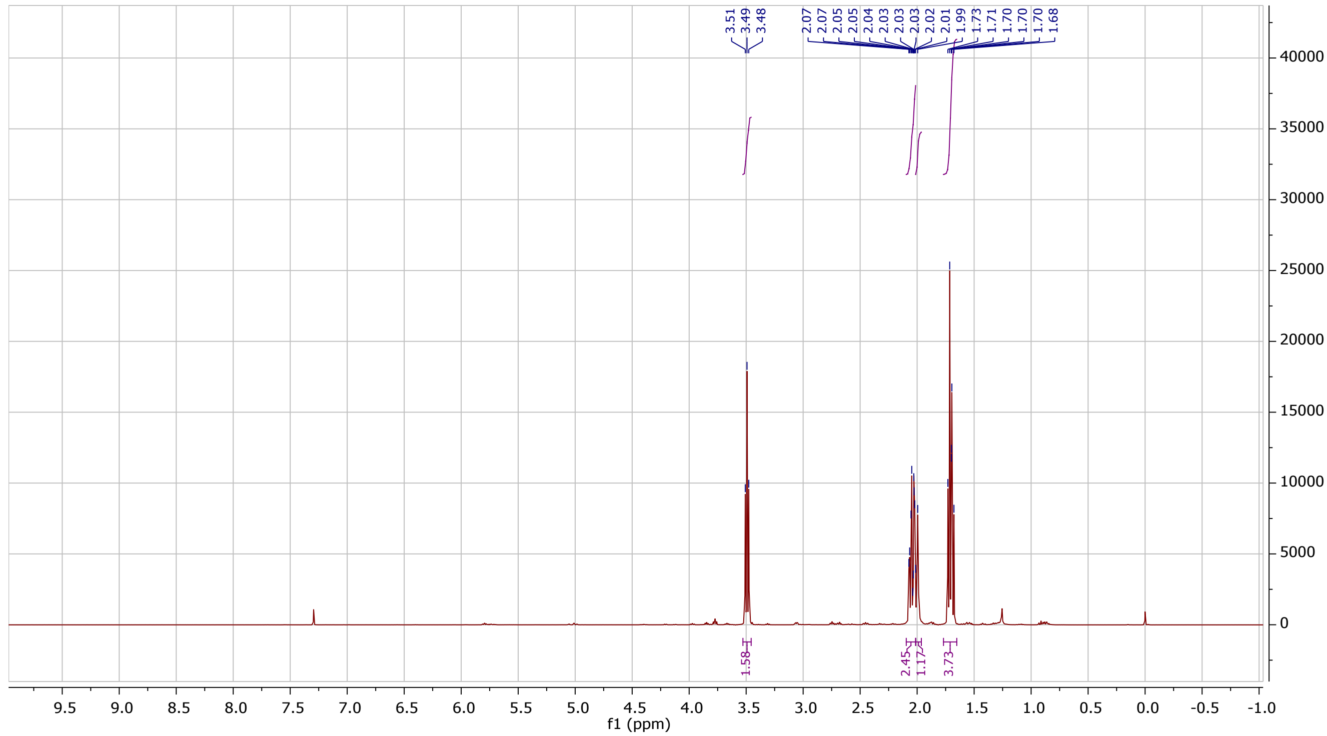

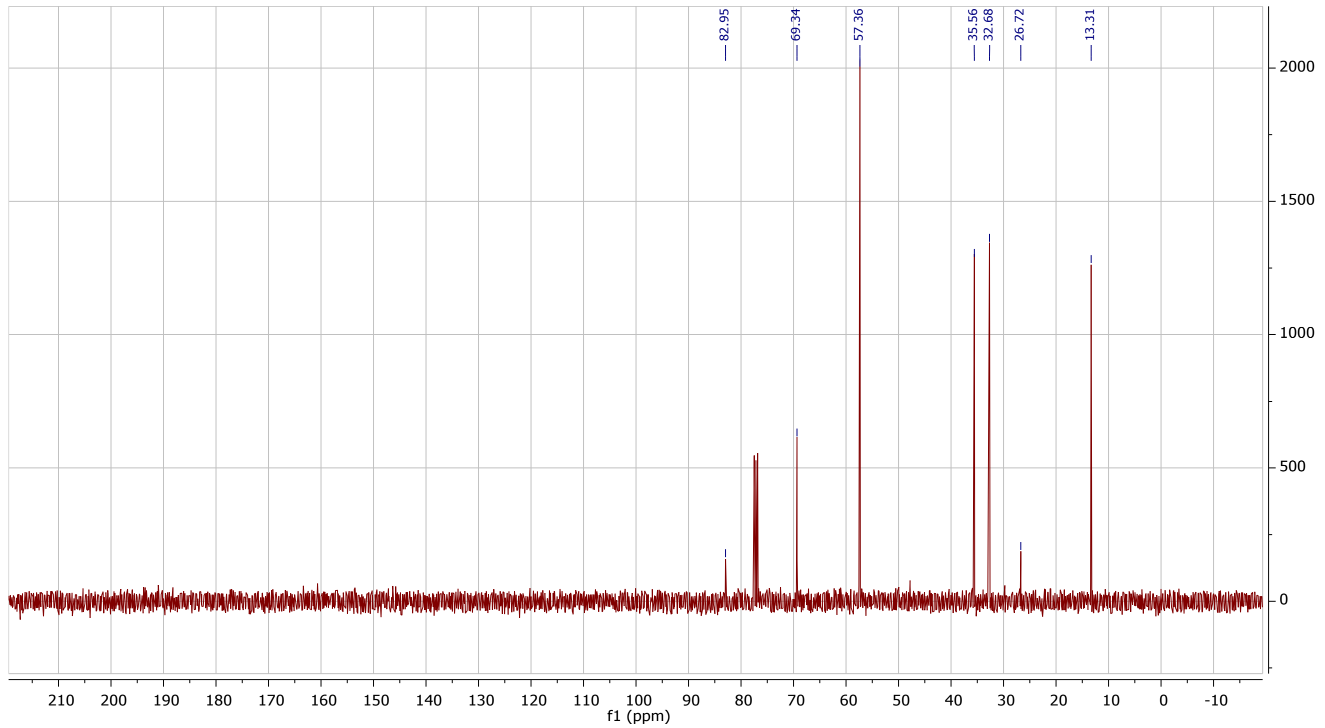

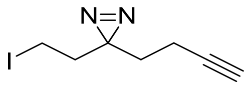

N=N
I

10

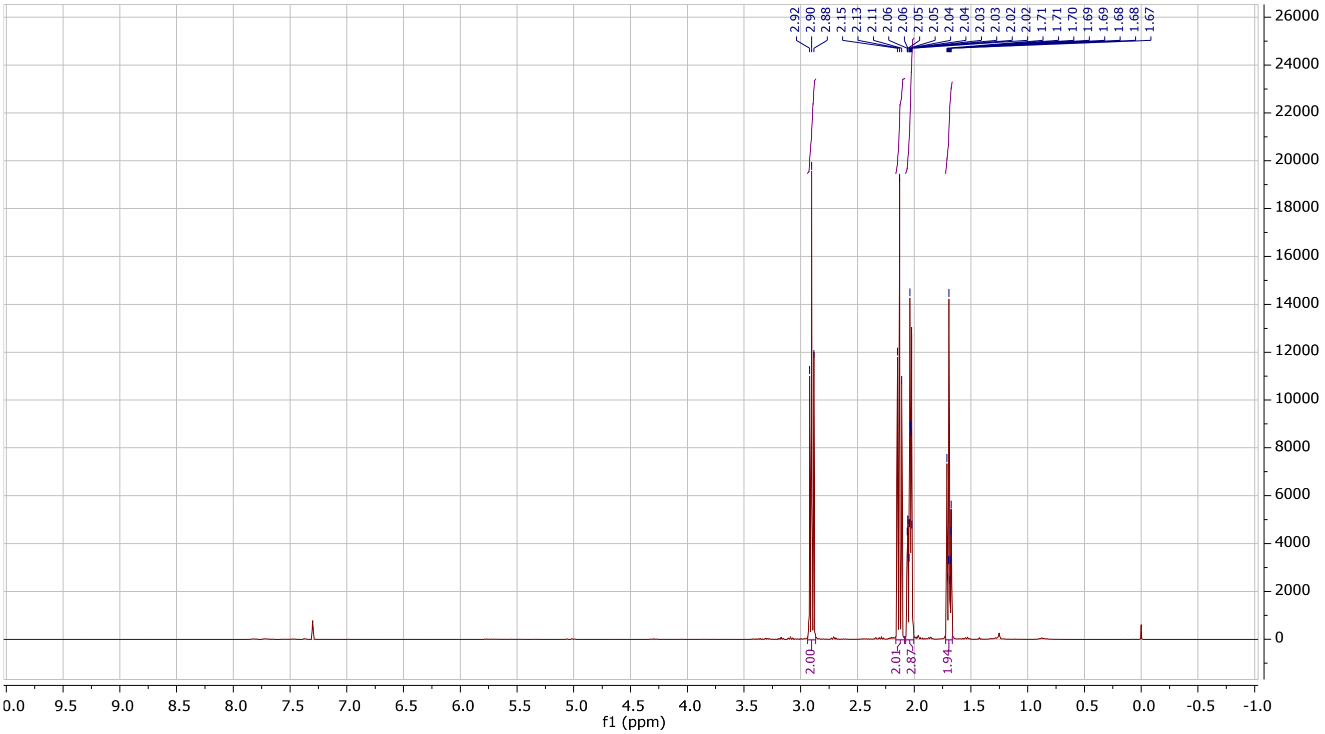

2.92
2.90
2.88
2.15
2.13
2.11
2.06
2.06
2.05
2.05
2.04
2.04
2.03
2.03
2.02
2.02
1.71
1.71
1.70
1.69
1.69
1.68
1.68
1.67
2.00
2.01
2.87
1.94
f1 (ppm)

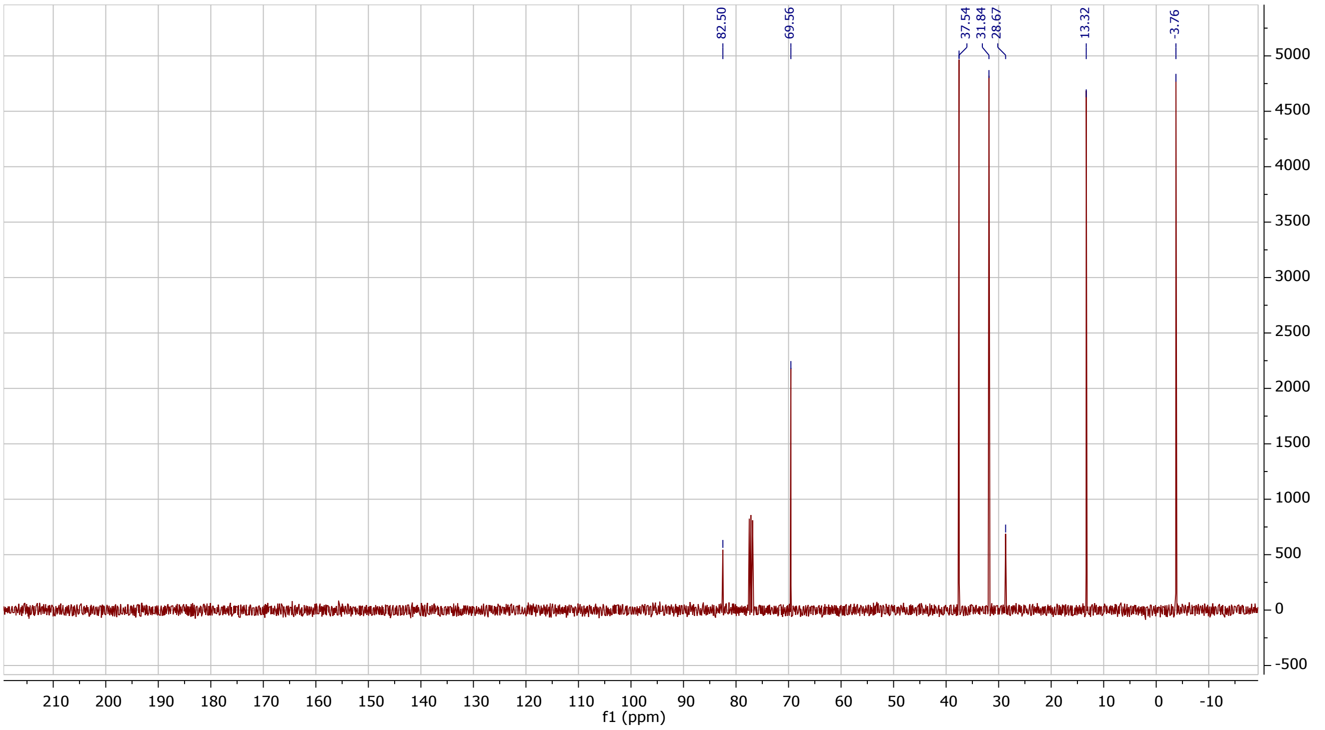

82.50
69.56
37.54
31.84
28.67
13.32
-3.76
f1 (ppm)

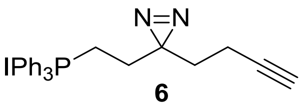

6

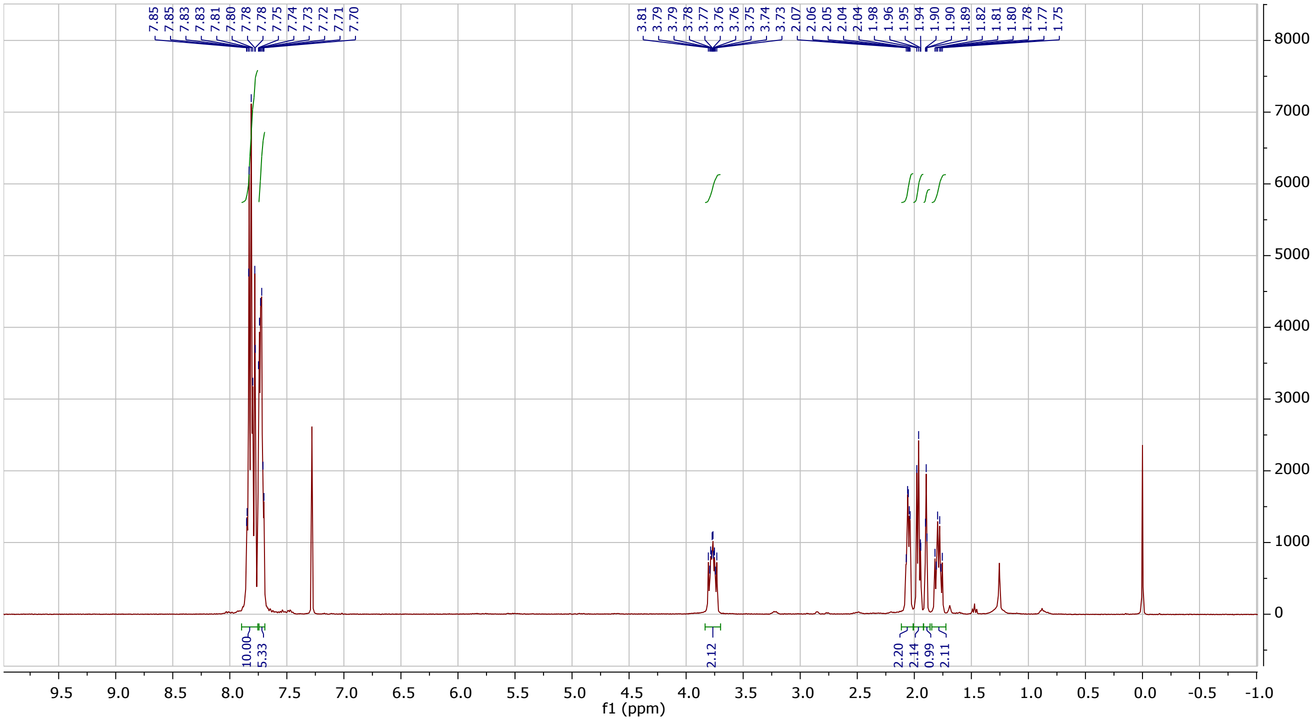

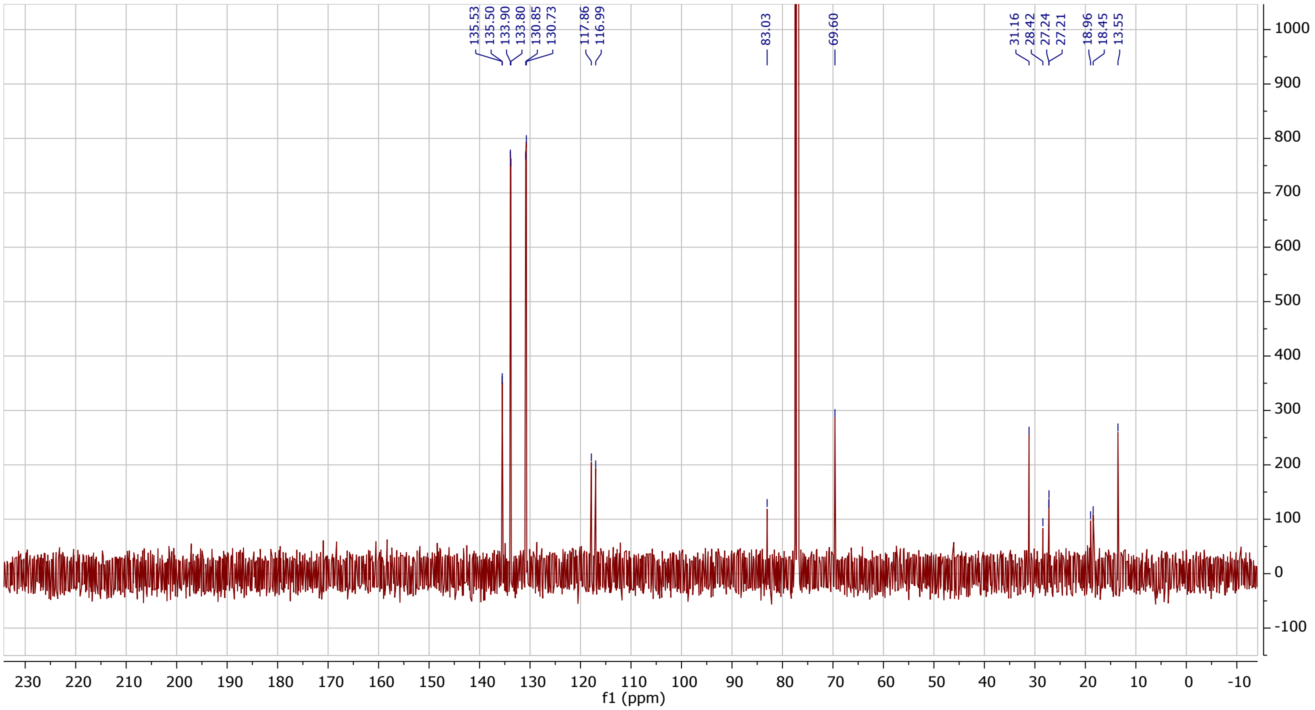

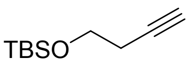
TBSO

12

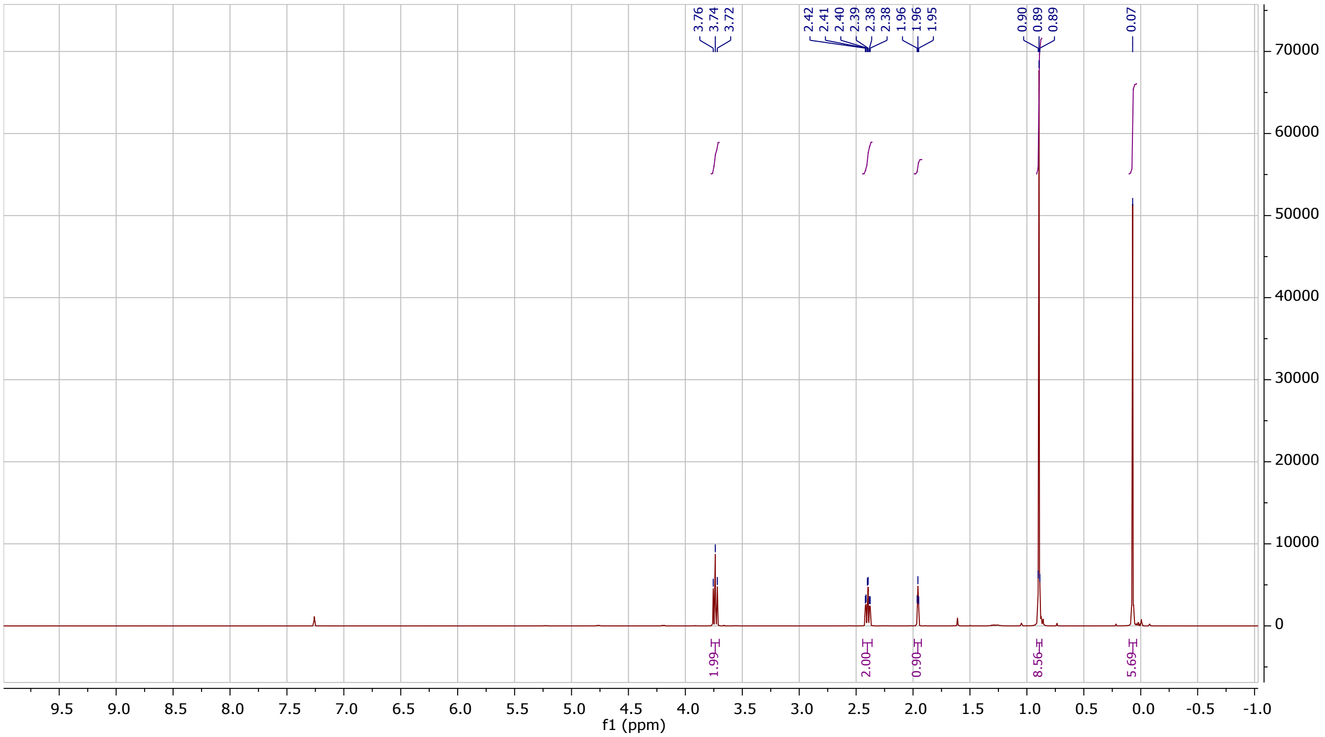
3.76
3.74
3.72
2.42
2.41
2.40
2.39
2.38
2.38
1.96
1.96
1.95
0.90
0.89
0.89
0.07
1.99
2.00
0.90
8.56
5.69
f1 (ppm)

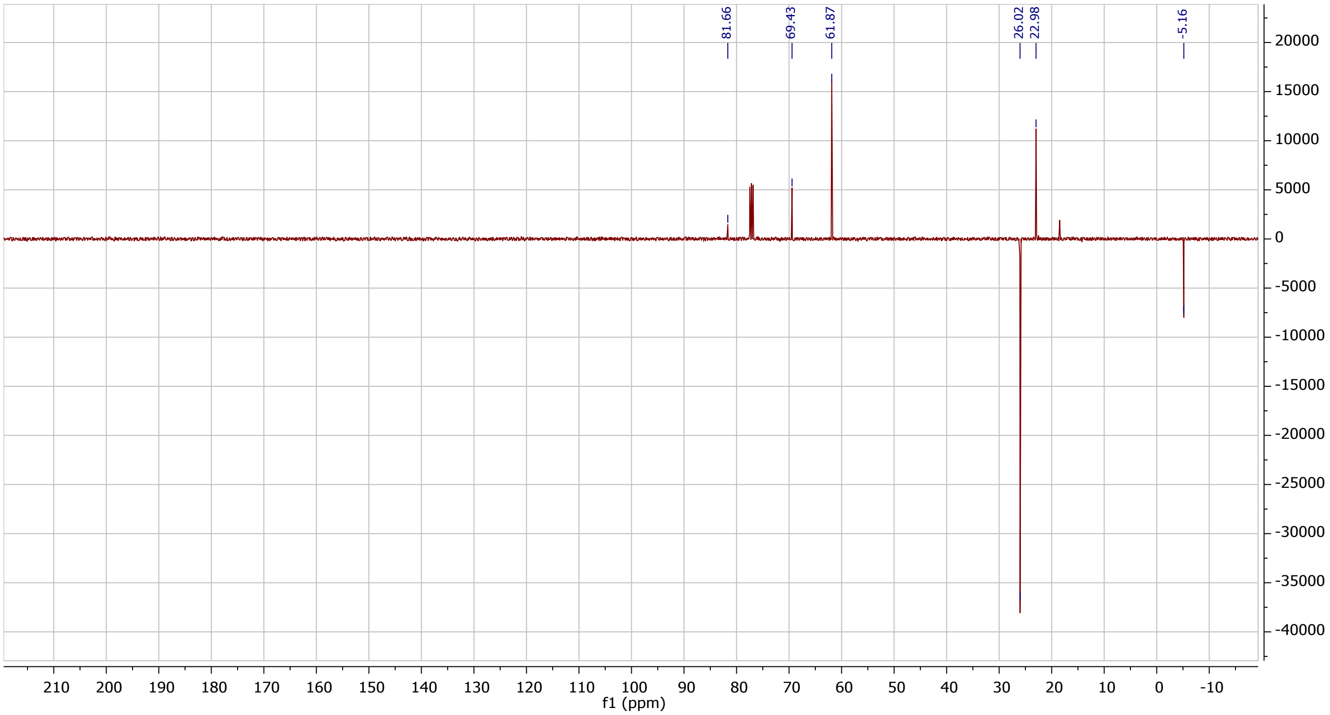
81.66
69.43
61.87
26.02
22.98
-5.16
f1 (ppm)

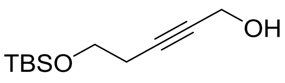

13

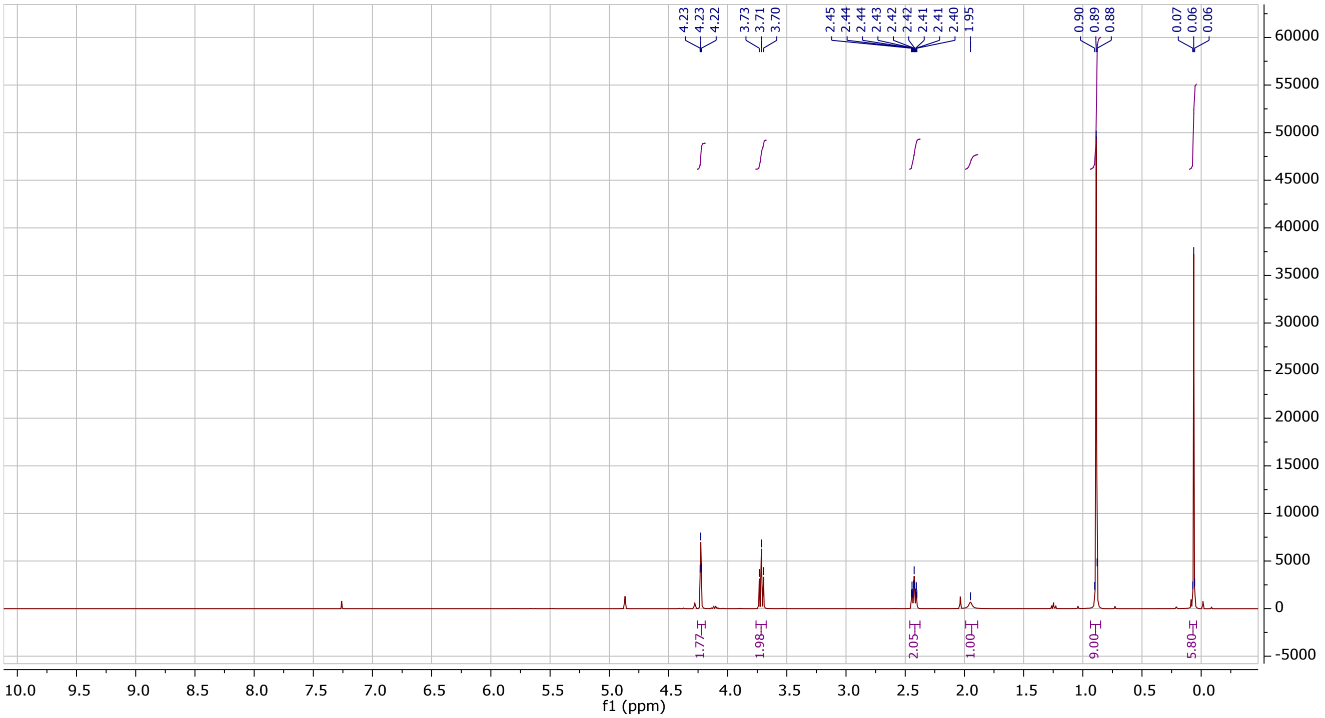

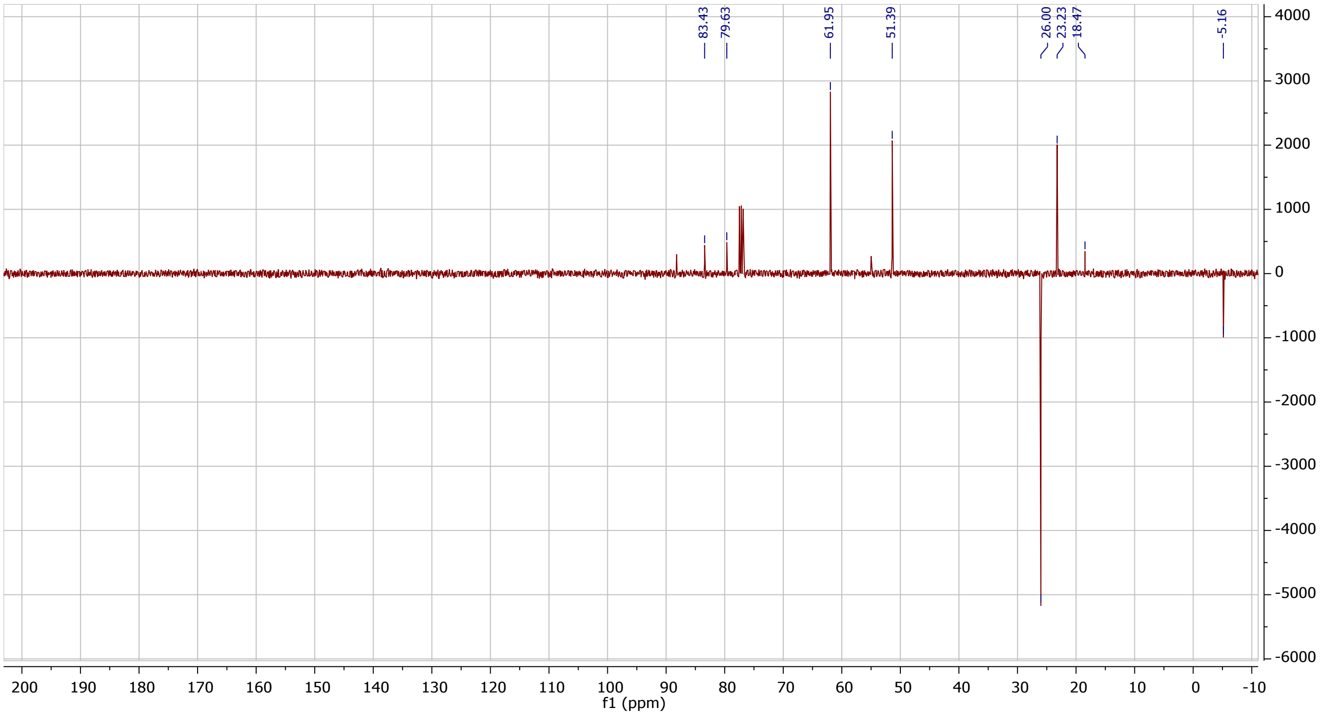

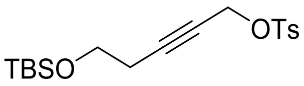

OTs
TBSO

14

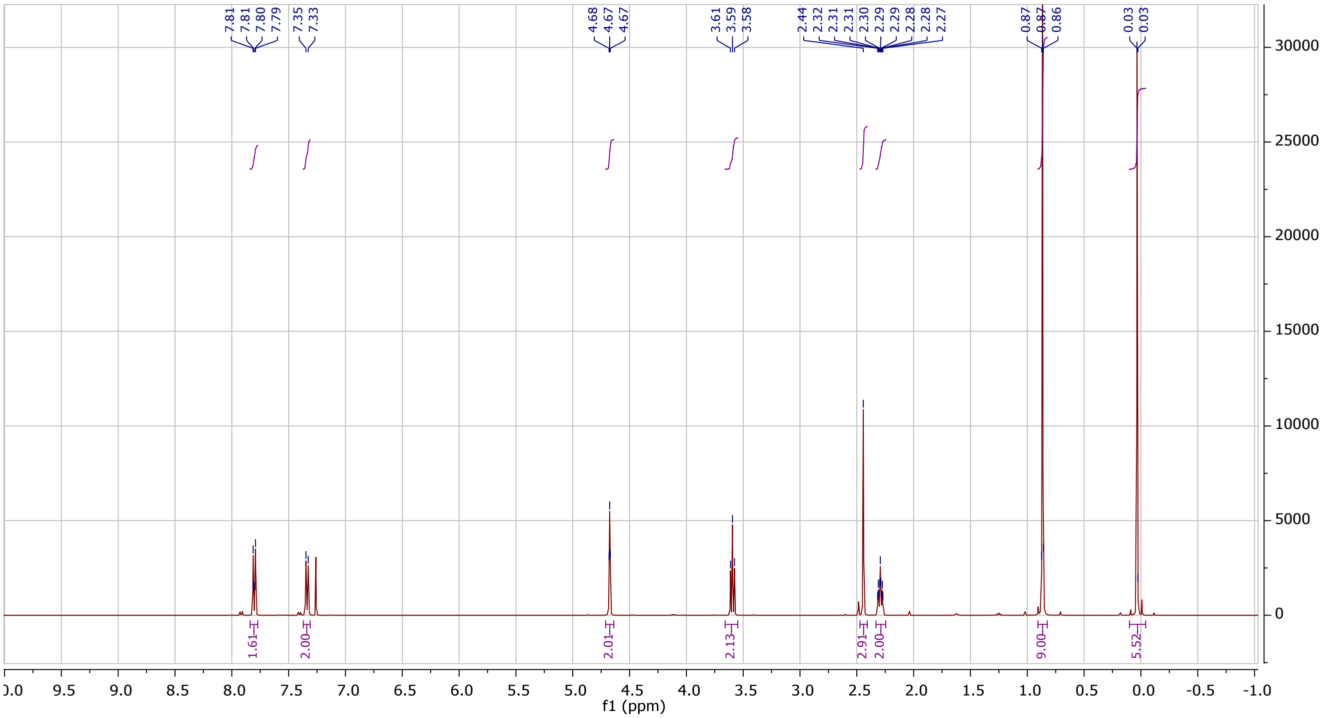

7.81
7.81
7.80
7.79
7.35
7.33
4.68
4.67
4.67
3.61
3.59
3.58
2.44
2.32
2.31
2.31
2.30
2.29
2.29
2.28
2.27
0.87
0.87
0.86
0.03
0.03
1.61
2.00
2.01
2.13
2.91
2.00
9.00
5.52
f1 (ppm)

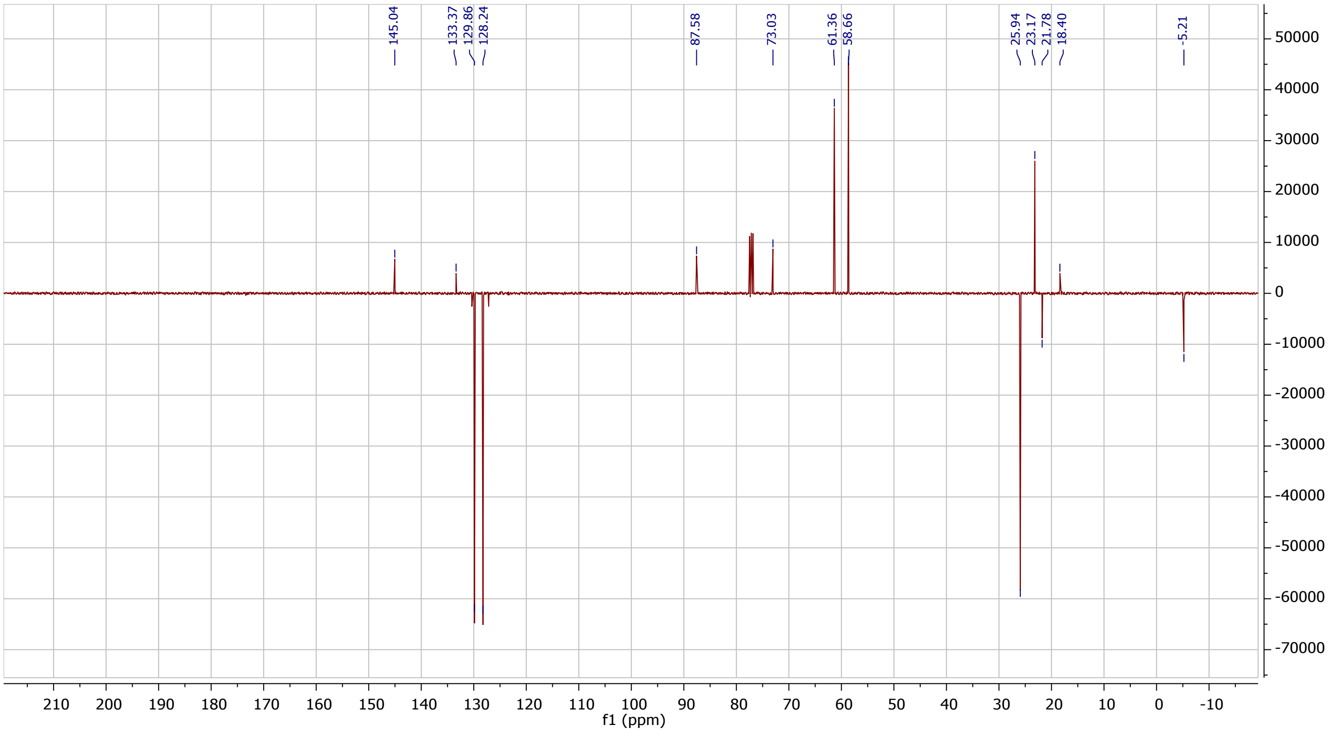

145.04
133.37
129.86
128.24
87.58
73.03
61.36
58.66
25.94
23.17
21.78
18.40
-5.21
f1 (ppm)

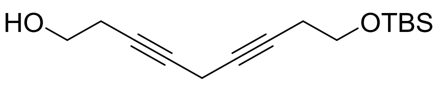
HO
OTBS

15

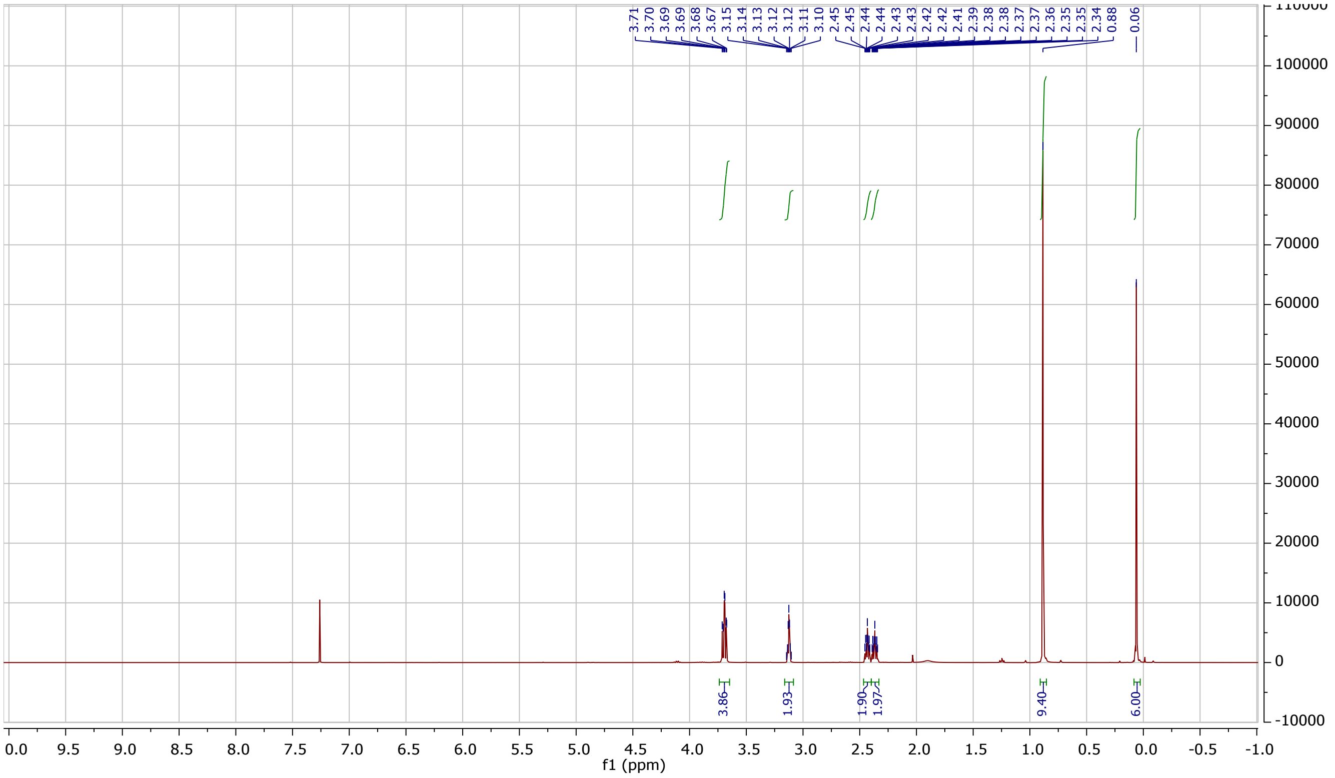
3.71 3.70 3.69 3.69 3.68 3.67 3.15 3.14 3.13 3.12 3.12 3.11 3.10 2.45 2.45 2.44 2.44 2.43 2.43 2.42 2.42 2.41 2.39 2.38 2.38 2.37 2.37 2.36 2.35 2.35 2.34 0.88 0.06
3.86
1.93
1.90
1.97
9.40
6.00
f1 (ppm)

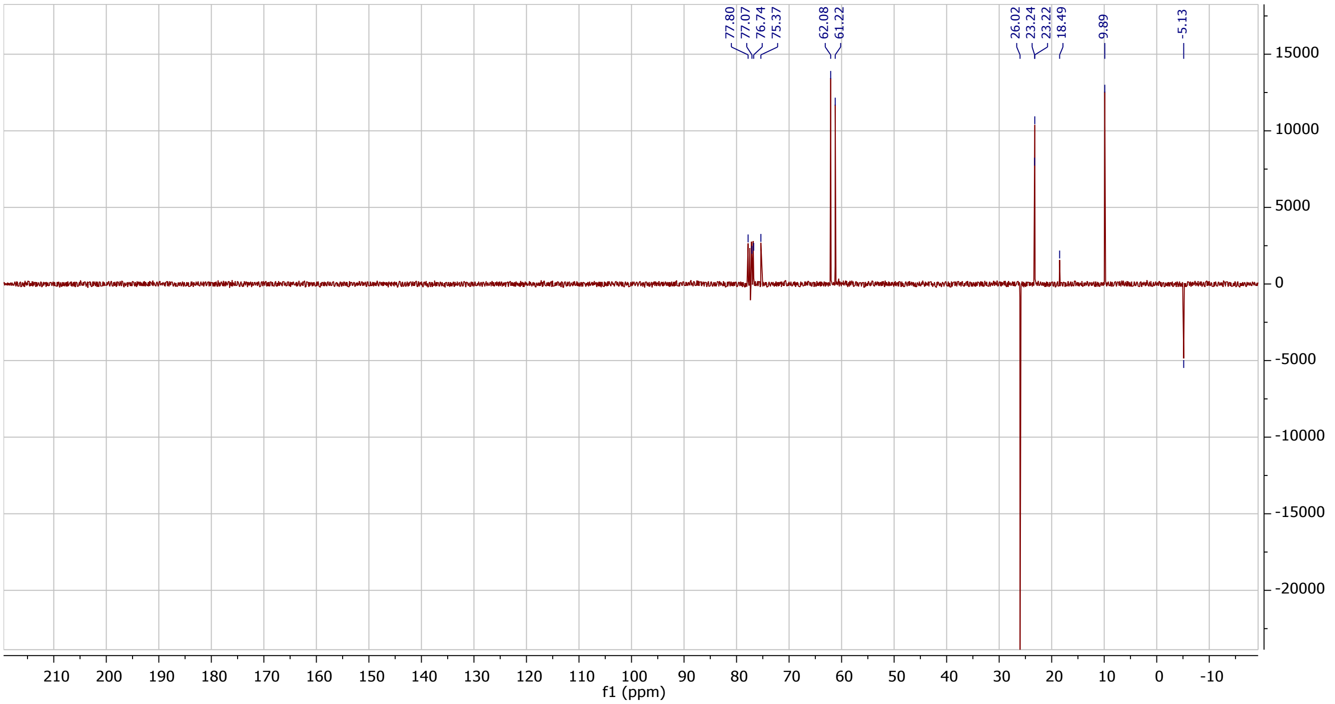
77.80 77.07 76.74 75.37 62.08 61.22 26.02 23.24 23.22 18.49 9.89 -5.13
f1 (ppm)

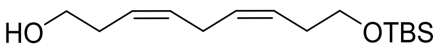
HO
OTBS

9

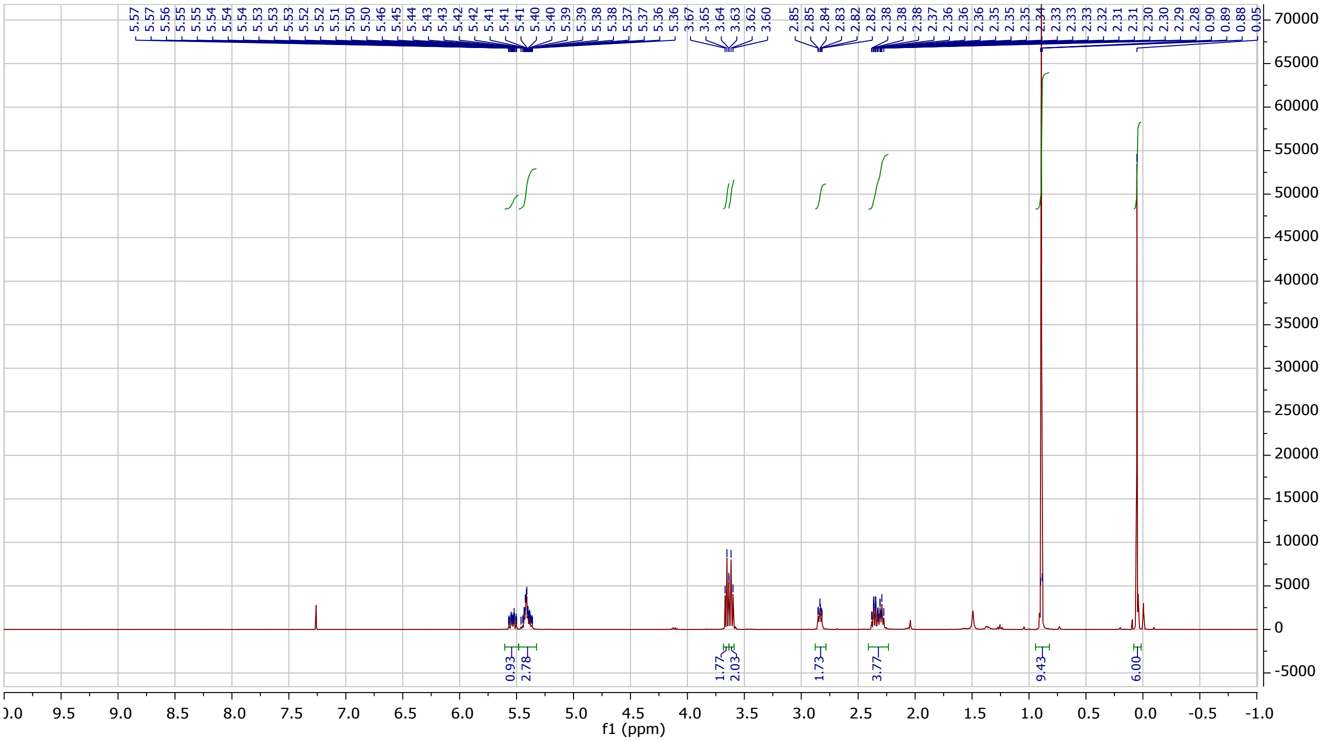
0.93
2.78
1.77
2.03
1.73
3.77
9.43
6.00
f1 (ppm)

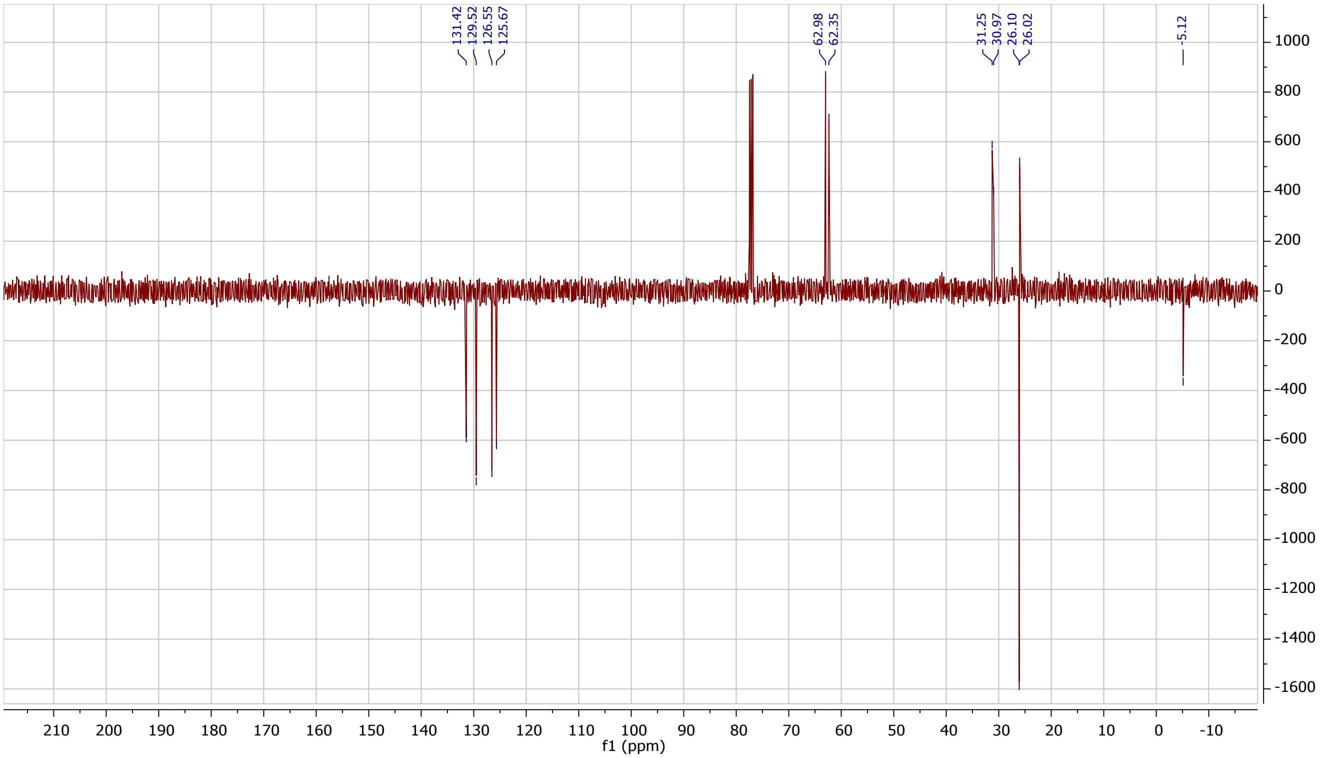
131.42
129.52
126.55
125.67
62.98
62.35
31.25
30.97
26.10
26.02
-5.12
f1 (ppm)

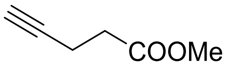
COOMe

17

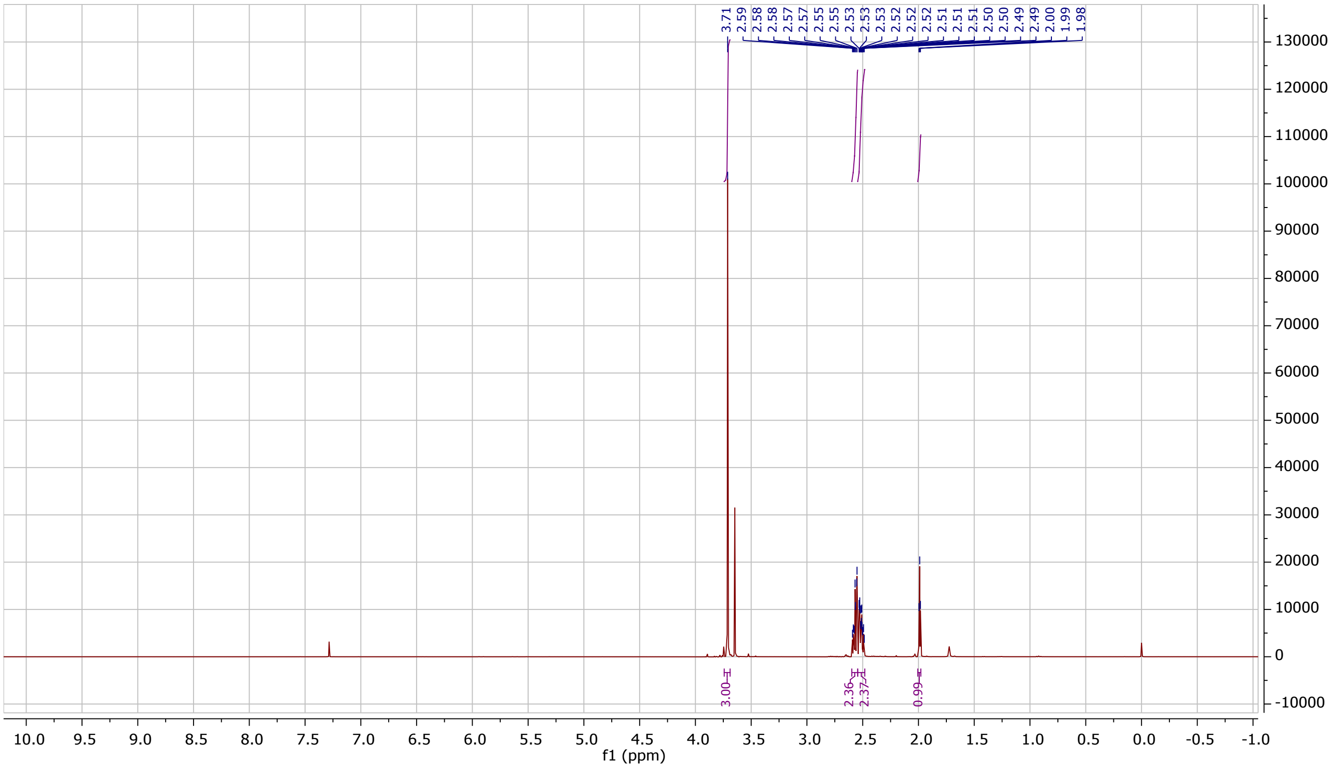
3.71
2.59 2.58 2.58 2.57 2.57 2.55 2.55 2.53 2.53 2.53 2.53 2.52 2.52 2.52 2.51 2.51 2.51 2.50 2.50 2.49 2.00 1.99 1.98
3.00
2.36
2.37
0.99
f1 (ppm)

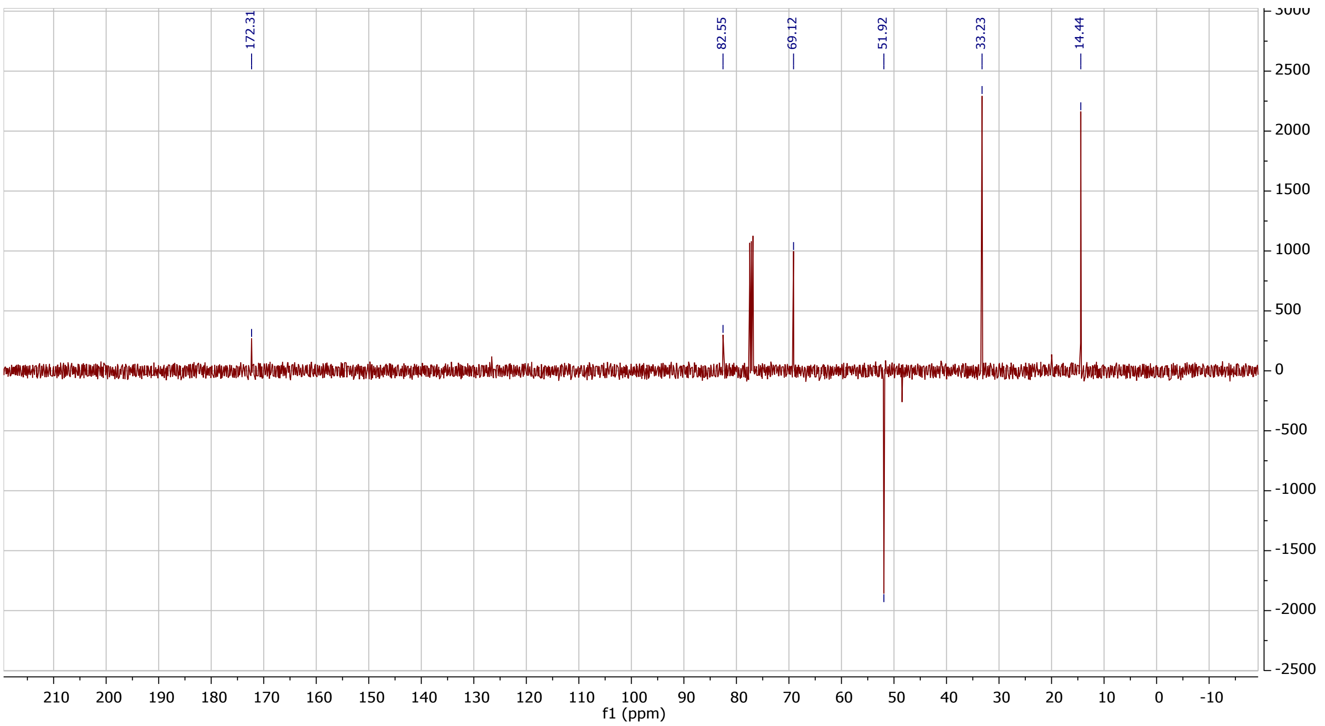
172.31
82.55
69.12
51.92
33.23
14.44
f1 (ppm)

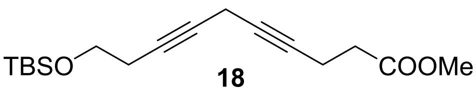

TBSO
COOMe

18

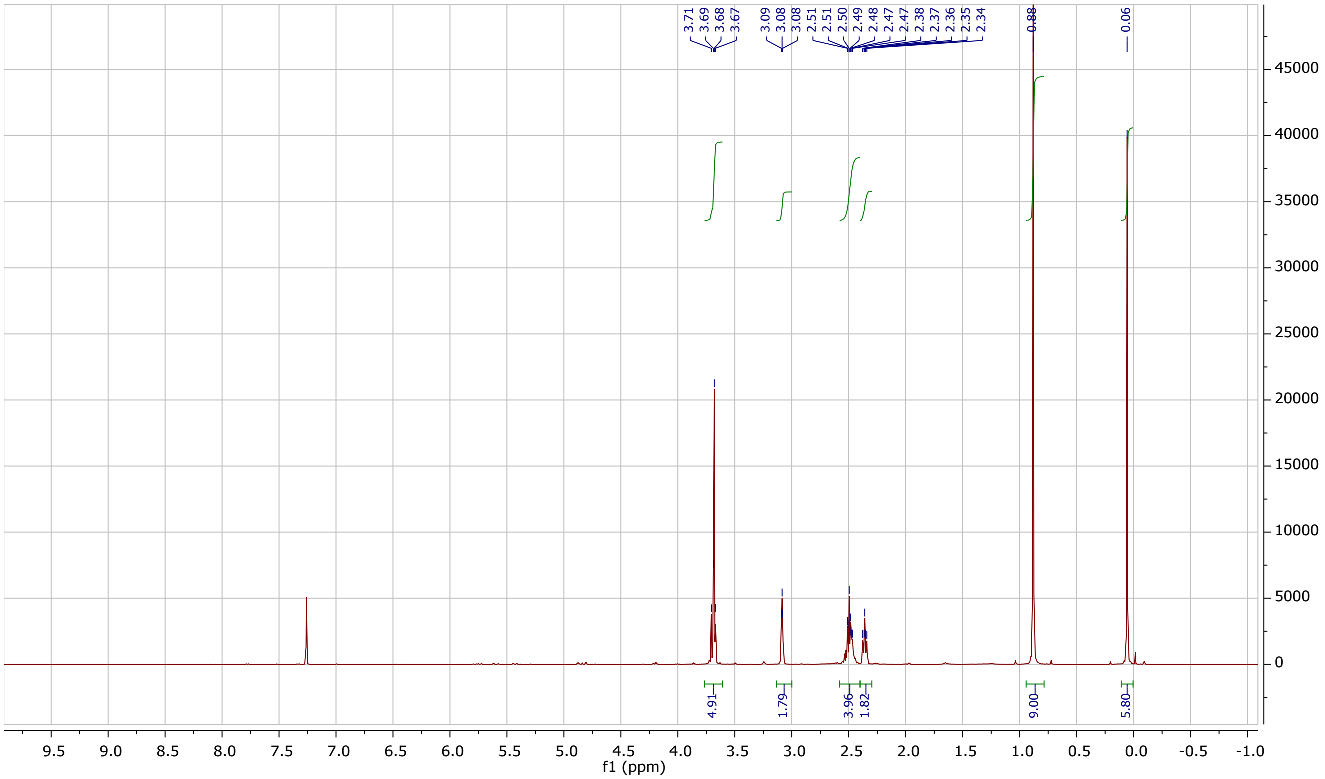

3.71
3.69
3.68
3.67
3.09
3.08
3.08
2.51
2.51
2.50
2.49
2.48
2.47
2.47
2.38
2.37
2.36
2.35
2.34
0.88
0.06
4.91
1.79
3.96
1.82
9.00
5.80
f1 (ppm)

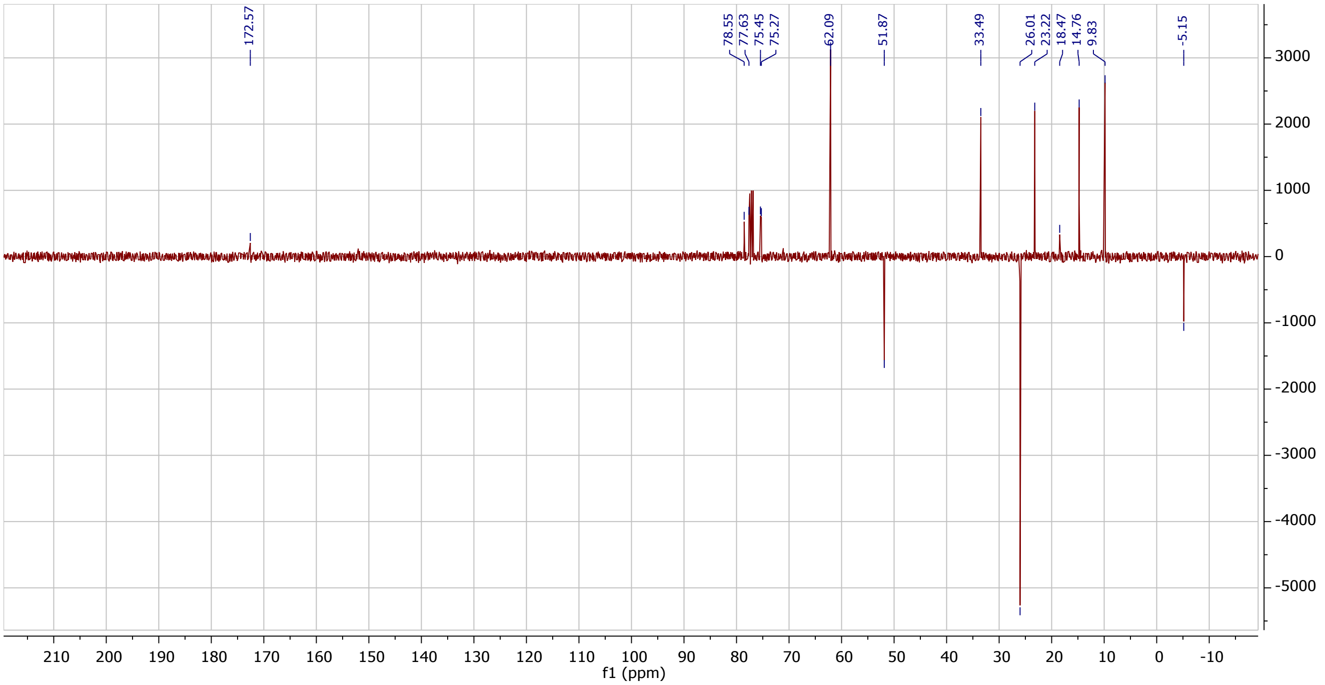

172.57
78.55
77.63
75.45
75.27
62.09
51.87
33.49
26.01
23.22
18.47
14.76
9.83
-5.15
f1 (ppm)

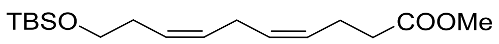
TBSO
COOMe

19

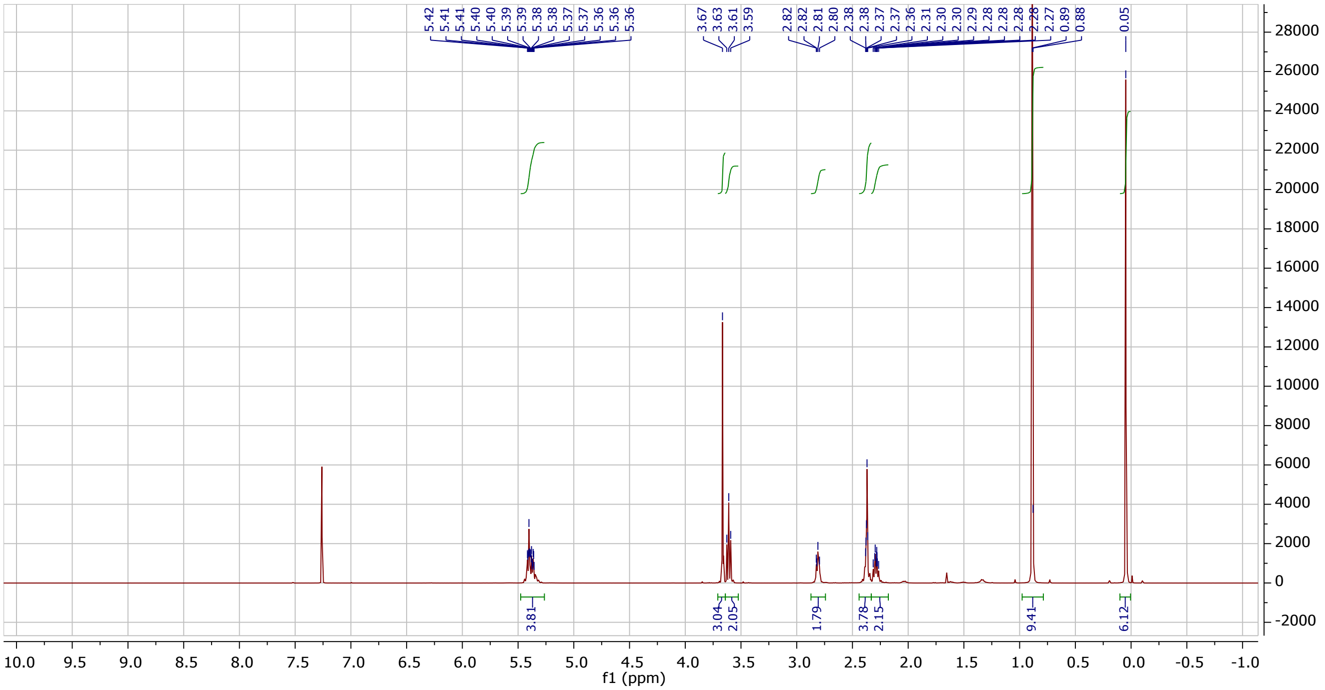
5.42 5.41 5.41 5.40 5.40 5.39 5.39 5.38 5.38 5.37 5.37 5.36 5.36 5.36
3.67 3.63 3.61 3.59
2.82 2.82 2.81 2.80 2.38 2.38 2.38 2.37 2.37 2.36 2.31 2.30 2.30 2.29 2.28 2.28 2.28 2.28 2.27 0.89 0.88
0.05
3.81 3.04 2.05 1.79 3.78 2.15 9.41 6.12
f1 (ppm)

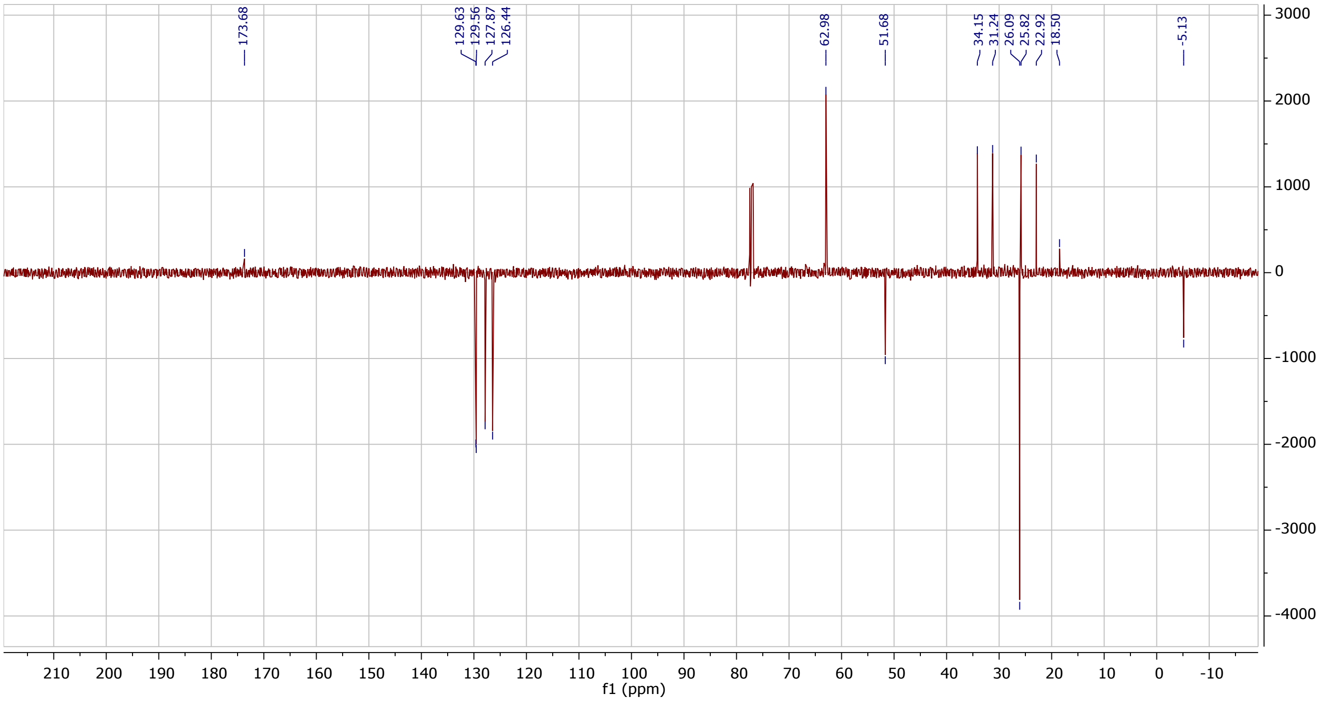
173.68
129.63 129.56 127.87 126.44
62.98
51.68
34.15 31.24 26.09 25.82 22.92 18.50
-5.13
f1 (ppm)

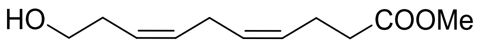
HO
COOMe

20

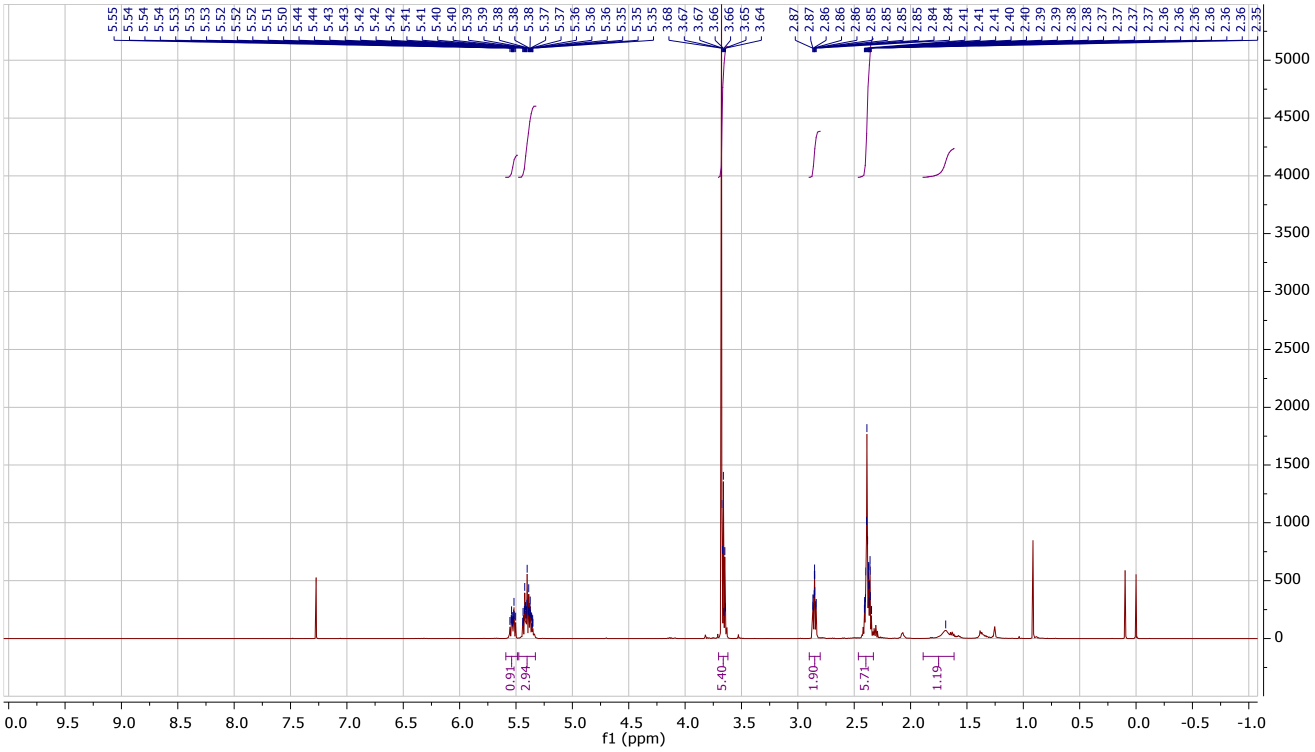
0.91
2.94
5.40
1.90
5.71
1.19
f1 (ppm)

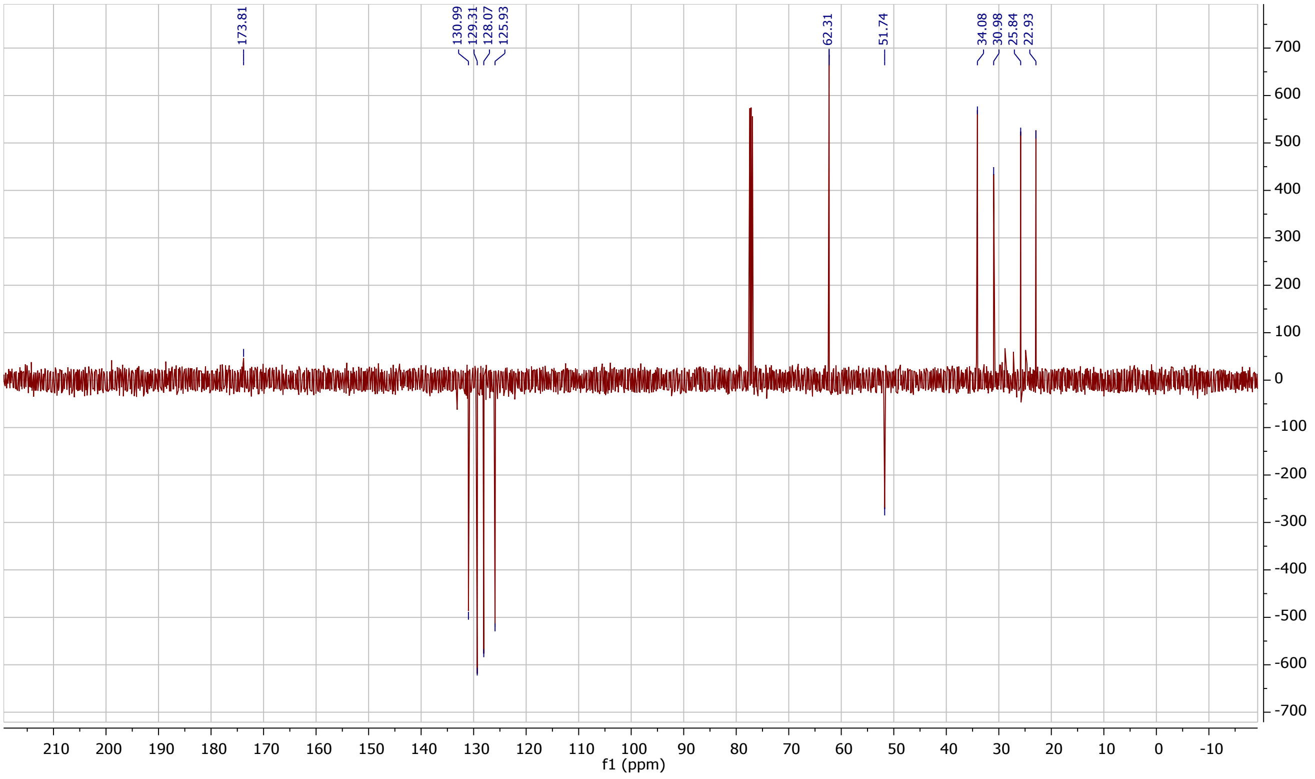
173.81
130.99
129.31
128.07
125.93
62.31
51.74
34.08
30.98
25.84
22.93
f1 (ppm)

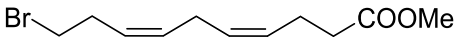
Br
COOMe

21

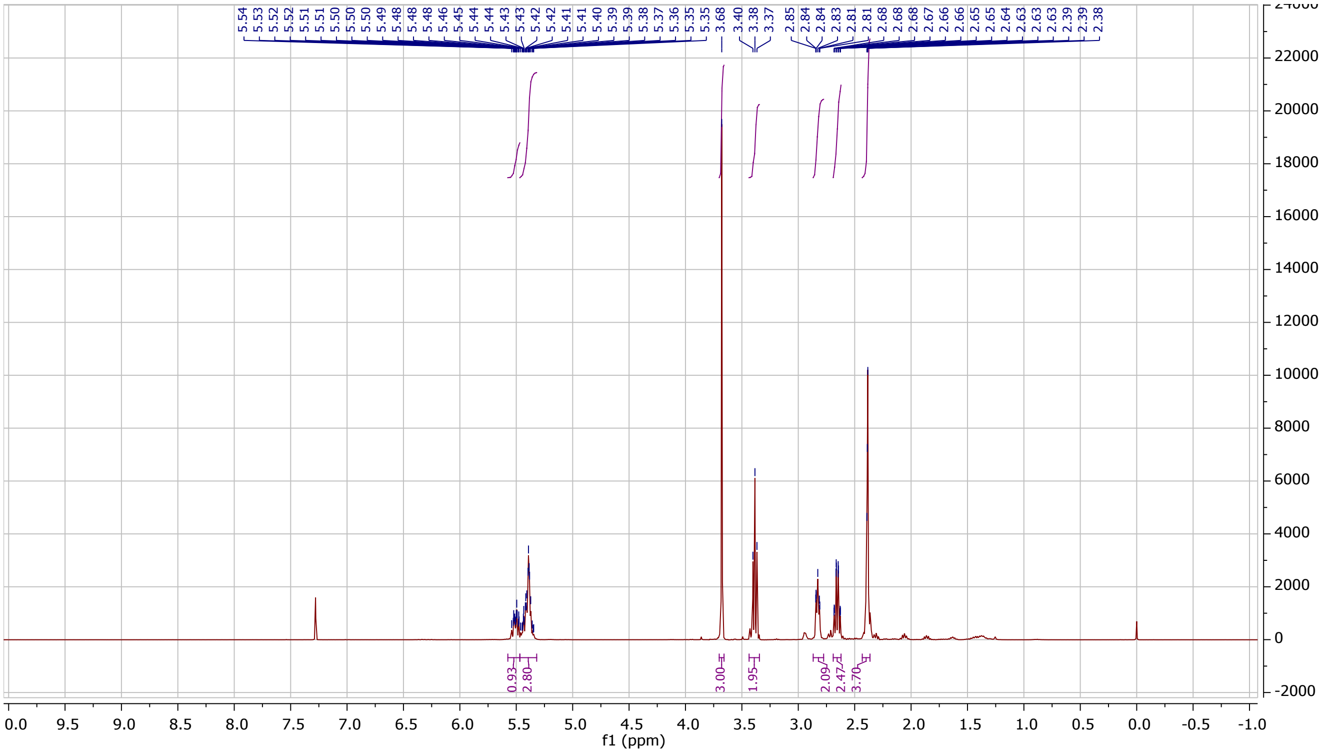
5.54 5.53 5.52 5.52 5.51 5.51 5.50 5.50 5.50 5.49 5.48 5.48 5.48 5.46 5.45 5.44 5.44 5.44 5.43 5.43 5.42 5.42 5.41 5.41 5.40 5.39 5.39 5.38 5.37 5.36 5.35 5.35
3.68
3.40 3.38 3.37
2.85 2.84 2.84 2.83 2.83 2.81 2.81 2.68 2.68 2.68 2.67 2.66 2.66 2.65 2.65 2.64 2.63 2.63 2.63 2.39 2.39 2.38
0.93
2.80
3.00
1.95
2.09
2.47
3.70
f1 (ppm)

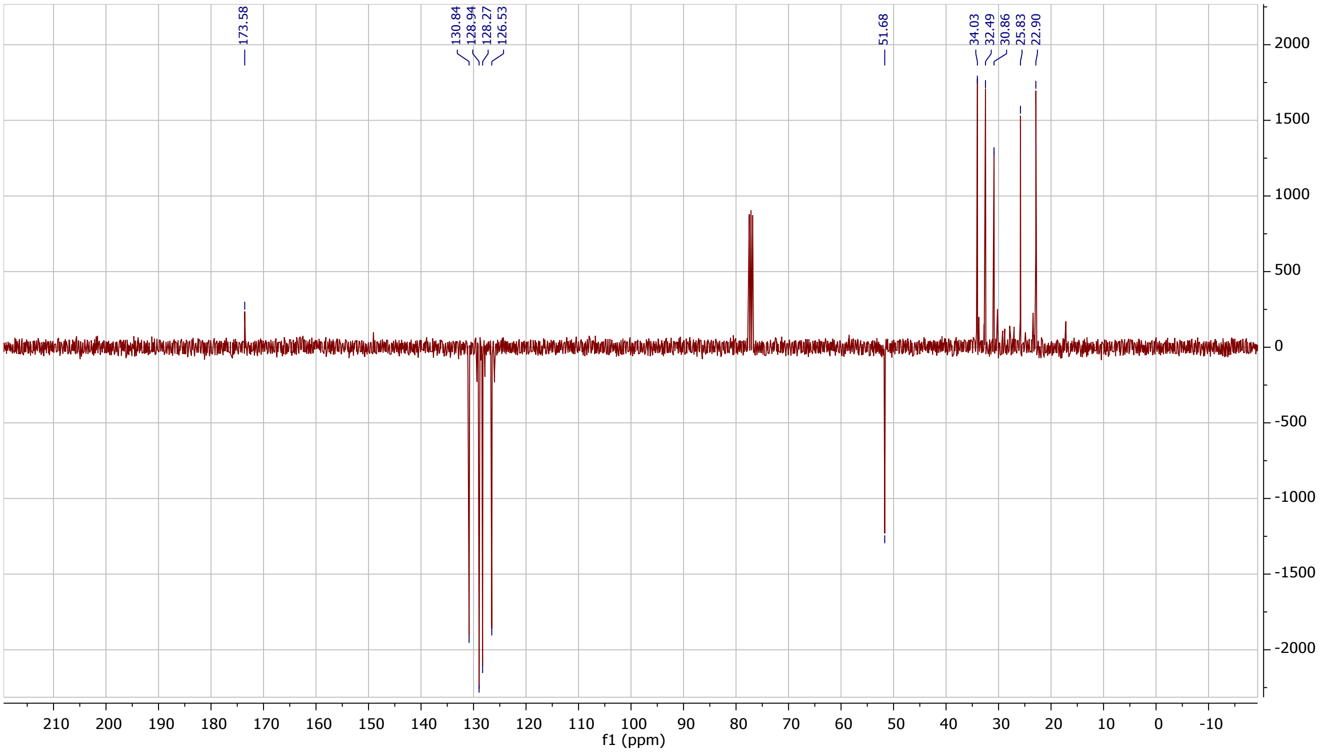
173.58
130.84
128.94
128.27
126.53
51.68
34.03
32.49
30.86
25.83
22.90
f1 (ppm)

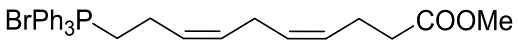
BrPh3P
COOMe

8

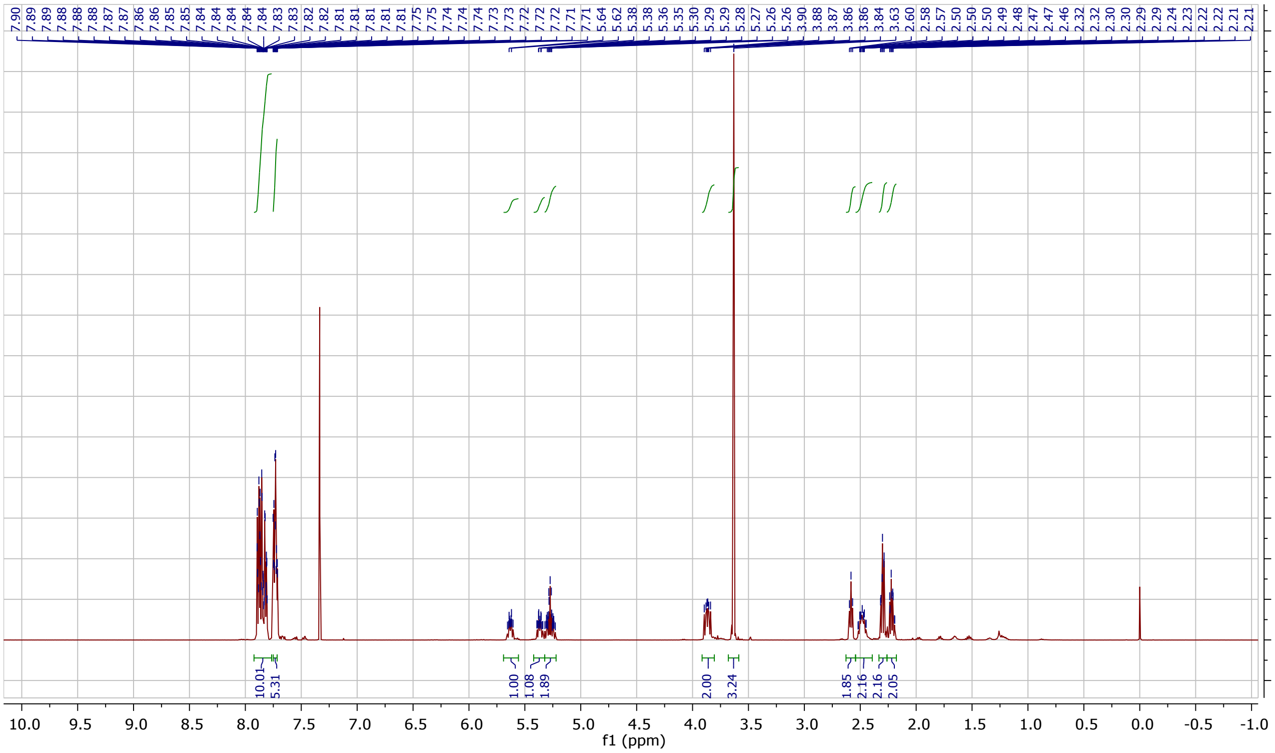
7.90 7.89 7.89 7.88 7.88 7.88 7.87 7.87 7.86 7.85 7.85 7.84 7.84 7.84 7.84 7.83 7.83 7.82 7.82 7.81 7.81 7.81 7.81 7.81 7.75 7.75 7.74 7.74 7.74 7.73 7.73 7.72 7.72 7.72 7.71 7.71 5.64 5.62 5.38 5.38 5.36 5.35 5.30 5.29 5.29 5.28 5.27 5.26 5.26 3.90 3.88 3.87 3.86 3.86 3.84 3.63 2.60 2.58 2.57 2.50 2.50 2.50 2.49 2.48 2.47 2.47 2.46 2.32 2.32 2.30 2.30 2.29 2.29 2.24 2.23 2.22 2.22 2.21 2.21
10.01
5.31
1.00
1.08
1.89
2.00
3.24
1.85
2.16
2.16
2.05
f1 (ppm)

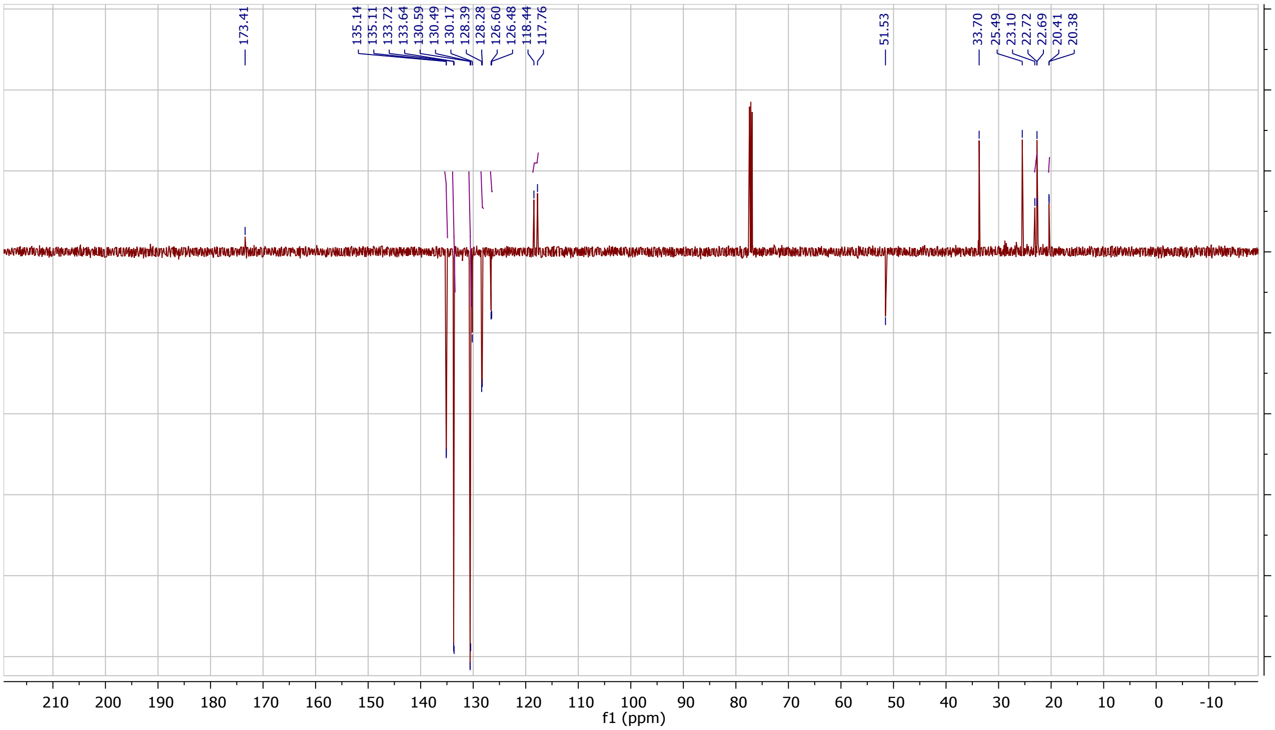
173.41
135.14 135.11 133.72 133.64 130.59 130.49 130.17 128.39 128.28 126.60 126.48 118.44 117.76
51.53
33.70 25.49 23.10 22.72 22.69 20.41 20.38
f1 (ppm)

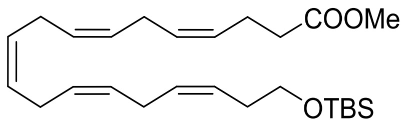

24

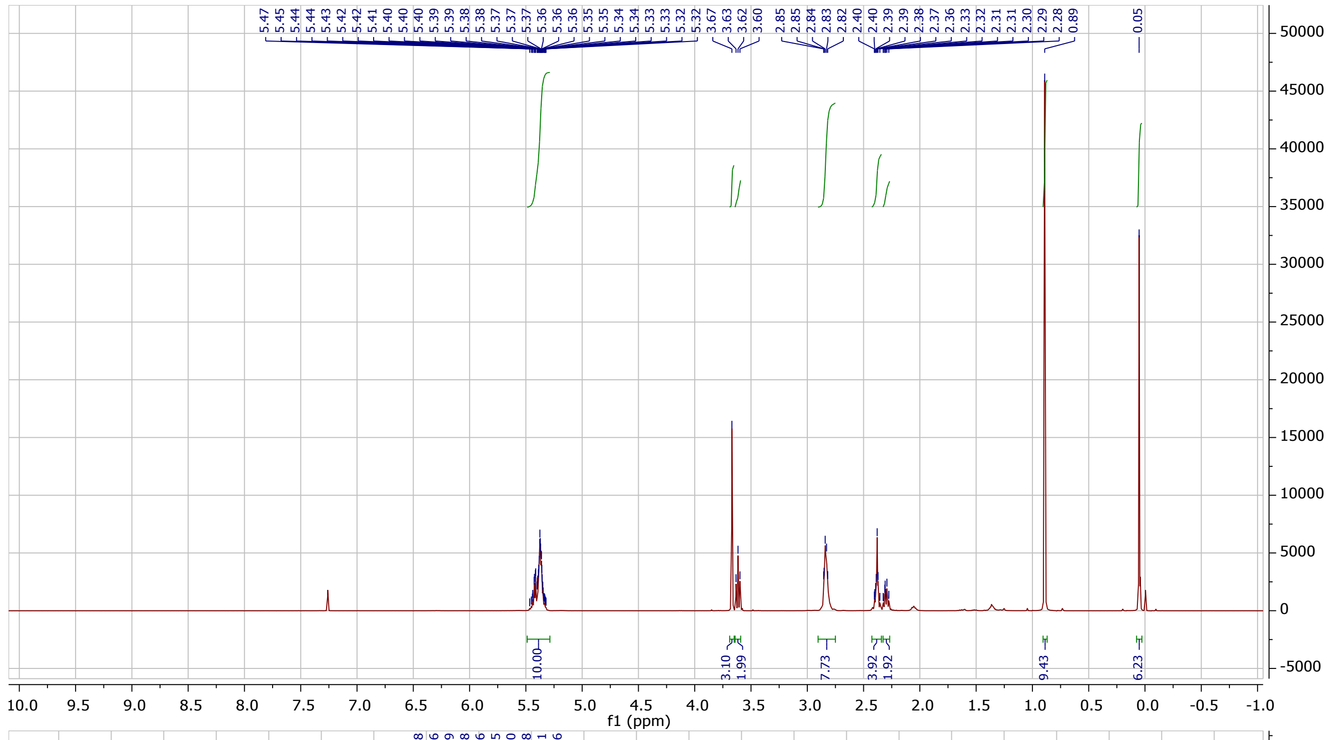

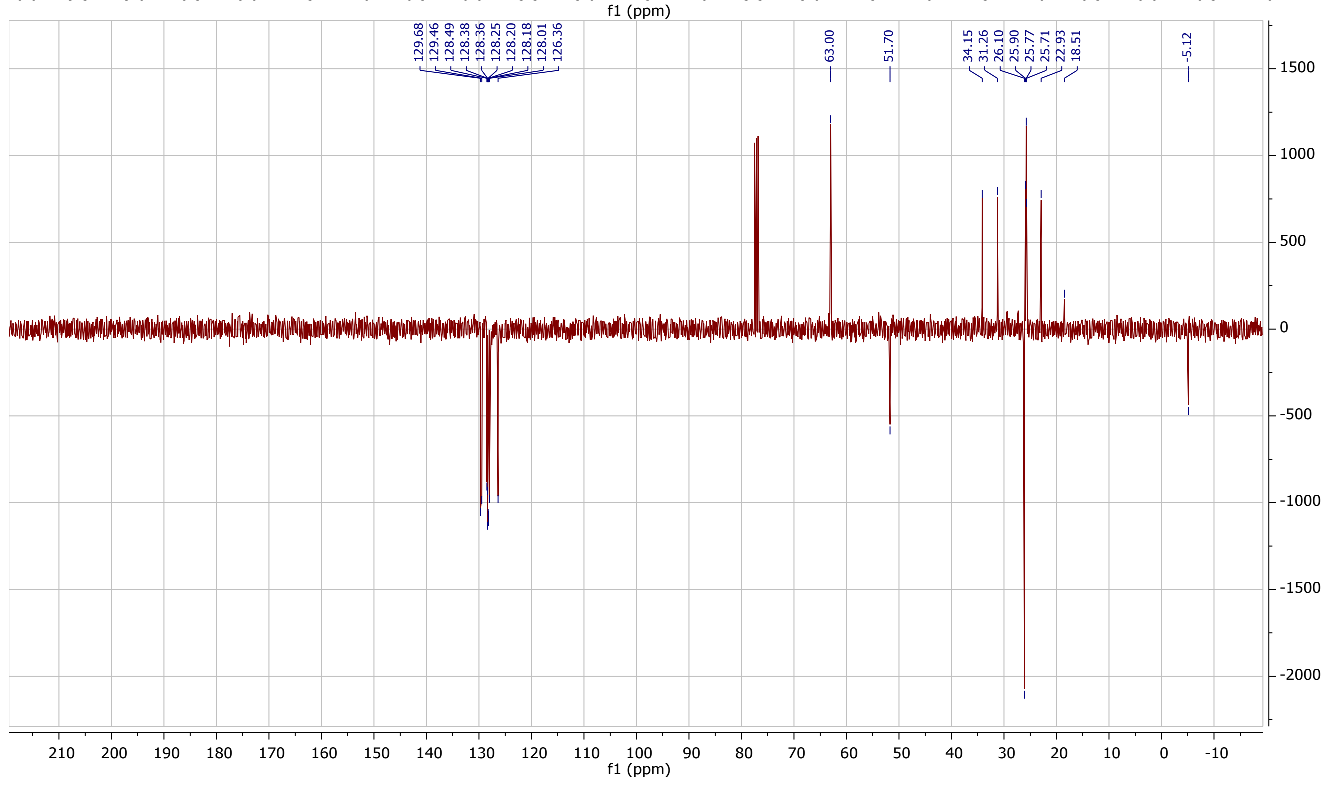

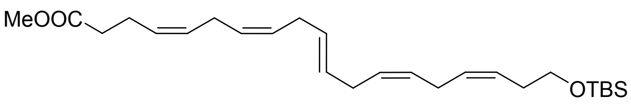

24 - trans byproduct

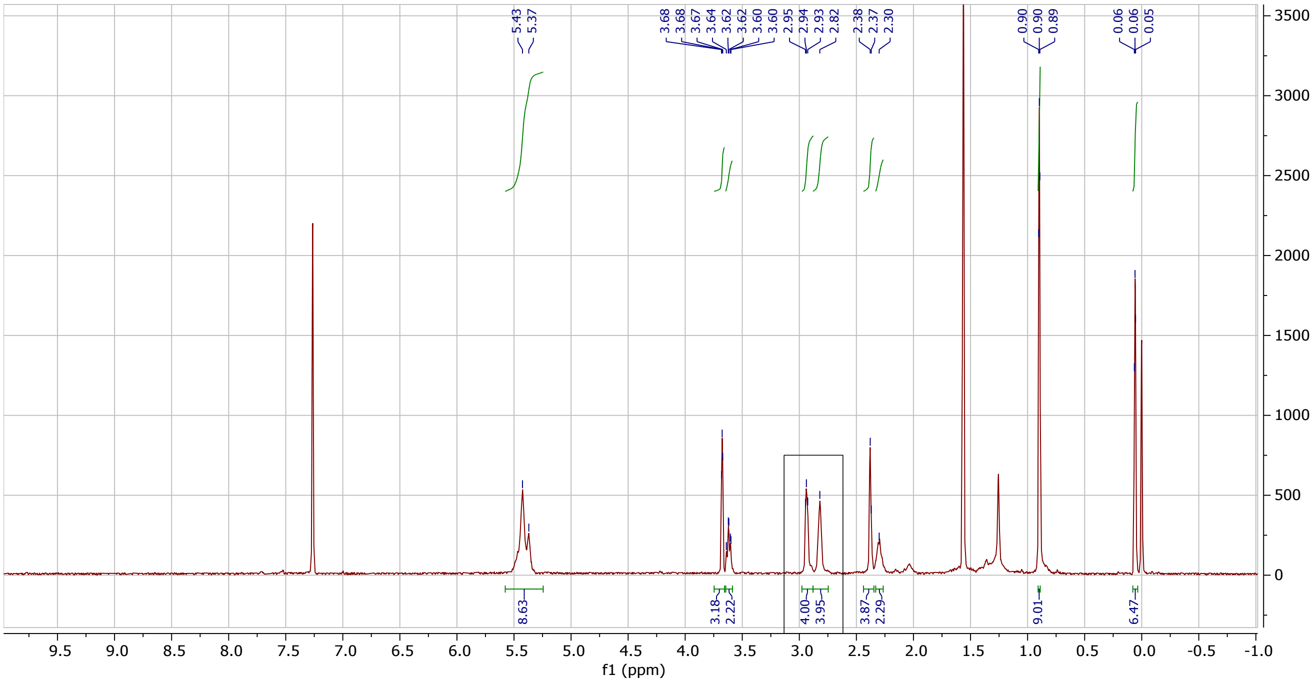

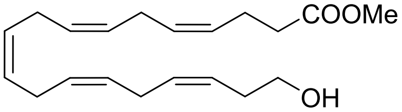
COOMe
OH

7

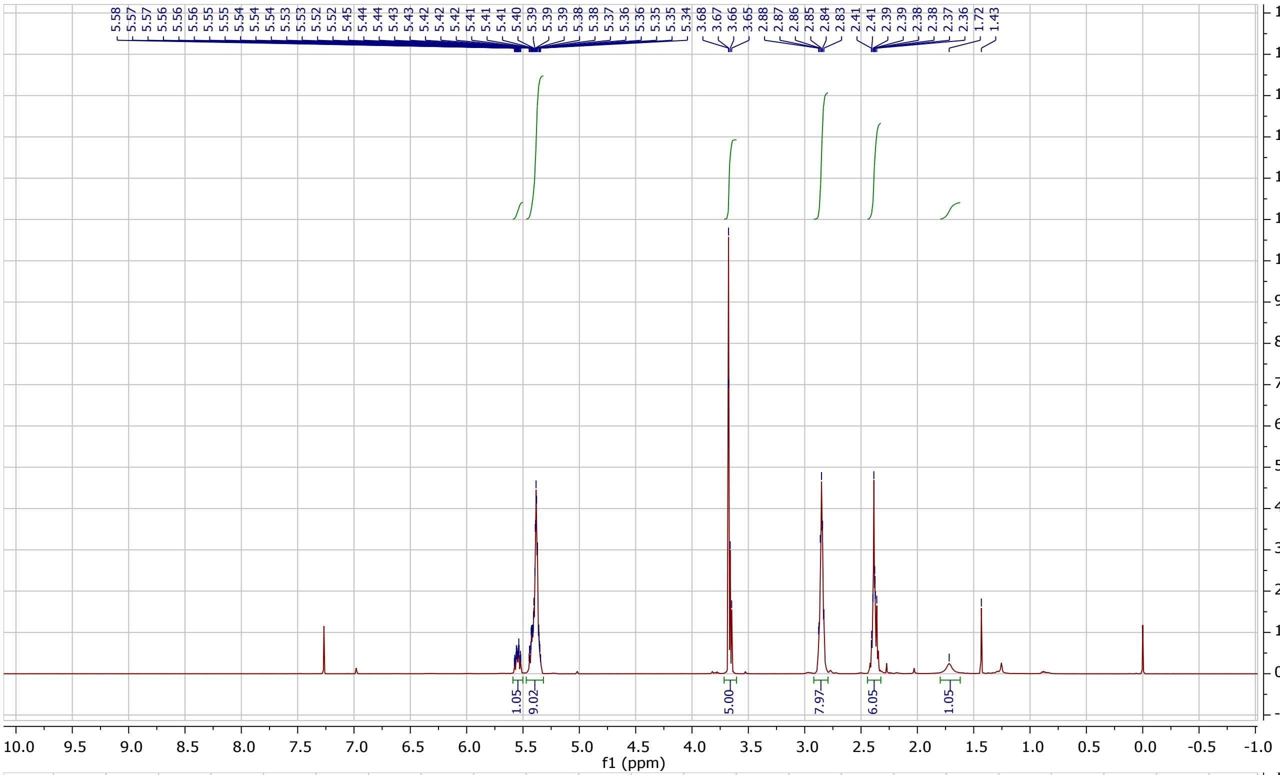
5.58 5.57 5.57 5.56 5.56 5.56 5.55 5.55 5.54 5.54 5.54 5.53 5.53 5.52 5.52 5.45 5.44 5.44 5.43 5.43 5.42 5.42 5.42 5.41 5.41 5.41 5.40 5.39 5.39 5.39 5.38 5.38 5.37 5.36 5.35 5.35 5.34 3.68 3.67 3.66 3.65 2.88 2.87 2.86 2.85 2.84 2.83 2.41 2.41 2.39 2.39 2.38 2.38 2.37 2.36 1.72 1.43
1.05 9.02 5.00 7.97 6.05 1.05
f1 (ppm)

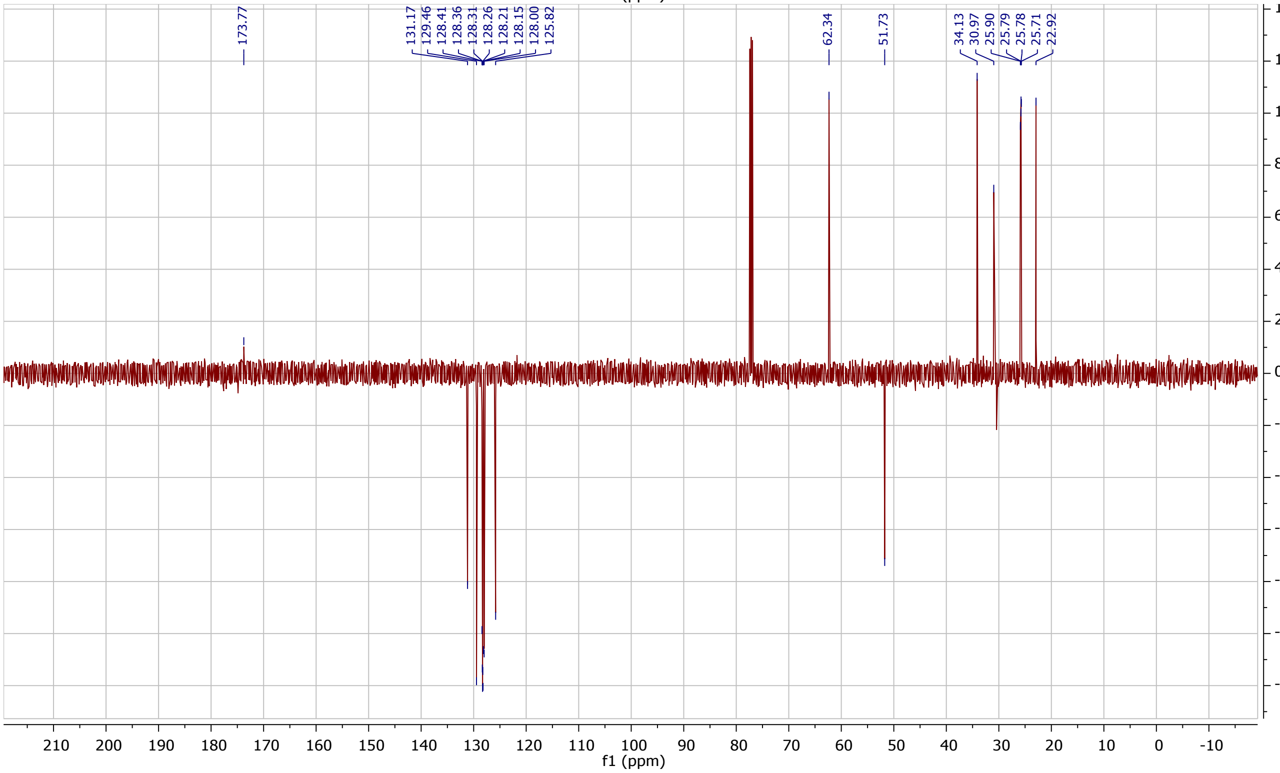
173.77
131.17 129.46 128.41 128.36 128.31 128.26 128.21 128.15 128.00 125.82
62.34
51.73
34.13 30.97 25.90 25.79 25.78 25.71 22.92
f1 (ppm)

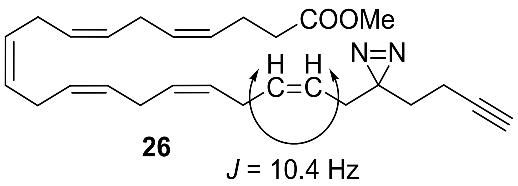
COOMe
N=N
H H
C=C
J = 10.4 Hz

26

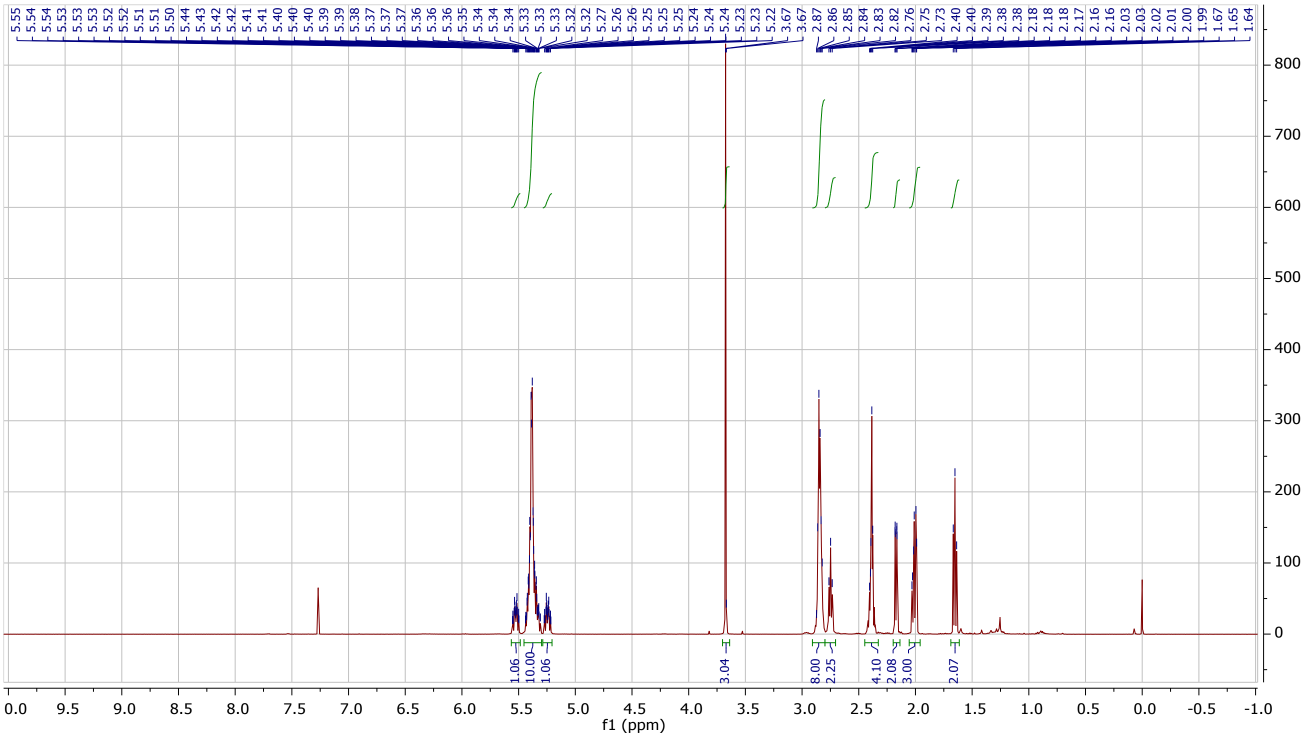
5.55 5.54 5.54 5.53 5.53 5.53 5.52 5.52 5.51 5.51 5.50 5.44 5.43 5.42 5.42 5.41 5.41 5.40 5.40 5.40 5.39 5.39 5.38 5.37 5.37 5.37 5.36 5.36 5.36 5.35 5.34 5.34 5.34 5.33 5.33 5.33 5.33 5.32 5.32 5.27 5.26 5.26 5.25 5.25 5.25 5.24 5.24 5.24 5.23 5.23 5.22 5.22 3.67 2.87 2.86 2.85 2.84 2.83 2.82 2.76 2.75 2.73 2.40 2.40 2.39 2.38 2.38 2.18 2.18 2.18 2.17 2.16 2.16 2.03 2.02 2.01 2.00 1.99 1.67 1.65 1.64
1.06
10.00
1.06
3.04
8.00
2.25
4.10
2.08
3.00
2.07
f1 (ppm)

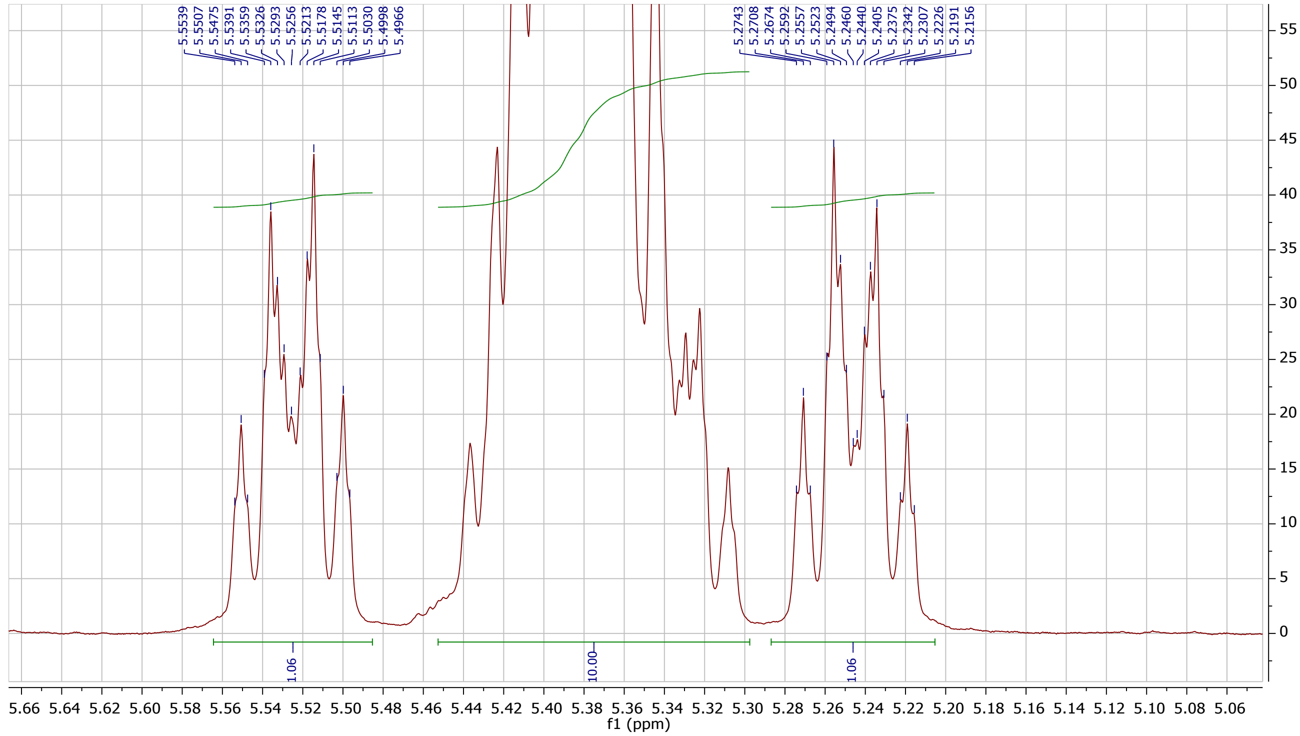
f1 (ppm)

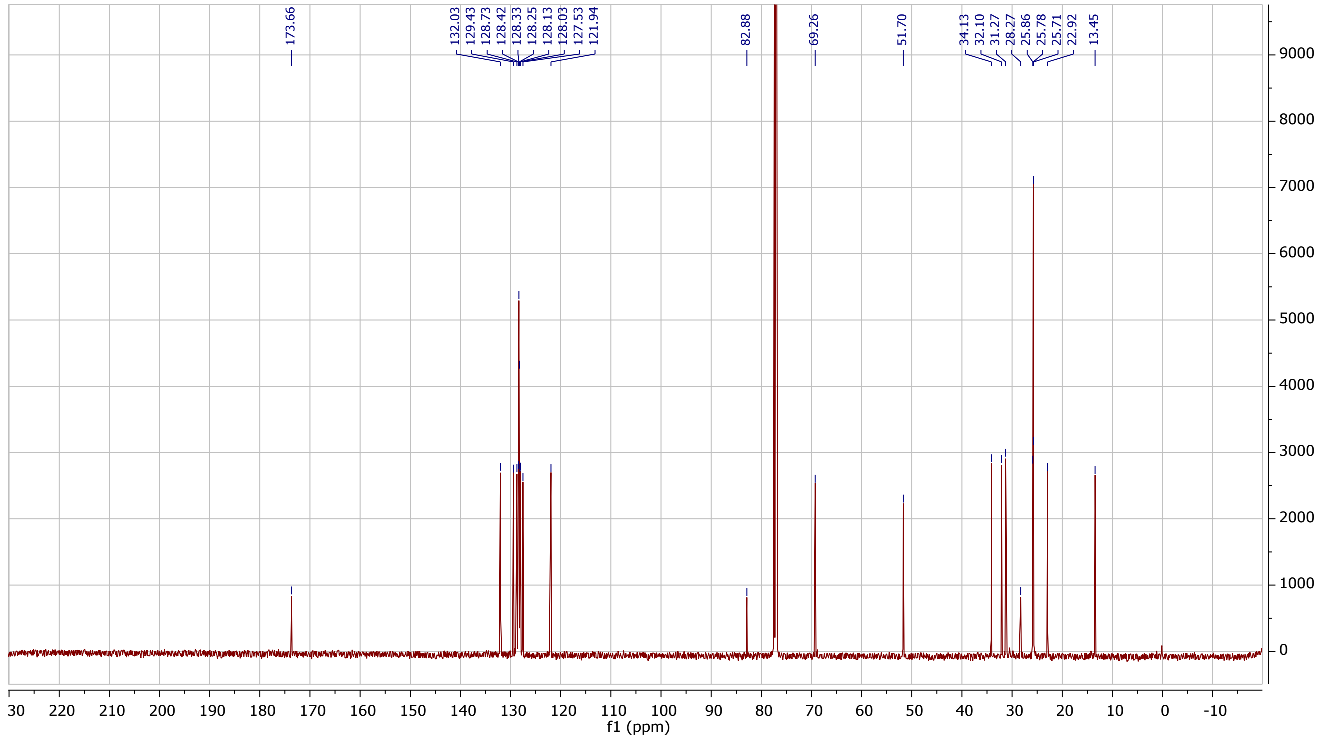
f1 (ppm)

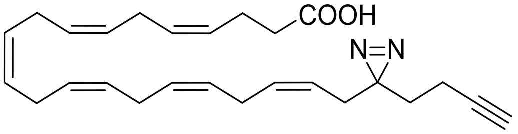
COOH
N=N

pac-DHA (4)

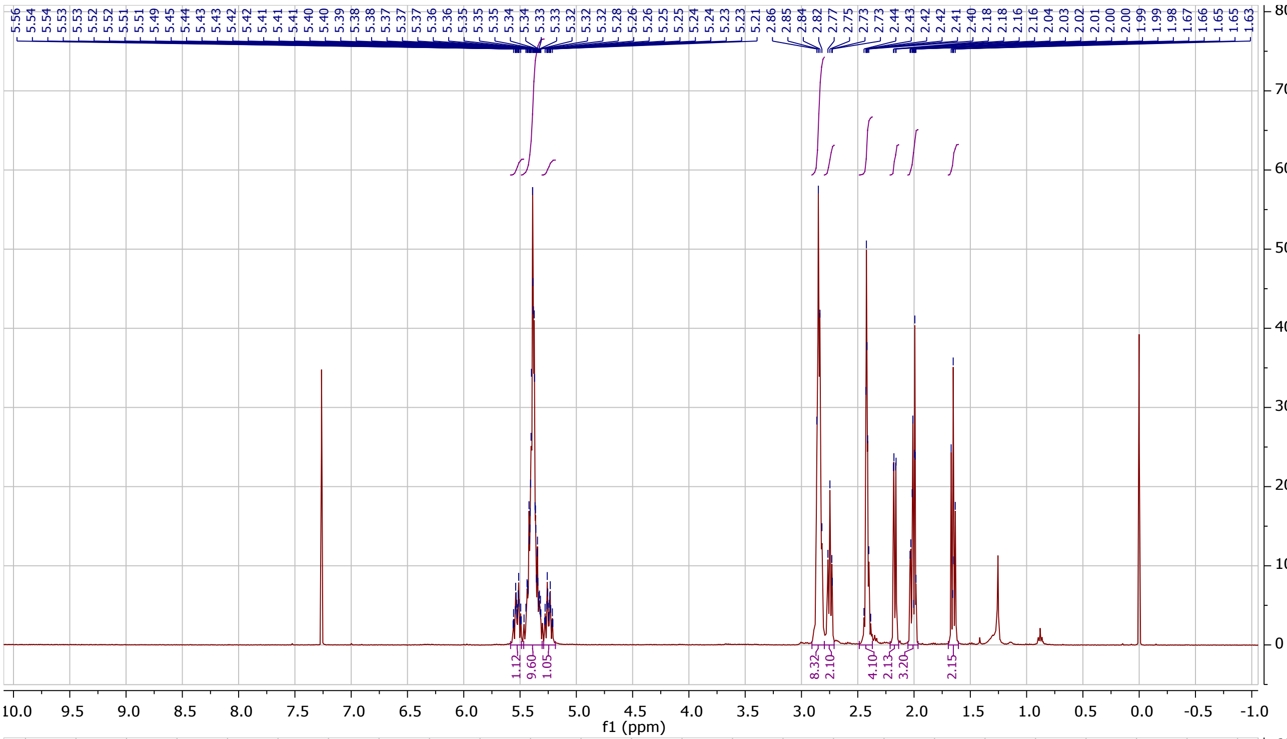
5.56 5.54 5.54 5.53 5.53 5.52 5.52 5.51 5.51 5.49 5.45 5.44 5.43 5.43 5.42 5.42 5.41 5.41 5.41 5.40 5.39 5.38 5.38 5.37 5.37 5.37 5.36 5.35 5.35 5.35 5.34 5.34 5.33 5.33 5.32 5.32 5.32 5.28 5.26 5.26 5.25 5.25 5.24 5.24 5.23 5.23 5.21 2.86 2.84 2.82 2.77 2.75 2.73 2.73 2.44 2.43 2.42 2.42 2.41 2.40 2.18 2.16 2.16 2.04 2.03 2.02 2.01 2.00 1.99 1.98 1.67 1.66 1.65 1.63
1.12 9.60 1.05 8.32 2.10 4.10 2.13 3.20 2.15
f1 (ppm)

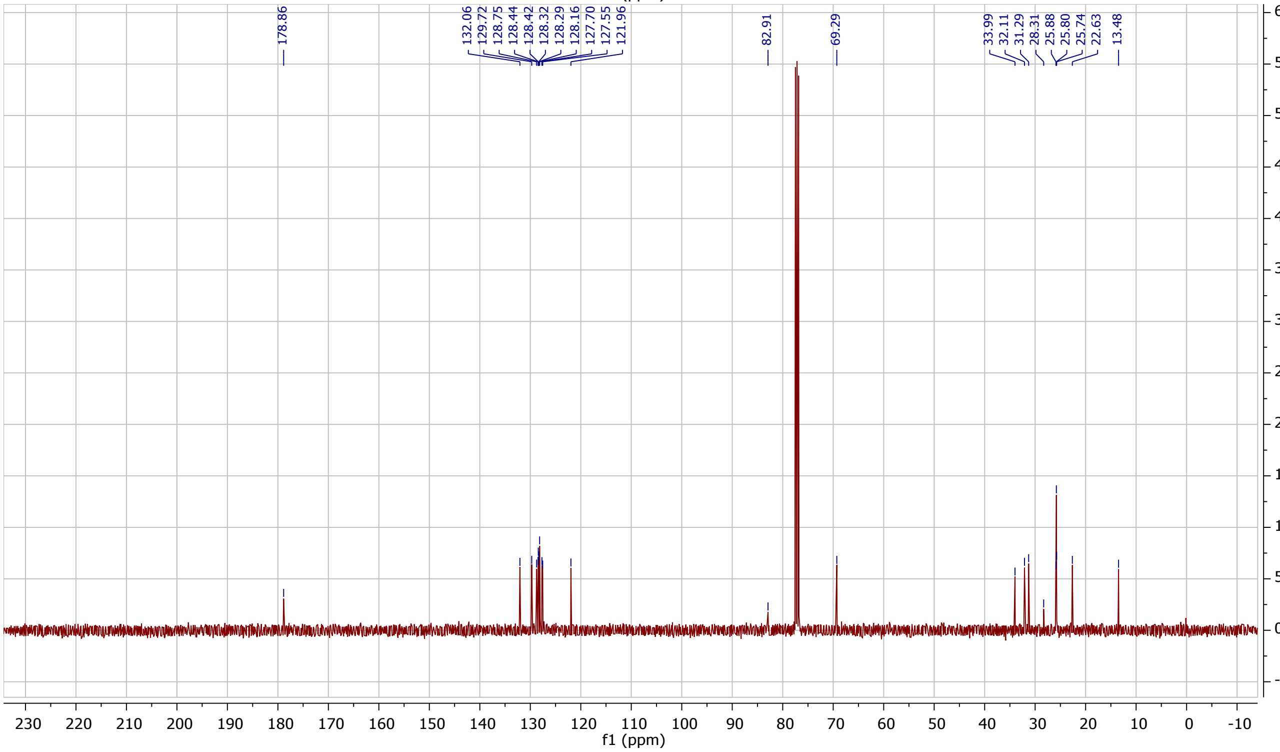
178.86
132.06 129.72 128.75 128.44 128.42 128.32 128.29 128.16 127.70 127.55 121.96
82.91
69.29
33.99 32.11 31.29 28.31 25.88 25.80 25.74 22.63 13.48
f1 (ppm)

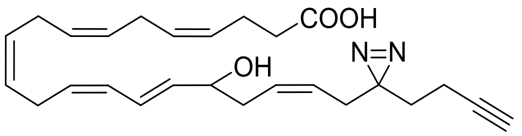

pac-17-HDHA (5)

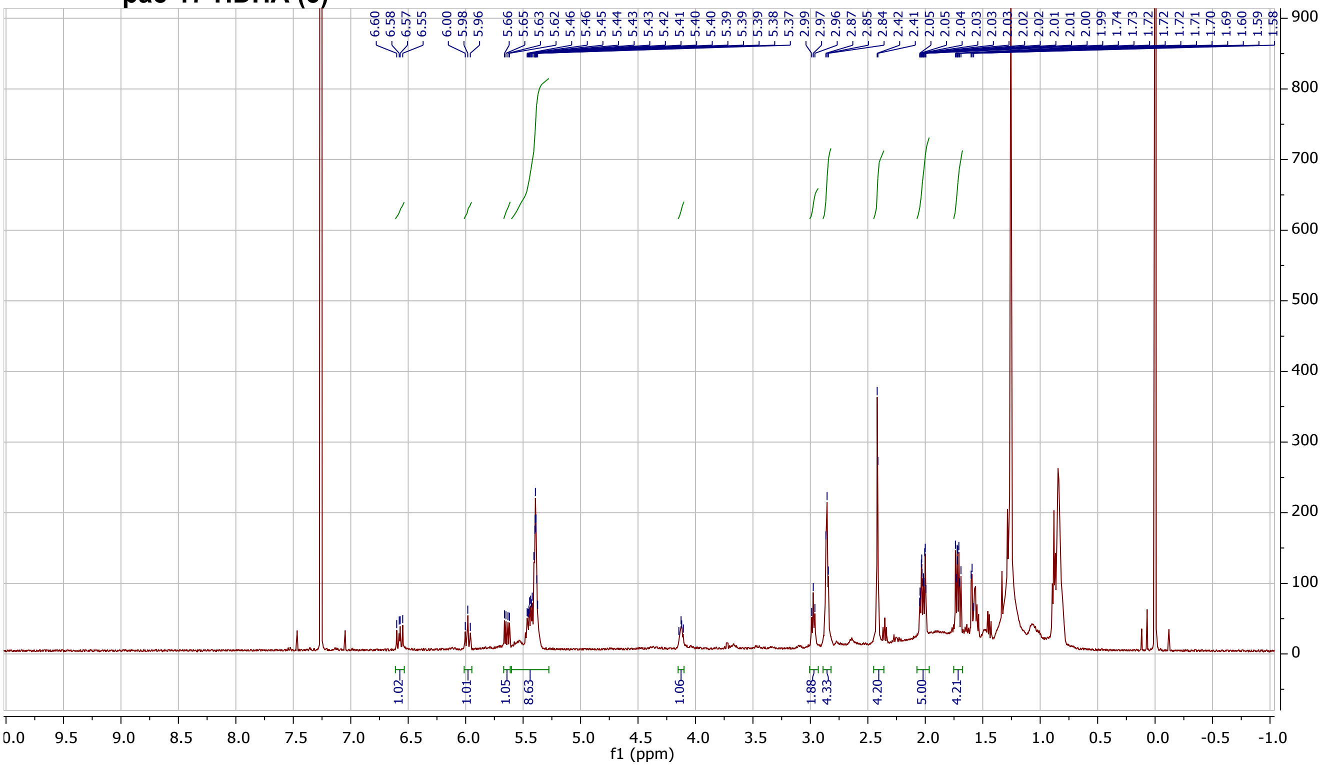

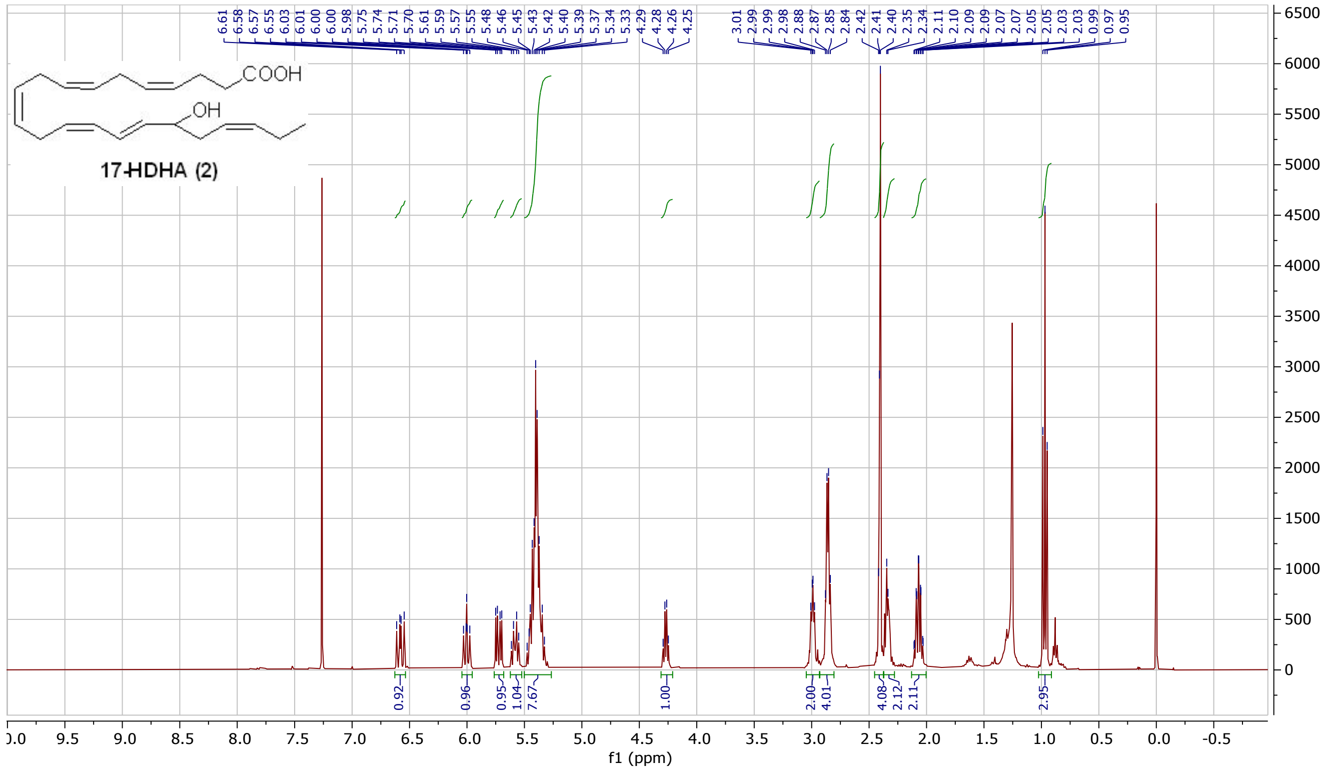

LC-MS analysis of pac-17-HDHA (5)

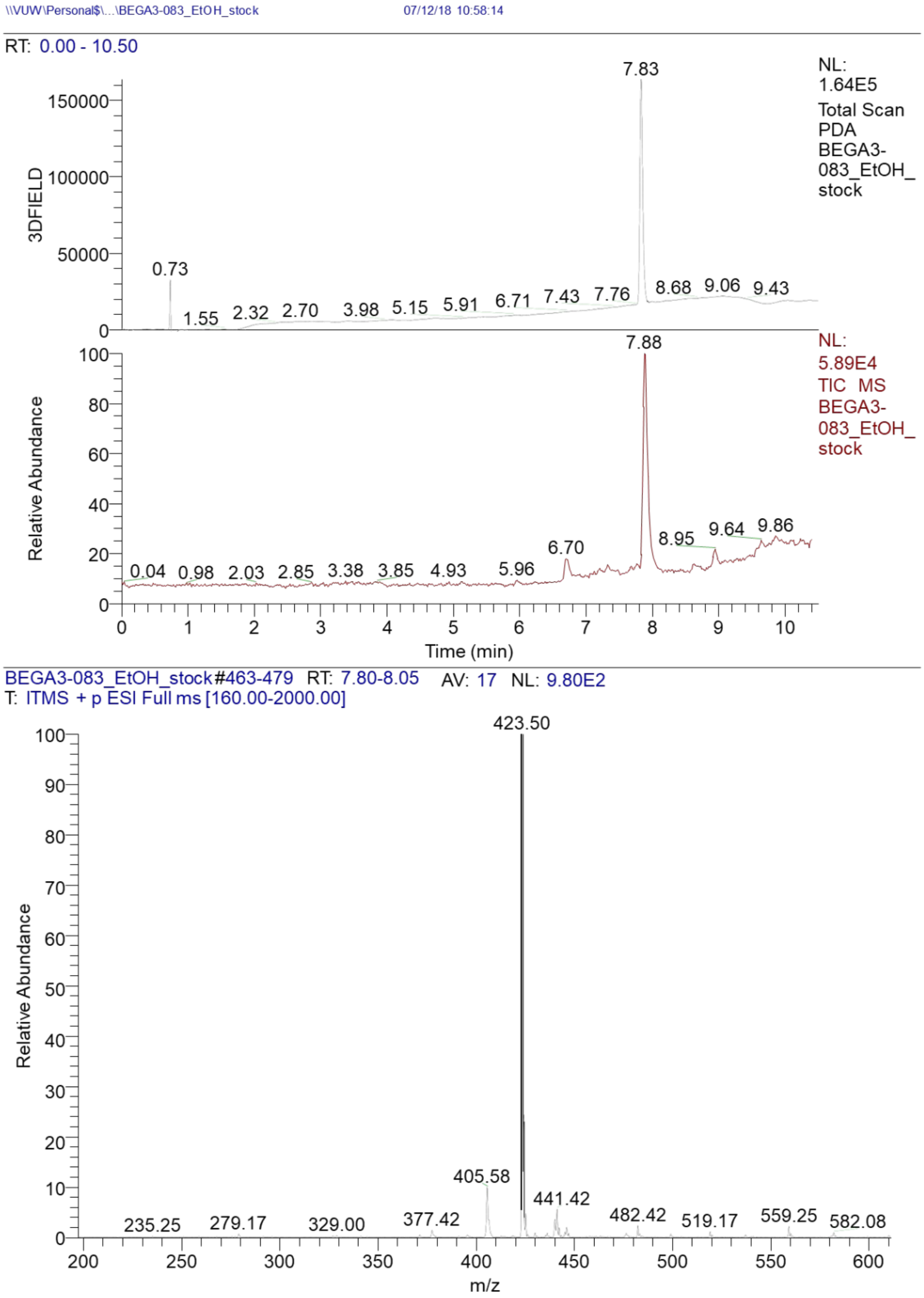

Fragmentation pattern of hydrogenated pac-17-HDHA (5)

Print of window 80: MS Spectrum

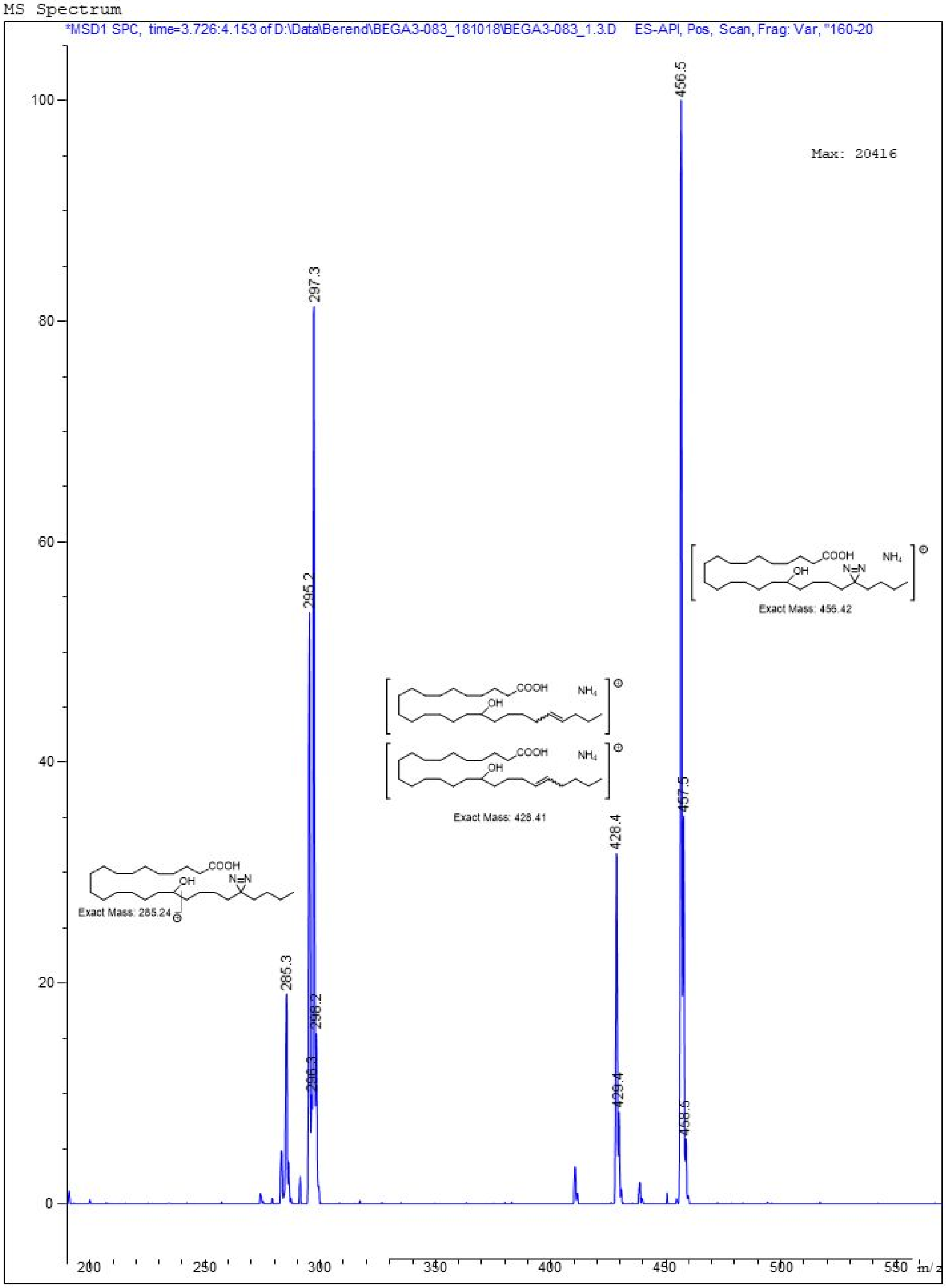

LC-MS_6120B 1/30/2019 11:19:17 AM SYSTEM Page 1 of 1

References

(1) S. T. A. Koenders, B. Gagestein, and M. van der Stelt Opportunities for Lipid-Based Probes in the Field of Immunology. In *Activity-Based Protein Profiling*; Cravatt, B. F., Hsu, K.-L., Weerapana, E., Eds.; Current Topics in Microbiology and Immunology; Springer International Publishing: Cham, 2019; pp 283–319.

(2) K. Labun, T. G. Montague, J. A. Gagnon, S. B. Thyme, and E. Valen CHOPCHOP v2: A Web Tool for the next Generation of CRISPR Genome Engineering. *Nucleic Acids Res.* **2016**, *44*, 272–276.

(3) L. Cong, F. A. Ran, D. Cox, S. Lin, R. Barretto, N. Habib, P. D. Hsu, X. Wu, W. Jiang, L. A. Marraffini, *et al.* Multiplex Genome Engineering Using CRISPR/Cas System. *Science* **2013**, *339* (February), 819–824.

(4) F. A. Ran, P. D. Hsu, J. Wright, V. Agarwala, D. A. Scott, and F. Zhang Genome Engineering Using the CRISPR-Cas9 System. *Nat. Protoc.* **2013**, *8* (11), 2281–2308.

(5) H. S. Jónasdóttir, A. Ioan-Facsinay, J. Kwekkeboom, H. Brouwers, A.-M. Zuurmond, R. Toes, A. M. Deelder, and M. Giera An Advanced LC–MS/MS Platform for the Analysis of Specialized Pro-Resolving Lipid Mediators. *Chromatographia* **2015**, *78* (5), 391–401.

(6) E. J. van Rooden, B. I. Florea, H. Deng, M. P. Baggelaar, A. C. M. van Esbroeck, J. Zhou, H. S. Overkleeft, and M. van der Stelt Mapping *in Vivo* Target Interaction Profiles of Covalent Inhibitors Using Chemical Proteomics with Label-Free Quantification. *Nat. Protoc.* **2018**, *13* (4), 752–767.

(7) M. Soethoudt, S. C. Stolze, M. V. Westphal, L. van Stralen, A. Martella, E. J. van Rooden, W. Guba, Z. V. Varga, H. Deng, S. I. van Kasteren, *et al.* Selective Photoaffinity Probe That Enables Assessment of Cannabinoid CB2 Receptor Expression and Ligand Engagement in Human Cells. *J. Am. Chem. Soc.* **2018**, *140* (19), 6067–6075.

(8) J. Rappsilber, M. Mann, and Y. Ishihama Protocol for Micro-Purification, Enrichment, Pre-Fractionation and Storage of Peptides for Proteomics Using StageTips. *Nat. Protoc.* **2007**, *2* (8), 1896–1906.

(9) S. P. Morcillo, D. Leboeuf, C. Bour, and V. Gandon Calcium-Catalyzed Synthesis of Polysubstituted 2-Alkenylfurans from β-Keto Esters Tethered to Propargyl Alcohols. *Chem. – Eur. J.* **2016**, *22* (47), 16974–16978.

(10) Z. Li, P. Hao, L. Li, C. Y. J. Tan, X. Cheng, G. Y. J. Chen, S. K. Sze, H.-M. Shen, and S. Q. Yao Design and Synthesis of Minimalist Terminal Alkyne-Containing Diazirine Photo-Crosslinkers and Their Incorporation into Kinase Inhibitors for Cell- and Tissue-Based Proteome Profiling. *Angew. Chem.* **2013**, *125* (33), 8713–8718.

(11) M. Walko, E. Hewitt, S. E. Radford, and A. J. Wilson Design and Synthesis of Cysteine-Specific Labels for Photo-Crosslinking Studies. *RSC Adv.* **2019**, *9* (14), 7610–7614.

(12) K. Ravindar, M. Sridhar Reddy, and P. Deslongchamps A Highly Efficient Access to Spiroketals, Mono-Unsaturated Spiroketals, and Furans: Hg(II)-Catalyzed Cyclization of Alkyne Diols and Triols. *Org. Lett.* **2011**, *13* (12), 3178–3181.

(13) W. Tang and E. V. Prusov Total Synthesis of RNA-Polymerase Inhibitor Ripostatin B and 15-Deoxyripostatin A. *Angew. Chem. Int. Ed.* **2012**, *51* (14), 3401–3404.

(14) A. Köpfer and B. Breit Rhodium-Catalyzed Hydroformylation of 1,1-Disubstituted Allenes Employing the Self-Assembling 6-DPPon System. *Angew. Chem. Int. Ed.* **2015**, *54* (23), 6913–6917.

(15) V. Ramella, Z. He, C. G. Daniliuc, and A. Studer Palladium-Catalyzed Dearomatizing Difunctionalization of Indoles and Benzofurans. *Eur. J. Org. Chem.* **2016**, *2016* (13), 2268–2273.
